# SCM-198 Prevents Endometriosis by Reversing Low Autophagy of Endometrial Stromal Cell *via* Balancing ERα and PR Signals

**DOI:** 10.3389/fendo.2022.858176

**Published:** 2022-06-15

**Authors:** Yi-Kong Lin, Yun-Yun Li, Yue Li, Da-Jin Li, Xiao-Lin Wang, Li Wang, Min Yu, Yi-Zhun Zhu, Jia-Jing Cheng, Mei-Rong Du

**Affiliations:** ^1^ NHC (National Health Commission) Key Lab of Reproduction Regulation (Shanghai Institute for Biomedical and Pharmaceutical Technologies), Shanghai Key Laboratory of Female Reproductive Endocrine Related Diseases, Hospital of Obstetrics and Gynecology, Fudan University, Shanghai Medical College, Shanghai, China; ^2^ Department of Obstetrics and Gynecology, Shanghai Fourth People’s Hospital, School of Medicine, Tongji University, Shanghai, China; ^3^ State Key Laboratory of Quality Research in Chinese Medicine and School of Pharmacy, Macau University of Science and Technology, Macao, Macao SAR, China; ^4^ Department of Obstetrics and Gynecology, Guangzhou First People’s Hospital, School of Medicine, South China University of Technology, Guangzhou, China

**Keywords:** SCM-198, EMS, estrogen, progesterone, TNF-α, autophagy

## Abstract

**Background:**

Endometriosis (EMS), an endocrine-related inflammatory disease, is characterized by estrogen and progesterone imbalance in ectopic lesions. However, its pathogenic mechanism has not been fully elucidated. While SCM-198 is the synthetic form of leonurine and has multiple pharmacological activities such as antioxidation and anti-inflammation, it remains unknown whether it could inhibit the progress of EMS by regulating estrogen signaling and inflammation.

**Methods:**

The therapeutic effects of SCM-198 on EMS and its potential mechanism were analyzed by establishing EMS mouse models and performing an RNA sequencing (RNA-seq) assay. ELISA was performed to detect estrogen and tumor necrosis factor (TNF) -α concentrations in normal endometrial stromal cells (nESCs) and ectopic endometrial stromal cells (eESCs) with or without SCM-198 treatment. Western blotting, RNA silencing, and plasmid overexpression were used to analyze the relationship between inflammation, endocrine factors, and autophagy and the regulatory activity of SCM-198 on the inflammation-endocrine-autophagy axis.

**Results:**

Increased estrogen-estrogen receptor (ER) α signaling and decreased progesterone receptor isoform B (PRB) expression synergistically led to a hypo-autophagy state in eESCs, which further inhibited the apoptosis of eESCs. The high expression of TNF-α in eESCs enhanced the antiapoptotic effect mediated by low autophagy through the activation of the aromatase-estrogen-ERα signaling pathway. SCM-198 inhibited the growth of ectopic lesions in EMS mice and promoted the apoptosis of eESCs both *in vivo* and *in vitro.* The apoptotic effect of SCM-198 on eESCs was attained by upregulating the autophagy level *via* the inhibition of the TNF-α-activated aromatase-estrogen-ERα signal and the increase in PRB expression.

**Conclusion:**

Inflammation facilitated the progress of EMS by disrupting the estrogen regulatory axis. SCM-198 inhibited EMS progression by regulating the inflammation-endocrine-autophagy axis.

## Introduction

Endometriosis (EMS) is defined as the presence of endometrial-like tissue outside the uterine cavity and periodic bleeding of ectopic lesions under the influence of ovarian hormones (1). EMS is an estrogen-dependent inflammatory disease that affects approximately 10% of women of childbearing age (2) and is associated with pelvic pain and infertility (3–6). None of the proposed pathogenetic mechanisms (retrograde menstruation, coelomic metaplasia, and the immune disorder theory) can fully explain the origin and development of EMS (7, 8). Recent studies indicate that the etiology of EMS is associated with the abnormality of inflammation and endocrine factors (9, 10).

There is currently no known gold standard treatment for EMS, which explains its high recurrence rate of ~50% (11). Surgery is traumatic (12, 13), and medical therapy [progesterone and gonadotropin-releasing hormone (GnRH) agonists] cannot effectively reduce estrogen production by ectopic lesions (11). Although EMS-related medical expenses are similar to those of diabetes, the medical management of the condition is still unsatisfactory. Therefore, there is an urgent need for a profound understanding of the pathogenesis of EMS and a suitable treatment scheme.

Enhanced survivability of ectopic endometria leads to the development of EMS (14). Elevated estrogen and reduced progesterone levels synergistically enhance the survival of ectopic endometria (15, 16). In addition, accumulated pro-inflammatory factors such as tumor necrosis factor (TNF)-α can promote the proliferation of ectopic endometrial stromal cells (eESCs) (17, 18). Moreover, previous meta-analyses have shown that TNF-α was associated with susceptibility to EMS, and anti-TNF-α therapy could relieve the pelvic pain associated with this condition (19, 20). However, the specific interaction between endocrine factors and inflammation in EMS needs to be studied further.

Recently, the role of autophagy in the pathogenesis of EMS has been emphasized (21). The autophagy level of eESCs is reduced, which further promotes their survival and inhibits their apoptosis (22–24). It has been found that increased estrogen signals inhibit autophagy in EMS (10, 25). Inflammation and autophagy can be negatively regulated by each other (26–29). Whether inflammation, endocrine factors, and autophagy jointly mediate the pathological process of EMS remains to be further explored.

Studies have demonstrated that SCM-198, a synthetic form of leonurine (30, 31), has therapeutic effects on cardiovascular disease (32, 33) and cerebrovascular disease (34). Importantly, SCM-198 has been demonstrated to alleviate hyperalgesia in mice with adenomyosis (35). However, no study has been reported to explore the effect of SCM-198 on EMS.

In this study, we focused on the therapeutic effects of SCM-198 on EMS and explored the regulatory roles of SCM-198 in the network of inflammation, endocrine factors, and autophagy of EMS. Here, we explained the complicated interplay between inflammation, endocrine factors, and autophagy in the pathogenesis of EMS and also presented a promising therapy for such a refractory disease.

## Materials and Methods

### Reagents and the Endometriosis Mouse Model

SCM-198 was kindly gifted by Dr. Zhu Yizhun’s laboratory. Female C57BL/6 mice (6–8 weeks old) were purchased from Shanghai JieSiJie Laboratory Animal Co. Ltd. (Shanghai, China). After 2 weeks of adaptation, the mice were randomly selected as the donors of the EMS model. Donor mice were intraperitoneally injected with 17β estradiol (E2) (#E2758, Sigma, St. Louis, MO, USA) (0.2 µg/g weight) thrice for a week as previously reported (36). Vaginal smears were used to select estrus mice as the recipients of EMS mouse models. As previously described (36), the uteri of donor mice were minced together, and then the tissue debris was intraperitoneally injected into recipient mice (the number of the donor uteri was equal to the number of the recipient mice). Since the ectopic lesions were well developed within 1 week after injection, a 7-day formulation of EMS ectopic lesions was administered in this study as previously described (37).

To investigate the effects of SCM-198 on the pathogenesis of EMS, recipient mice were randomly divided into three groups: the EMS group, the EMS+SCM-198 low-dose group (EMS+SCM-198 L, 7.5 mg/kg), and the EMS+SCM-198 high-dose group (EMS+SCM-198 H, 15 mg/kg). According to the corresponding dose (once daily for a week), a 200-µl aliquot of SCM-198 was intraperitoneally injected into each recipient mouse. The mice in the EMS group were given Phosphate buffer saline (PBS) at the same posology. One week later, all of the mice were sacrificed. The endometriotic tissue, uterus, and peritoneal fluid were collected for subsequent analyses.

### Collection of Human Samples and the Isolation of Endometrial Stromal Cells

Ectopic endometrial tissues of 46 women (aged 22–45 years) with ovarian EMS were obtained *via* laparoscopic surgery, and normal endometrial samples were collected from 10 healthy women (aged 23–46 years) by uterine curettage at the Obstetrics and Gynecology Hospital of Fudan University. The demographic and obstetrical characteristics of the enrolled participants are summarized in **Table 1**. Each sample (at least 500 mg) was collected under sterile conditions. ESCs were isolated according to a previously described method (38–41). This method yields ESCs with more than 95% purity, as confirmed by using immunocytochemical staining of vimentin.

**Table 1:** Characteristics of the study participants.

Subjects	Non-EMS	EMS	*P*
Number	10	46	–
Age range (years)	23–46	22–45	–
Mean age^a^	34.3 ± 2.28	35.3 ± 0.84	ns
Cyst diameter (cm)^b^	–	5.74 ± 0.88	–
rAFS stage [n (%)]			
I	NA	0	–
II	NA	0	–
III	NA	26 (56.52%)	–
IV	NA	20 (43.48%)	–
Menstrual cycle [n (%)]			
Proliferative phase	5 (50%)	16 (34.78%)	–
Secretory phase	5 (50%)	30 (65.22%)	–
Treatment history	–	–	–

a Mean ± standard error of the mean (SEM).

b Mean ± standard deviation (SD).

EMS, endometriosis; rAFS, revised American Fertility Society, NA, Not applicable; ns, no significant.

Briefly, the endometrial tissues were minced (2–3-mm pieces) and digested in Dulbecco's Modified Eagle's Medium (DMEM)/F-12 containing collagenase type IV (0.1%, Sigma, USA) for 30 min at 37°C. Then, the dispersed cells were filtered through a 400-mesh wire sieve to remove the undigested tissue pieces containing glandular epithelium. After gentle centrifugation, the supernatant was discarded, and the cells were resuspended in DMEM/F-12 containing 10% fetal bovine serum (Gemini, Calabasas, CA, USA), 100 IU/ml penicillin (Sigma, USA), 100 μg/ml streptomycin (Sigma, USA), and 1 μg/ml amphotericin B (Sangon, Shanghai, China) at 37°C in 5% CO_2_. Each clinical sample was an independent source of ESCs. Freshly isolated ESCs were cultured overnight in a 25-cm^2^ flask (Corning, USA) per sample. In the next day, those cells that did not adhere were washed away, and those that adhered were largely stromal cells (2–3 × 10^6^/flask), which could attain 85%–90% fusion. After trypsin digestion, ESCs were seeded into the six-well plate at a density of 3–5 × 10^5^/well for further experiments.

### Immunohistochemistry

The immunohistochemical sections were kept at 60°C for 2 h. Xylene and gradient alcohol were used to dewax and rehydrate the sections. The sections were incubated with 3% hydrogen peroxide and 5% bovine serum albumin successively to block endogenous peroxidase. Tissue sections were incubated with anti-mouse estrogen receptor (ER)α (#ab32063, Abcam, Cambridge, UK) and progesterone receptor (PR) (#ab101688, Abcam, UK) overnight in a humid chamber at 4°C. The sections were washed thrice with PBS for 5 min each time and covered with peroxidase-conjugated goat anti-rabbit or mouse IgG (#GK500710, Gene Teck, San Francisco, CA, USA) for 30 min. Next, they reacted with 3,3-diaminobenzidine (DAB), and the nucleus was stained with hematoxylin. Finally, the slices were dehydrated in gradient alcohol and xylene and then mounted.

### Western Blotting Analysis

The total proteins of ESCs, mouse uterine tissue, and ectopic lesions were extracted by a radioimmunoprecipitation assay (RIPA) buffer (Beyotime, Shanghai, China) supplemented with protease and phosphatase inhibitors (Sigma, USA). The protein concentration was measured using a BCA (Bicinchoninic Acid) protein assay kit (Beyotime, China). After denaturation, equal amounts of protein were separated *via* Sodium dodecyl sulfate (SDS)-polyacrylamide gel electrophoresis (PAGE) and then wet-transferred to polyvinylidene difluoride membranes. Nonspecific binding sites were blocked by incubating the membranes with 5% skim milk in Tris-buffered saline with 0.1% Tween 20 (TBS-T) for 1 h. Next, the membranes were incubated overnight at 4°C with primary antibodies (1:1,000) against aromatase (#14528, CST, Boston, USA), ERα (#ab32063, Abcam, UK), PRB (#ab32085, Abcam, UK), LC3B (#3868, CST, USA), BECN1 (#ab207612, Abcam, UK), Bcl-2 (#2870, CST, USA), Bax (#12105, CST, USA), FN1 (#ab2413, Abcam, UK), vimentin (#5741, CST, USA), α-tubulin (#ab7291, Abcam, UK), and glyceraldehyde-3-phosphate dehydrogenase (GAPDH) (#10112, Arigo, Taiwan, China). Subsequently, membranes were incubated with appropriate horseradish peroxidase (HRP) -conjugated anti-rabbit (#65351, Arigo, China) or anti-mouse (#65350, Arigo, China) IgG secondary antibodies for 1 h at room temperature. After washing with TBS-T thrice, the immunopositive bands on the blots were visualized on the enhanced chemiluminescence detection system (Merck Millipore, USA) using chemiluminescent HRP substrate (#WBKLS0100, Millipore, Boston, MA, USA).

### RNA Sequencing Data Analysis

The corrected expression value of genes or transcriptomes, the corrected value of the fold change, the *P*-value, and the false discovery rate (FDR) value were obtained by DESeq2. We considered transcripts as differentially expressed if the *P*-value was <0.05 and the fold change was either >1.2 or <0.83333. The GO (Gene Ontology) and KEGG (Kyoto Encyclopedia of Genes and Genomes) databases were used for functional enrichment and pathway enrichment, respectively. Bubble charts and volcano plot were produced using the ggplot and cluster profiler packages of R version 4.0.4.

### Quantitative Real-Time PCR

Total RNA was extracted using TRIzol reagent (Invitrogen, CA, USA) and then reverse-transcribed into first-strand complementary DNA (cDNA) (Takara, Kyoto, Japan) per the manufacturer’s instructions. The synthesized cDNA was amplified using the ABI PRISM 7900 Sequence Detection System (Applied Biosystems, CA, USA) with specific primers and SYBR Green (Takara, Japan). Triplicate samples were examined for each condition. A comparative threshold cycle value was normalized for each sample using the *2^-ΔΔ^* Ct method.

### ELISA

The supernatants of normal endometrial stromal cells (nESCs) and differently treated eESCs were harvested and assayed by ELISA per the manufacturer’s instructions (estrogen, #CSB-E07286h, CUSABIO, Shanghai, China; TNF-α, #BDEL-0049, Biodragon, Beijing, China) to detect the secretion levels of estrogen and TNF-α.

### Plasmid Overexpression and siRNA Transfection

The aromatase overexpression (Aromatase^over^) plasmid and negative control plasmid were purchased from Shanghai Genechem Co., Ltd. (Shanghai, China). Aromatase siRNA (siAromatase) and control siRNA were purchased from Shanghai Genepharma Co., Ltd. (Shanghai, China). The Aromatase^over^ plasmid and negative control plasmid (Ctrl) were transfected into eESCs by liposome transient transfection when the fusion degree reached approximately 70%–80% in a six-well plate. Transfected cells were incubated at 37°C for 24 h and then collected for further study. The transfection process of aromatase-silencing (siAromatase) was similar to that of the overexpressed aromatase transfection.

### Statistical Analysis

Prism 8 (GraphPad) was used for data analysis. Statistical significance was determined by either Student’s t-test for two-group analyses or the one-way ANOVA for multiple group comparisons. Continuous data were presented as the mean ± SD. The threshold for statistical significance was set at *P* < 0.05.

## Results

### SCM-198 Suppresses Endometriotic Growth Both *In Vivo* and *In Vitro*

**Figure 1:**
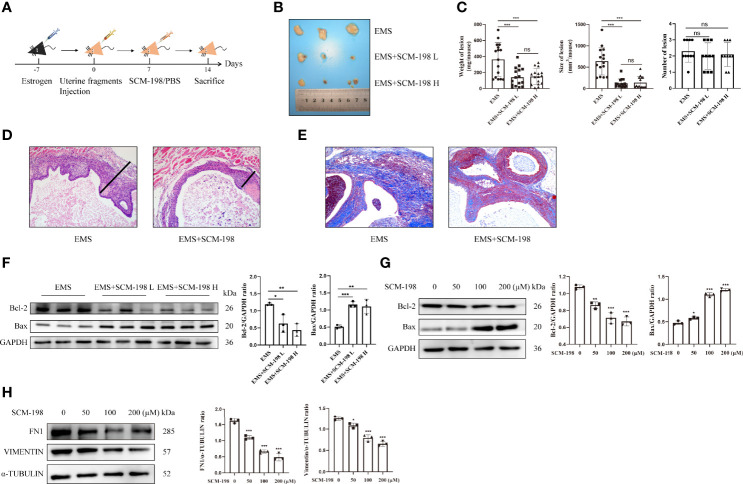
SCM-198 suppresses endometriotic growth both *in vivo* and *in vitro* EMS mice were treated with or without SCM-198 at low (EMS+SCM-198 L, 7.5 mg/kg) or high (EMS+SCM-198 H, 15 mg/kg) doses once daily for 1 week. **(A)** The flowchart of the process of establishing the mouse EMS model. **(B)** Representative images of the ectopic lesions from EMS mice. **(C)** Quantitative results for the weight (n = 16), size (n = 15), and number (n = 10) of ectopic lesions from EMS mice. **(D)** Thicknesses of ectopic cyst walls from EMS mice. **(E)** Masson staining was performed to detect collagen fibers of ectopic lesions. **(F)** Western blotting was utilized to analyze the protein levels of Bcl-2 and Bax in ectopic lesions (n = 3). **(G, H)** The eESCs from EMS patients were treated with different doses of SCM-198 for 48 h, and then Western blotting was used to analyze the protein levels of Bcl-2, Bax, FN1, and vimentin (n = 3). Continuous data are presented as the mean ± SD (*P < 0.05, **P < 0.01, and ***P < 0.001; ns, not significant).

Firstly, we used mouse models to investigate whether SCM-198 could alleviate the development of EMS. **Figure 1A** illustrates the general process of establishing EMS mouse models. As shown in **Figures 1B**, **C**, SCM-198 significantly decreased the weights and sizes of mouse ectopic lesions. However, we found no significant difference in the number of ectopic lesions in EMS mice treated with or without SCM-198 (**Figure 1C**). Then, we separated whole ectopic lesions from mice and stained the sections with hematoxylin and eosin (H&E). Through microscopic observation, we found that the outer layers of ectopic lesions were coated with fibrotic tissue. We then measured the thickness of the surrounding fibrotic tissue under the microscope and found that SCM-198 reduced the wall thickness of EMS lesions (**Figure 1D**). In addition, Masson staining revealed that SCM-198 significantly reduced collagen accumulation in ectopic lesions (**Figure 1E**). Western blotting results revealed that SCM-198 inhibited the expression of antiapoptotic protein Bcl-2 and promoted the expression of proapoptotic protein Bax in ectopic lesions at both low and high doses (**Figure 1F**). In line with *in vivo* results, *in vitro* analyses revealed that SCM-198 inhibited Bcl-2 and promoted Bax expression in human eESCs (**Figure 1G**). Meanwhile, the levels of fibrosis-related molecules such as fibronectin 1 (FN1) and vimentin were also reduced in human eESCs after SCM-198 treatment (**Figure 1H**). These results suggest that SCM-198 is able to accelerate apoptosis and attenuate the growth and fibrosis of EMS both *in vivo* and *in vitro*.

### SCM-198 Promotes the Autophagy Level and Reverses the ERα/PR Imbalance of Endometriosis

**Figure 2:**
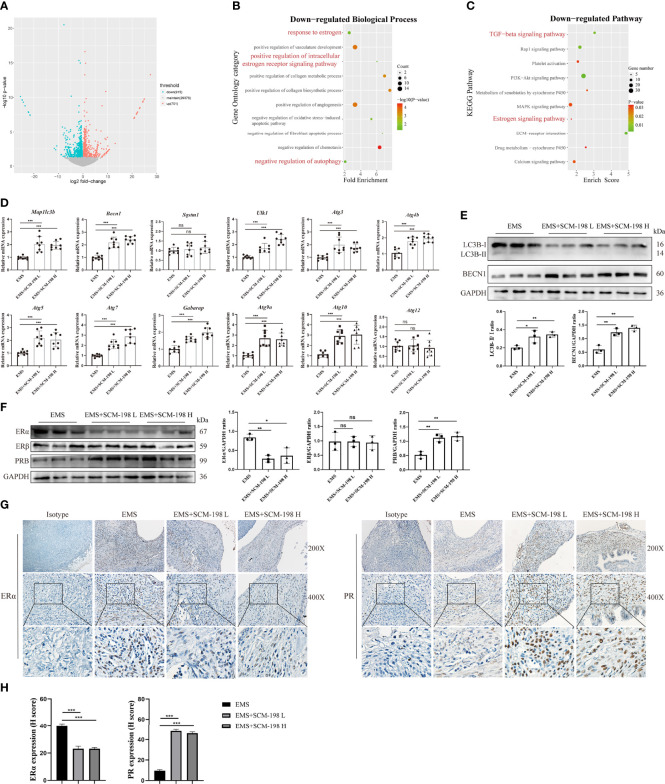
SCM-198 promotes the autophagy level and reverses the ERα/PR imbalance of EMS **(A)** A volcano plot of differentially expressed genes of ectopic lesions in SCM-198-treated EMS mice compared with those from the untreated EMS mice. The x-axis shows the Log2 (fold change) in expression, and the y-axis shows the -Log10 (*P*-value) of the gene being expressed differentially (blue: downregulated genes; red: upregulated genes). **(B)** GO enrichment of biological process for the downregulated genes. **(C)** The KEGG pathway analysis of the downregulated genes. **(D)** The mRNA expressions of Map1lc3b, Becn1, Sqstm1, Ulk1, Atg3, Atg4b, Atg5, Atg7, Gabarap, Atg9a, Atg10, and Atg12 of the ectopic lesion (n = 8) treated with or without SCM-198. **(E, F)** The protein expressions of LC3B-II/I, BECN1, ERα, Erβ, and PR in ectopic lesions were measured *via* Western blotting (n = 3). **(G, H)** The representative IHC images and quantification data of ERα and PR expressions in ectopic lesions. Data are presented as the mean ± SD (*P < 0.05, **P < 0.01, and ***P < 0.001; ns, not significant).

To investigate the underlying mechanism of SCM-198 in restraining EMS, we performed RNA-seq in ectopic lesions of EMS mice that were either treated with SCM-198 or not. We observed a total of 1,616 differentially expressed genes, with 701 genes being upregulated and 915 genes being downregulated in SCM-198-treated ectopic lesions (**Figure 2A**). GO enrichment and KEGG pathway analyses revealed that SCM-198 reduced the levels of autophagy inhibitor molecules and inhibited the ER pathway in ectopic lesions (**Figures 2B, C** **)**.

To confirm the results of the bioinformatics analysis, we first analyzed the expression of autophagy-related genes in ectopic lesions. The results presented in **Figure 2D** show that SCM-198 could extensively promote the mRNA expression of autophagy-related proteins such as Map1lc3b, Becn1, Ulk1, Atg3, Atg4b, Atg5, Atg7, Gabarap, Atg9a, and Atg10 in ectopic lesions. Furthermore, Western blotting results confirmed that SCM-198 could promote autophagy by increasing the ratio of LC3B-II/I and BECN1 expression (**Figure 2E**). Meanwhile, SCM-198 reversed the imbalance of ERα and PR in EMS ectopic lesions by upregulating PR and downregulating ERα expressions (**Figures 2F–H**). However, the expression of ERβ was not significantly decreased by SCM-198 (**Figure 2F**). These results indicate that SCM-198 could promote autophagy and reverse the imbalance of ERα/PR in EMS.

### The ERα/PR Imbalance Contributes to the Hypo-Autophagy State of Ectopic Endometrial Stromal Cells

**Figure 3:**
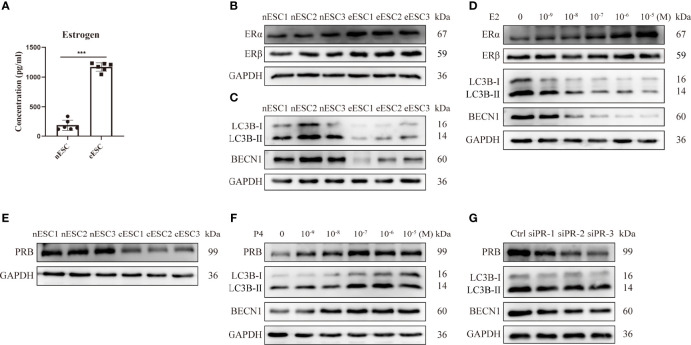
The ERα/PR imbalance contributes to the hypo-autophagy state of eESCs. **(A)** ELISA was used to detect the estrogen concentrations of eESCs and nESCs (n = 6), both of which were cultured in culture flasks for 24 h. **(B, C)** The protein expressions of ERα, ERβ, LC3B-II/I, and BECN1 of eESCs and nESCs were analyzed *via* Western blotting. **(D)** After treatment with different concentrations of E2 for 48 h, the expression levels of ERα, ERβ, LC3B-II/I, and BECN1 in eESCs were analyzed *via* Western blotting. **(E)** The protein expressions of PRB in eESCs and nESCs were detected *via* Western blotting. **(F)** After treatment with different concentrations of progesterone for 48 h, the protein levels of PRB, LC3B-II/I, and BECN1 in eESCs were analyzed *via* Western blotting. **(G)** The protein levels of PRB, LC3B-II/I, and BECN1 in eESCs after PR silencing were analyzed *via* Western blotting. Continuous data are presented as the mean ± SD (***P < 0.001).

Then, we assessed the levels of estrogen, hormone receptors, and autophagy in eESCs from EMS patients. Higher production of estrogen (**Figure 3A**) and upregulated ERα (**Figure 3B**) were observed in eESCs. Compared with nESCs, LC3B-II/I and BECN1 were downregulated in eESCs, indicating a lower autophagy level in eESCs (**Figure 3C**). To explore the relationship between estrogen signaling and autophagy, eESCs were treated with E2. The results showed that E2 treatment dose-dependently increased ERα and inhibited autophagy by reducing LC3B-II/I and BECN1 (**Figure 3D**). Previous studies have demonstrated that ERα inhibited autophagy in eESCs (10). Thus, high local estrogen production led to an increase in ERα, which further inhibited autophagy. Progesterone resistance in the ectopic endometrium is mainly mediated by the decrease in PRB (the isoform of PR). Therefore, we focused on the effect of SCM-198 on the expression of PRB in eESCs and found that PRB was decreased in eESCs (**Figure 3E**). Progesterone increased PRB expression and promoted autophagy in a dose-dependent manner (**Figure 3F**). In addition, PR silencing downregulated the autophagy of eESCs by decreasing LC3B-II/I and BECN1, implying that the decrease in PR contributed to the hypo-autophagy state of eESCs (**Figure 3G**). Together, these results suggest that high local estrogen levels lead to increased ERα expression, and the ERα/PRB imbalance in ectopic lesions promotes hypo-autophagy in eESCs.

### SCM-198 Promotes the Autophagy-Mediated Apoptosis of Ectopic Endometrial Stromal Cells by Inhibiting the Estrogen-ERα Pathway and Promoting PR Expression

**Figure 4:**
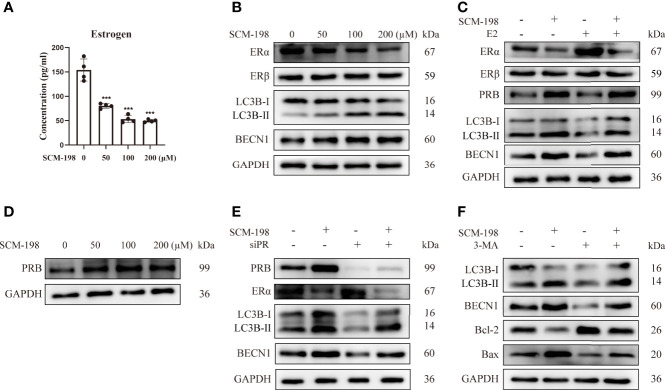
SCM-198 promotes the autophagy-mediated apoptosis of eESCs by inhibiting the estrogen-ERα pathway and promoting PR expression. **(A)** After treatment with different concentrations of SCM-198 for 48 h, the estrogen concentration of eESCs was analyzed *via* ELISA (n = 4). **(B)** The eESCs were treated with different concentrations of SCM-198 for 48 h, and then protein expressions of ERα, ERβ, LC3B-II/I, and BECN1 were analyzed *via* Western blotting. **(C)** The protein expressions of ERα, ERβ, PRB, LC3B-II/I, and BECN1 were detected *via* Western blotting in eESCs treated with SCM-198 (200 µM), E2 (100 nM), or SCM-198+E2 (200 µM, 100 nM) for 48 h. **(D)** The eESCs were treated with different concentrations of SCM-198 for 48 h, after which the protein expression of PRB was analyzed *via* Western blotting. **(E)** After treatment with SCM-198 (200 µM), silenced PR, or SCM-198+silenced PR for 48 h, the protein expressions of PRB, ERα, LC3B-II/I, and BECN1 in eESCs were detected *via* Western blotting. **(F)** After treatment with SCM-198 (200 µM), 3-MA (5 mM), or SCM-198+3-MA (200 µM, 5 mM) for 48 h, the expressions of LC3B-II/I, BECN1, Bcl-2, and Bax in eESCs were analyzed *via* Western blotting. Continuous data are presented as the mean ± SD (***P < 0.001; compared with eESCs that were not treated with SCM-198).

To investigate whether SCM-198 could promote autophagy by inhibiting estrogen signaling, we treated eESCs with SCM-198. The results showed that SCM-198 downregulated the estrogen level and ERα expression in a dose-dependent manner (**Figures 4A, B** **)** and enhanced autophagy by upregulating LC3B-II/I and BECN1 levels of eESCs. Meanwhile, no significant change in the expression of ERβ was detected (**Figure 4B**). Importantly, augmented ERα expression and the inhibited autophagy induced by E2 were reversed by SCM-198 in eESCs (**Figure 4C**). In addition, SCM-198 dose-dependently upregulated PRB expression (**Figure 4D**) and reversed the inhibitory autophagy mediated by PR silencing (**Figure 4E**). Furthermore, by using autophagy inhibitor 3-MA (3-Methyladenine), we proved that low autophagy levels were conducive to the antiapoptosis of ESCs (**Figure 4F**). Also, SCM-198 exerted proapoptotic effects on eESCs by promoting autophagy (**Figure 4F**).

Together, these results imply that SCM-198 can enhance autophagy by inhibiting the estrogen pathway and promoting PRB expression, which promotes the apoptosis of eESCs.

### TNF-α Promotes an Imbalance of Estrogen and Progesterone Signaling in Ectopic Endometrial Stromal Cells

**Figure 5:**
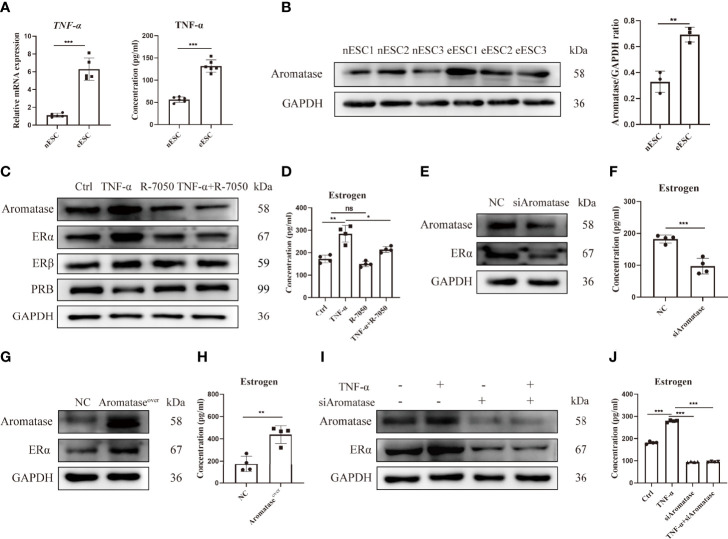
TNF-α promotes an imbalance of estrogen and progesterone signaling in eESCs. **(A)** The mRNA expression (n = 5) and concentration (n = 6) of TNF-α in eESCs and nESCs were detected *via* RT-PCR and ELISA. **(B)** The aromatase expression of eESCs and nESCs was detected *via* Western blotting (n = 3). **(C, D)** After treatment with TNF-α (10 ng/ml), R-7050 (5 µM), or TNF-α+R-7050 (10 ng/ml, 5 µM), the expressions of aromatase, ERα, Erβ, and PRB in eESCs were analyzed *via* Western blotting **(C)**, and the concentration of estrogen was detected *via* ELISA (n = 4) **(D)**. **(E–H)** Aromatase was silenced or overexpressed in eESCs for 48 h. Then, the expressions of aromatase and ERα were detected *via* Western blotting **(E, G)**, and the level of estrogen was assayed *via* ELISA (n = 4) **(F, H)**. **(I, J)** The protein expressions of aromatase and ERα were analyzed *via* Western blotting **(I)**, and the concentration of estrogen was detected *via* ELISA (n = 4) **(J)**. Continuous data are presented as the mean ± SD (*P < 0.05, **P < 0.01, and ***P < 0.001; ns, not significant).

Disordered inflammation and endocrine factors promoted the growth of ectopic lesions in EMS. To study the association between inflammation and endocrine signals in EMS, we first detected the level of the pro-inflammation cytokine TNF-α and the expression of aromatase (a key enzyme of estrogen production) in eESCs. As shown in **Figure 5A**, the mRNA expression and concentration of TNF-α were significantly increased and the expression of aromatase was also upregulated in eESCs (**Figure 5B**). Next, eESCs were treated with TNF-α or R-7050, a tumor necrosis factor receptor (TNFR) antagonist. TNF-α significantly promoted estrogen signaling by increasing aromatase and ERα levels (**Figure 5C**) and elevating estrogen concentration (**Figure 5D**). Although TNF-α and R7050 had no significant effect on the expression of ERβ, TNF-α inhibited the expression of PRB, suggesting that it aggravated the endocrine disorder by inhibiting progesterone signaling (**Figure 5C**). While aromatase silencing decreased estrogen levels and ERα expression (**Figures 5E, F** **)**, its overexpression had the opposite effect (**Figures 5G, H** **)**. The results indicated that aromatase-estrogen signaling positively regulated ERα. To demonstrate whether TNF-α positively regulates estrogen-ERα signaling through aromatase, we silenced aromatase under the treatment of TNF-α. As shown in **Figures 5I**, **J**, aromatase silencing wiped out the promotive effect of TNF-α on estrogen production and ERα expression. These data indicate that TNF-α can upregulate the aromatase-estrogen-ERα pathway and reduce PRB expression. Inflammatory disorders can promote an imbalance of estrogen and progesterone signals and, thus, accelerate the development of EMS.

### The Proapoptotic Effects of SCM-198 Are Realized by Downregulating the Aromatase-Estrogen Pathway *via* the Inhibition of TNF-α

**Figure 6:**
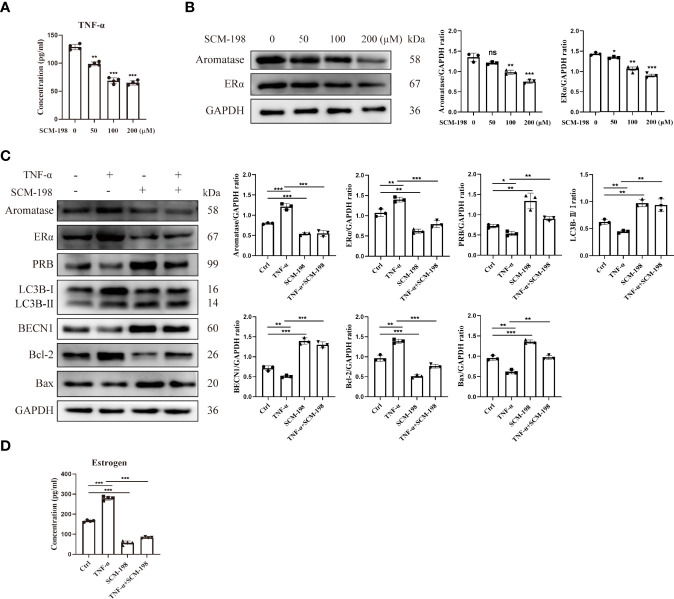
The proapoptotic effects of SCM-198 are brought about by downregulating the aromatase-estrogen pathway *via* the inhibition of TNF-α. **(A)** ELISA was utilized to detect the TNF-α concentration of eESCs treated with different doses of SCM-198 (n = 4). **(B)** The protein expressions of aromatase and ERα in eESCs treated with different doses of SCM-198 were measured *via* Western blotting (n = 3). **(C, D)** After treatment with TNF-α (10 ng/ml), SCM-198 (200 µM), or TNF-α+SCM-198 (10 ng/ml, 200 µM), the expressions of aromatase, ERα, PRB, LC3B-II/I, BECN1, Bcl-2, and Bax were analyzed *via* Western blotting (n = 3) **(C)**, and the concentration of estrogen was detected *via* ELISA (n = 4) **(D)**. Continuous data are presented as the mean ± SD (*P < 0.05, **P < 0.01, and ***P < 0.001; ns, not significant).

**Figure 7:**
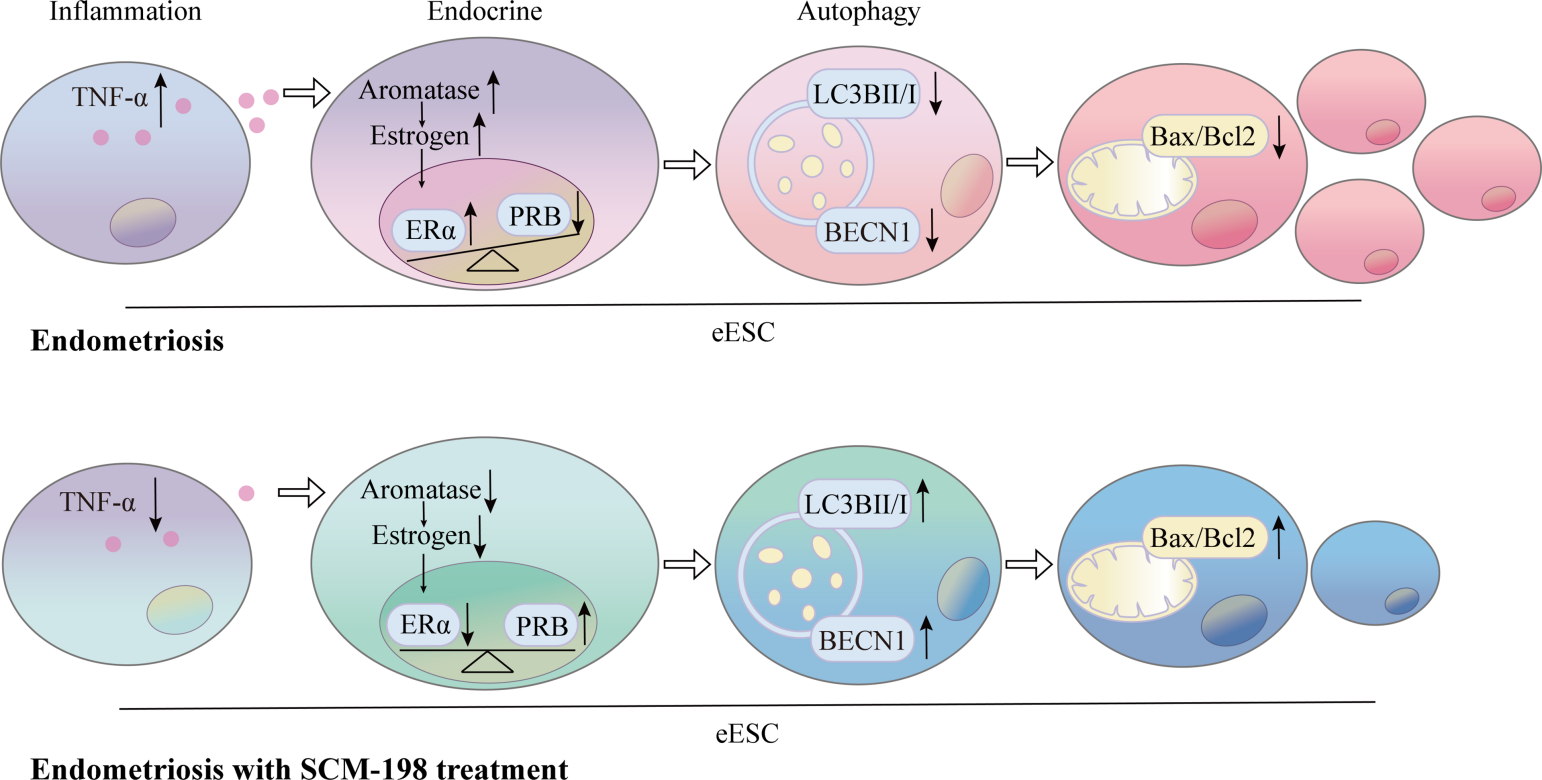
Schematic showing the therapeutic mechanism of SCM-198 on EMS. The production of TNF-α was higher in eESCs than that in nESCs. Elevated TNF-α levels augmented the activation of aromatase-estrogen-ERα signaling and aggravated PRB reduction. The upregulated estrogen signaling and downregulated progesterone signaling synergistically suppressed the autophagy level, which further led to the growth of eESCs. SCM-198 inhibited aromatase-estrogen-ERα signaling and increased PRB expression by downregulating TNF-α. Consequently, SCM-198 promoted the autophagy-mediated apoptosis of eESCs by reconstructing the balance of estrogen and progesterone signals.

To investigate whether SCM-198 can promote autophagy by inhibiting the TNF-α-mediated imbalance of estrogen and progesterone signals, we treated eESCs with different concentrations of SCM-198. SCM-198 significantly decreased the concentration of TNF-α (**Figure 6A**) and the expression of aromatase (**Figure 6B**). Furthermore, SCM-198 ameliorated aromatase-estrogen-ERα signaling and augmented PRB levels by inhibiting TNF-α (**Figures 6B–D**). The antiapoptotic effect mediated by the TNF-α-estrogen/progesterone signaling-low autophagy axis could be abated by SCM-198 (**Figures 6C, D** **)**. These results suggest that inflammation suppresses autophagy *via* estrogen and progesterone signaling, thereby inhibiting the apoptosis of eESCs. SCM-198 could restore the balance of estrogen and progesterone signaling by reducing TNF-α and, eventually, promote autophagy and accelerate the apoptosis of eESCs (**Figure 7**).

## Discussion

EMS, a common condition in obstetrics and gynecology, is characterized by the growth of endometrial tissue outside the uterine cavity. Owing to the occurrence of severe complications (pelvic pain and infertility) and the high prevalence rate (~10%), multiple therapies have been proposed for the disease (42, 43). However, a recurrence rate of up to 50% is still observed in EMS patients because neither medication nor surgery is effective in stopping the growth of these ectopic lesions (11). The search for effective medications for EMS helps to improve the quality of women’s lives and relieve a substantial economic burden. SCM-198, a synthetic form of leonurine, has been demonstrated to have the pharmacological effect of relieving pain in adenomyosis (35). In the present study, we identified the therapeutic effects of SCM-198 on EMS, as evidenced by the decreased weights and sizes of the lesions, the reduced collagen accumulation, and the increased Bax/Bcl-2 ratio in ectopic lesions of mice.

Aberrant autophagy has stepped into the spotlight of the study of EMS pathogenesis (21, 44). Accumulatively, decreased autophagy levels of ectopic endometria have been reported (45, 46). More importantly, low autophagy levels contribute to the survival of ectopic endometria, as corroborated by enhanced apoptosis and the decreased proliferation of eESCs (24, 47). Then, we investigated whether autophagy was involved in the mechanisms of SCM-198 in treating EMS. The results of RNA-seq based on ectopic foci showed that SCM-198 significantly promoted the autophagy of eESCs. The upregulation of autophagy-related factors under SCM-198 treatment further confirmed its promotive effects on eESC autophagy.

The imbalanced endocrine microenvironment of the ectopic endometrium, which is manifested through high estrogen signaling and progesterone resistance, has an inescapable responsibility for the growth of the ectopic endometrium. Specifically, increased estrogen production supported by elevated aromatase potentiates the proliferation of ectopic lesions (48–50). Progesterone resistance results from PRB (the isoform of PR) reduction, which reinforces the activation of estrogen signaling and is beneficial for EMS development (51–54). To tackle the pathogenetic mechanisms of EMS, multiple studies have emphasized that autophagy inhibition is an important pathway for estrogen to restrain apoptosis and facilitate the growth of ectopic lesions (10, 25). The promotive effect of SCM-198 on autophagy is brought about by repairing the damaged hormonal endocrine networks. We verified that increased estrogen signaling and impaired progesterone signaling synergistically led to the decline of autophagy in eESCs. Consistent with the hypothesis, SCM-198-induced upregulation of autophagy was mediated by increasing PRB expression and decreasing aromatase-estrogen-ERα signaling in eESCs.

The current consensus is that dysregulated pelvic inflammation plays a crucial role in EMS (55), as evidenced by the fact that increased TNF-α levels are closely associated with the pelvic pain and infertility caused by EMS (20, 56). Our findings confirmed that the significant upregulation of TNF-α in eESCs could be suppressed by SCM-198, which has been identified as an anti-inflammatory drug. More importantly, we demonstrated that TNF-α augmented the activation of the estrogen-ERα signal (by increasing aromatase levels) and aggravated PRB reduction. Furthermore, TNF-α decreased the autophagy of eESCs by promoting estrogen signaling and inhibiting progesterone signaling, which suggests that the inflammation-endocrine-autophagy axis plays a pivotal role in the survival of ectopic endometrium. Notably, we found that SCM-198 could reverse the low autophagy by repairing the TNF-α-induced imbalance of estrogen and progesterone and, ultimately, promote the apoptosis of eESCs.

In a nutshell, the disordered inflammation-endocrine-autophagy network was implicated in the pathogenesis of EMS. SCM-198 worked to rectify the aberrant inflammation-endocrine-autophagy axis by reversing the low autophagy level of eESCs *via* the inhibition of the TNF-α-aromatase-estrogen-ERα pathway and the promotion of PR expression. This study provided a theoretical basis for the potential application of SCM-198 in the treatment of EMS.

## Data Availability Statement

The data generated and/or analyzed in this study are available from the corresponding author on reasonable request. The original data for RNA-seq can be found at https://www.jianguoyun.com/p/DU6KkB8Q8dKuChiHjrUEIAA.

## Ethics Statement

Our study was approved by the Research Ethics Committee of the Obstetrics and Gynecology Hospital of Fudan University, and all experiments were performed per the relevant guidelines and regulations (No. Kyy2016-4) (Shanghai, China).

## Author Contributions

Y-KL and Y-YL designed and performed the experiments and drafted the article. YL searched the relevant literature, analyzed the data, and revised the article. LW, D-JL, X-LW, and MY performed data interpretation and revised the article. M-RD, J-JC, and Y-ZZ conceived the project, analyzed the data, and revised the article. All authors reviewed the article and approved its final version.

## Funding

This study was supported by grants from the National Basic Research Program of China (2021YFE0206500), the National Natural Science Foundation of China (31970859, 81630036, 91542116), the international cooperation project between Macau and Shanghai (20410760300), the Strategic Collaborative Research Program of the Ferring Institute of Reproductive Medicine (FIRMA200504), Innovation-oriented Science, and a Technology Grant from the NHC Key Laboratory of Reproduction Regulation (CX2017-2), the Innovative research team of high-level local universities in Shanghai, and a key laboratory program of the Education Commission of Shanghai Municipality (ZDSYS14005).

## Conflict of Interest

The authors declare that the research was conducted in the absence of any commercial or financial relationships that could be construed as a potential conflict of interest.

## Publisher’s Note

All claims expressed in this article are solely those of the authors and do not necessarily represent those of their affiliated organizations, or those of the publisher, the editors and the reviewers. Any product that may be evaluated in this article, or claim that may be made by its manufacturer, is not guaranteed or endorsed by the publisher.
