# Supplementary figures and images for: SCM-198 Prevents Endometriosis by Reversing Low Autophagy of Endometrial Stromal Cell via Balancing ERα and PR Signals
Source: Front Endocrinol (Lausanne). 2022 Jun 15;13:858176. doi: 10.3389/fendo.2022.858176 (PMC9245568; doi:10.3389/fendo.2022.858176)

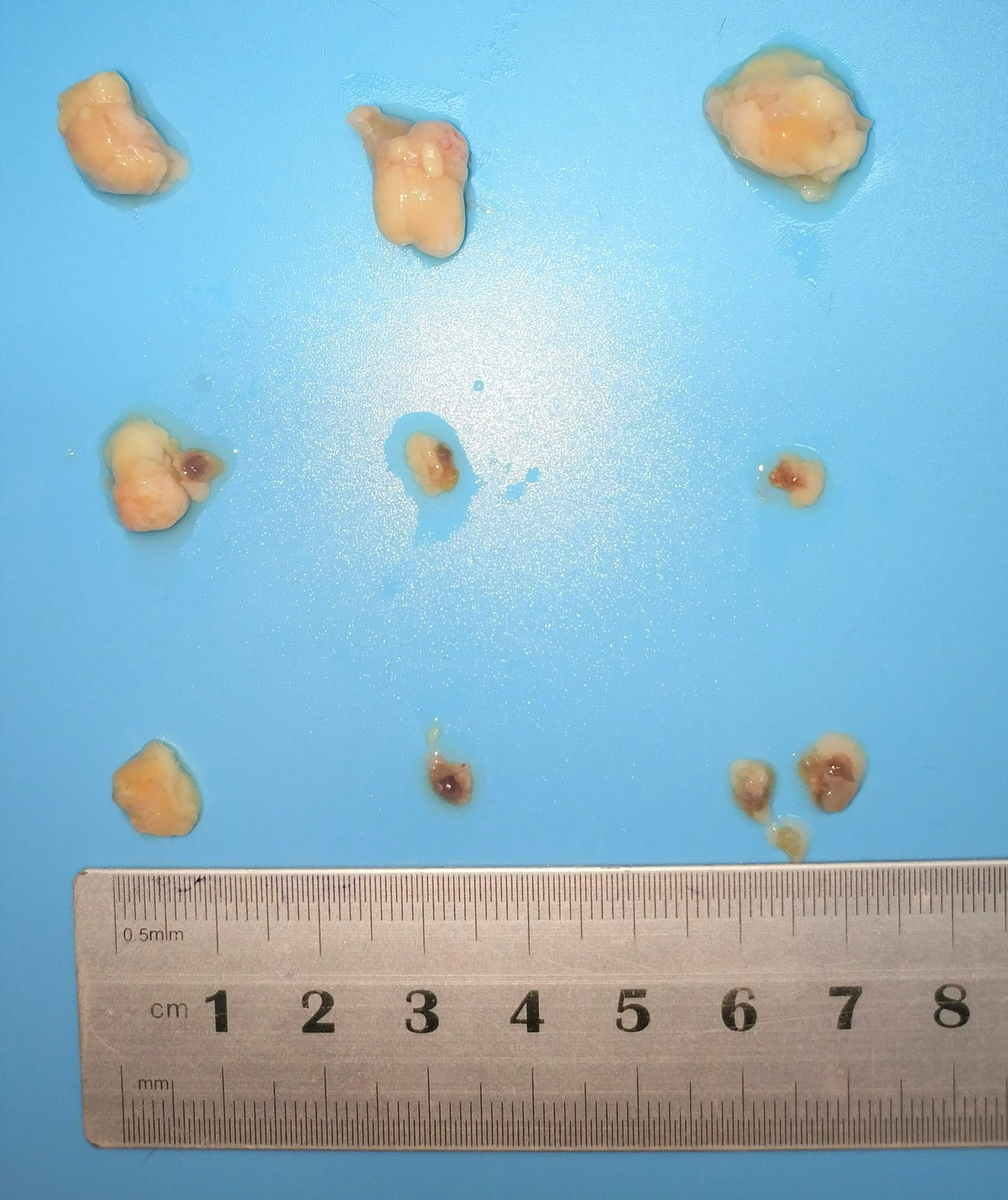

Supplement: Supplementary file 1 [file DataSheet_1.zip › raw original data-Fig1/Fig1b.jpg]

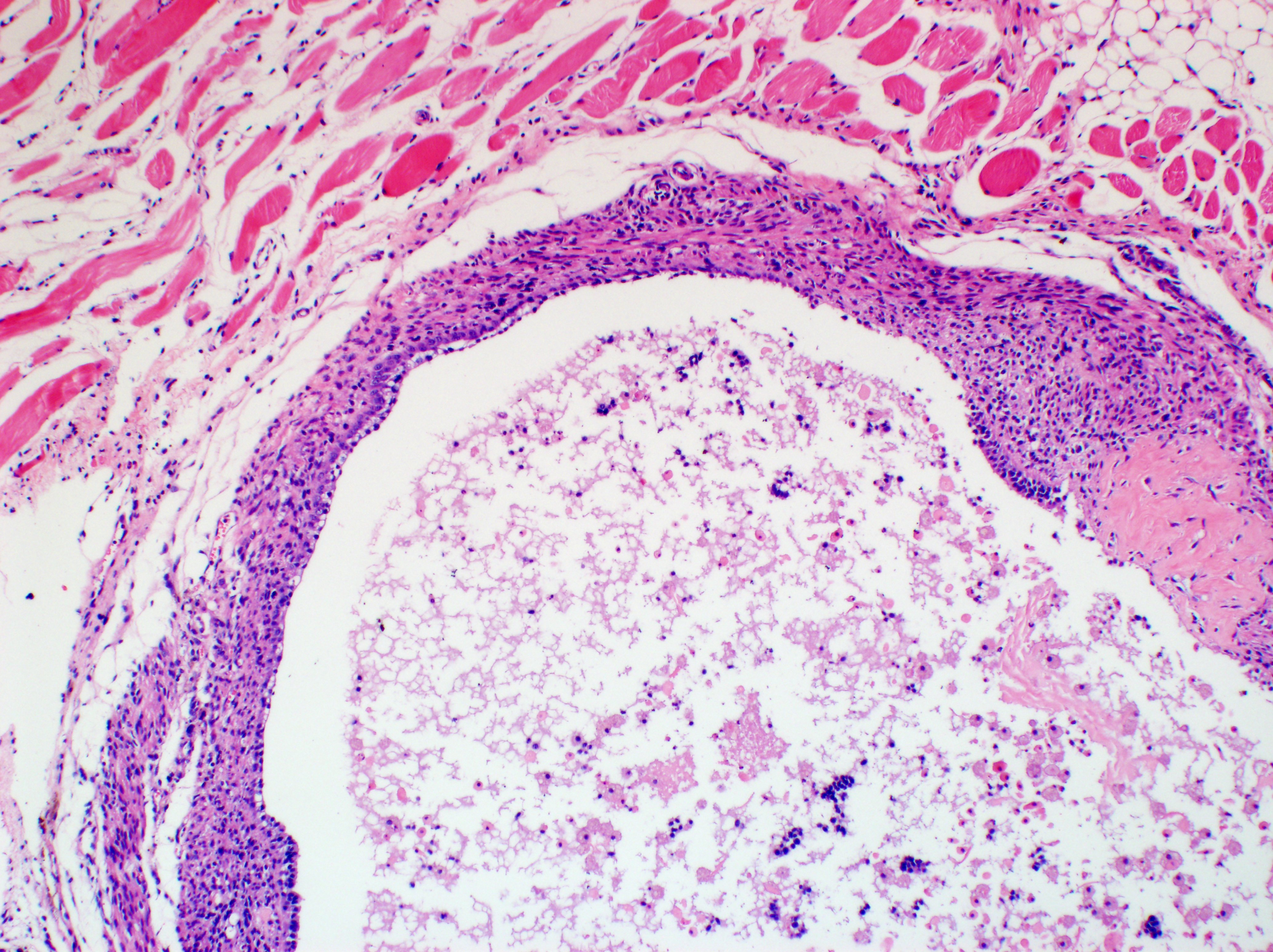

Supplement: Supplementary file 1 [file DataSheet_1.zip › raw original data-Fig1/Fig1D-EMS+SCM198.jpg]

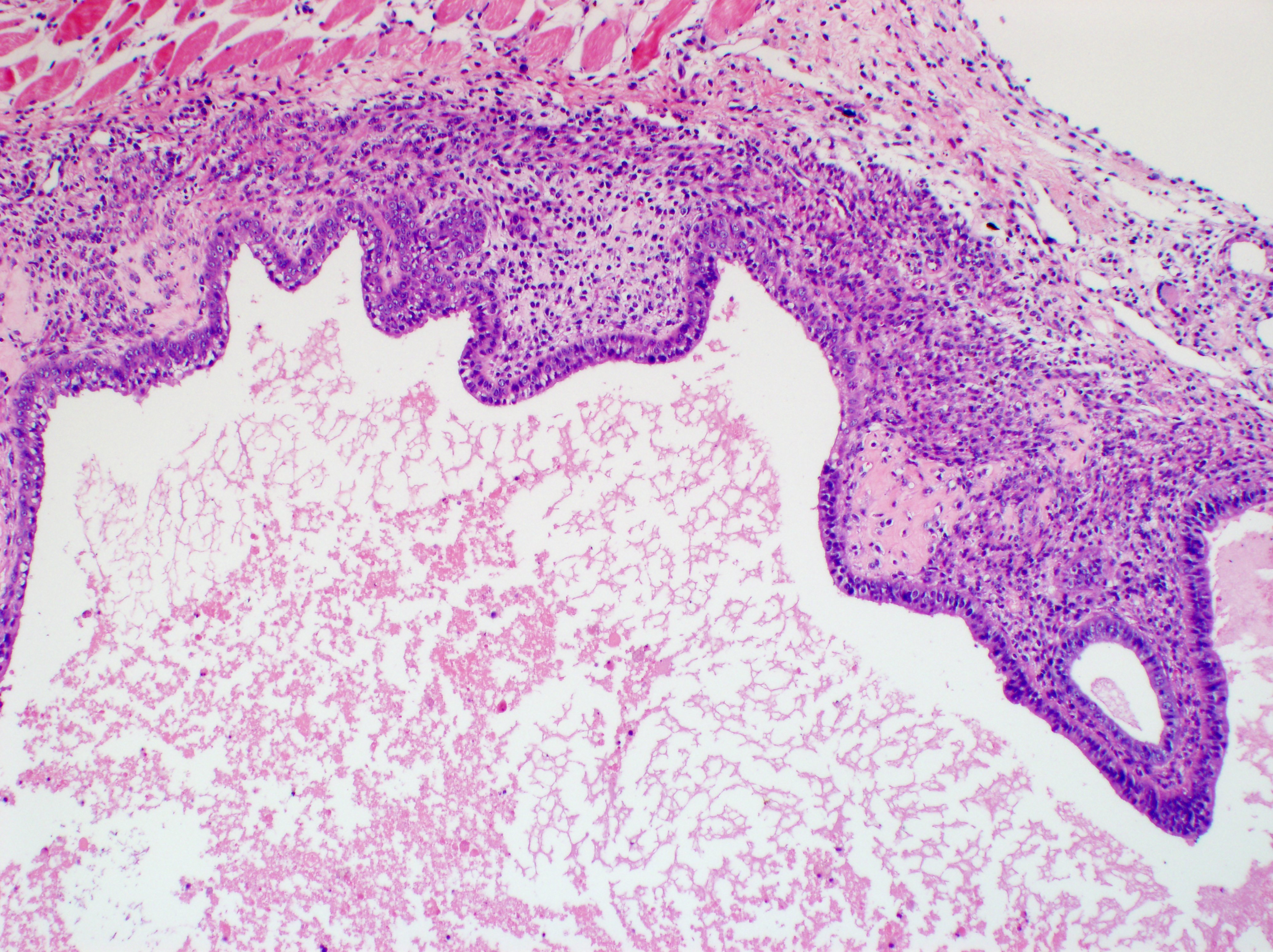

Supplement: Supplementary file 1 [file DataSheet_1.zip › raw original data-Fig1/Fig1D-EMS.jpg]

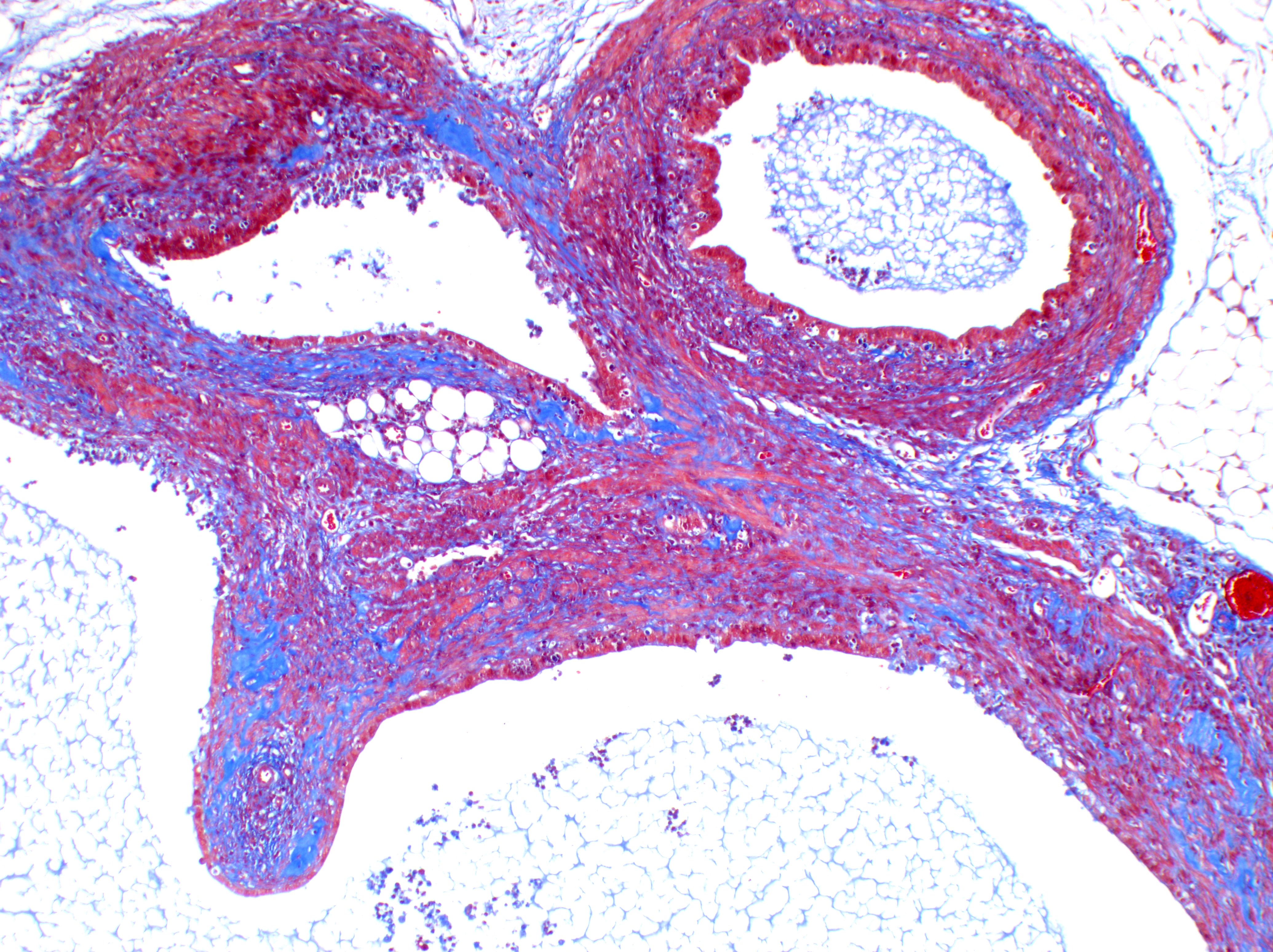

Supplement: Supplementary file 1 [file DataSheet_1.zip › raw original data-Fig1/Fig1E-EMS+SCM198.jpg]

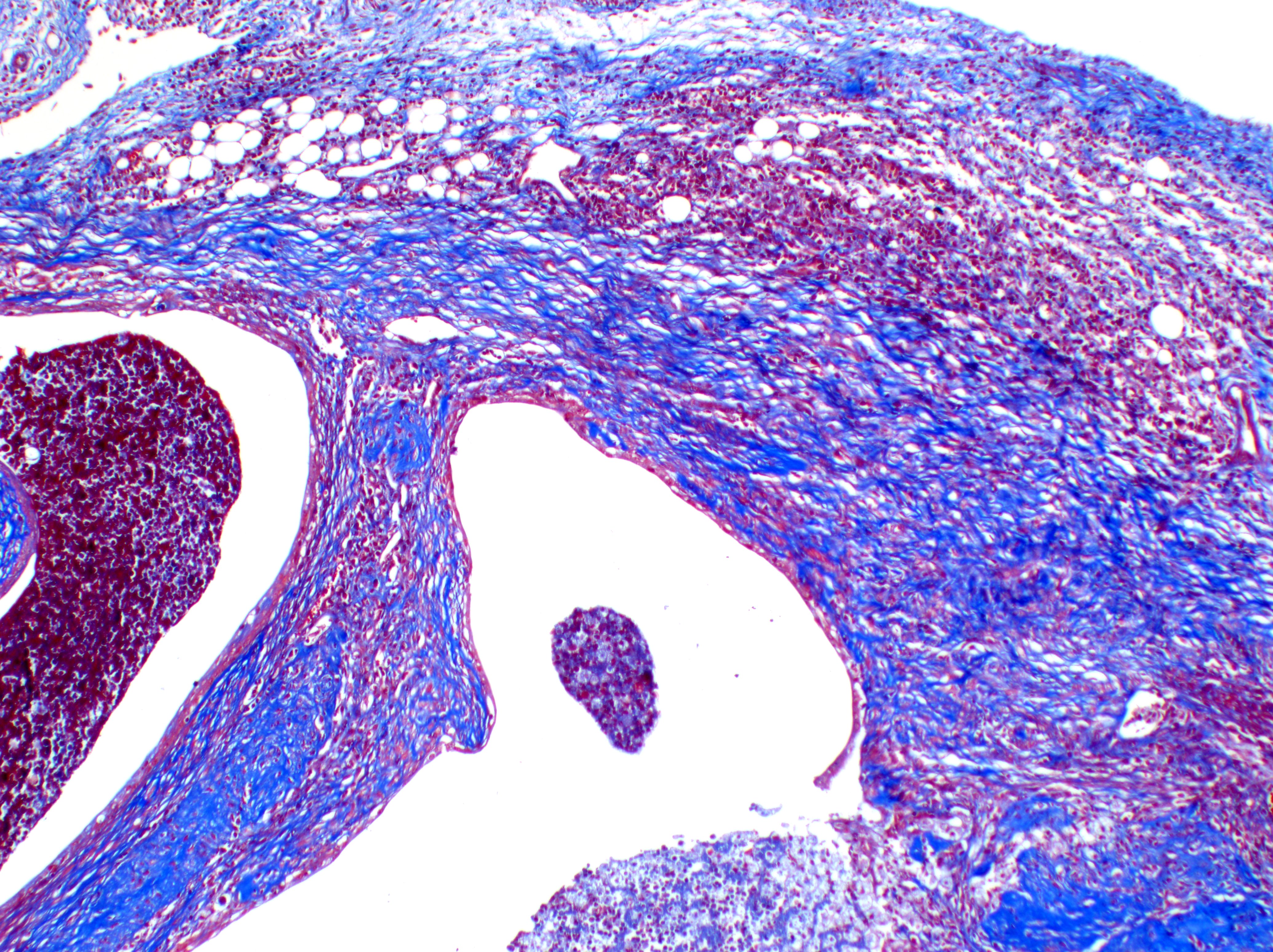

Supplement: Supplementary file 1 [file DataSheet_1.zip › raw original data-Fig1/Fig1E-EMS.jpg]

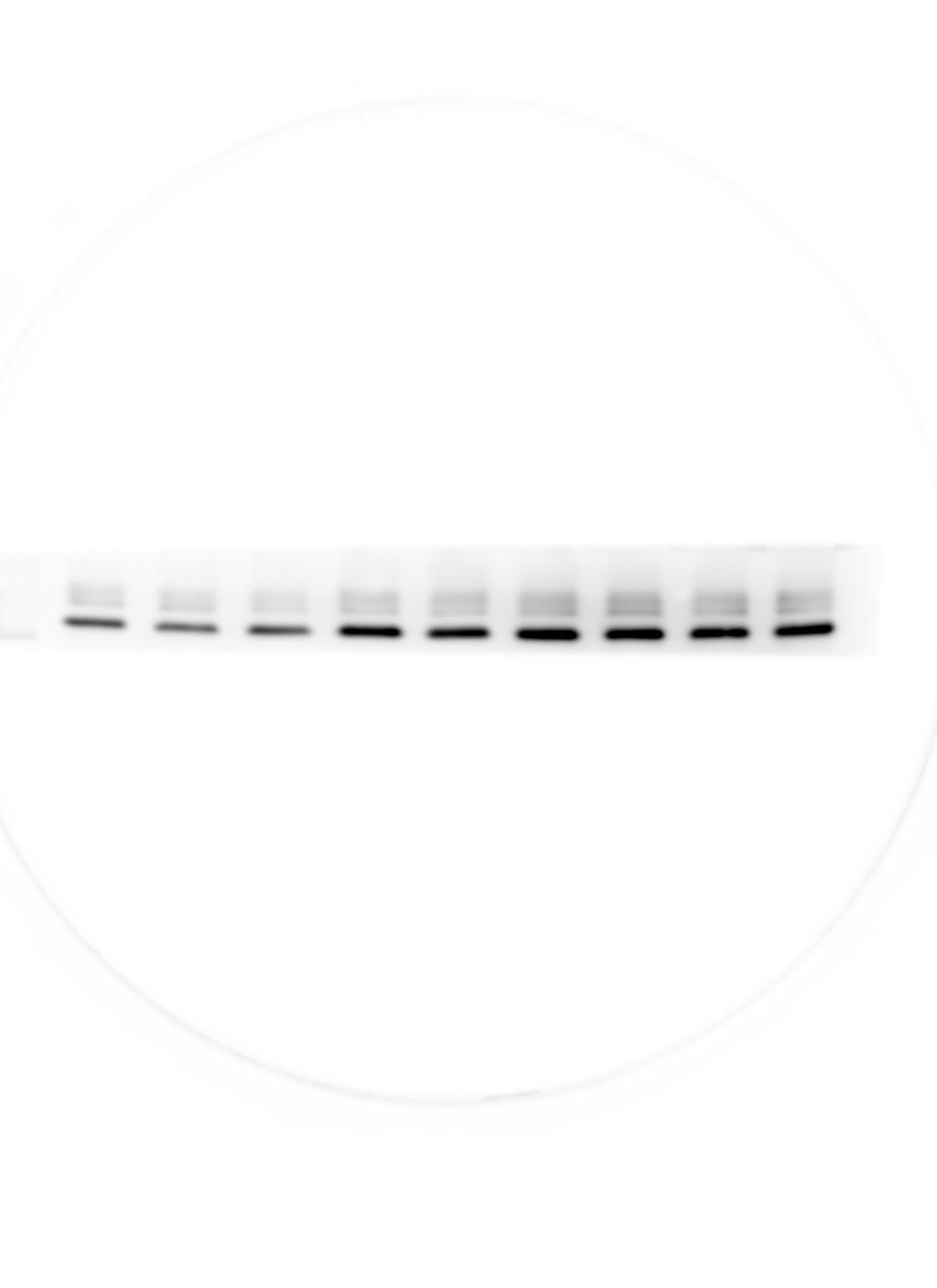

Supplement: Supplementary file 1 [file DataSheet_1.zip › raw original data-Fig1/WB/Fig1F-Bax.jpg]

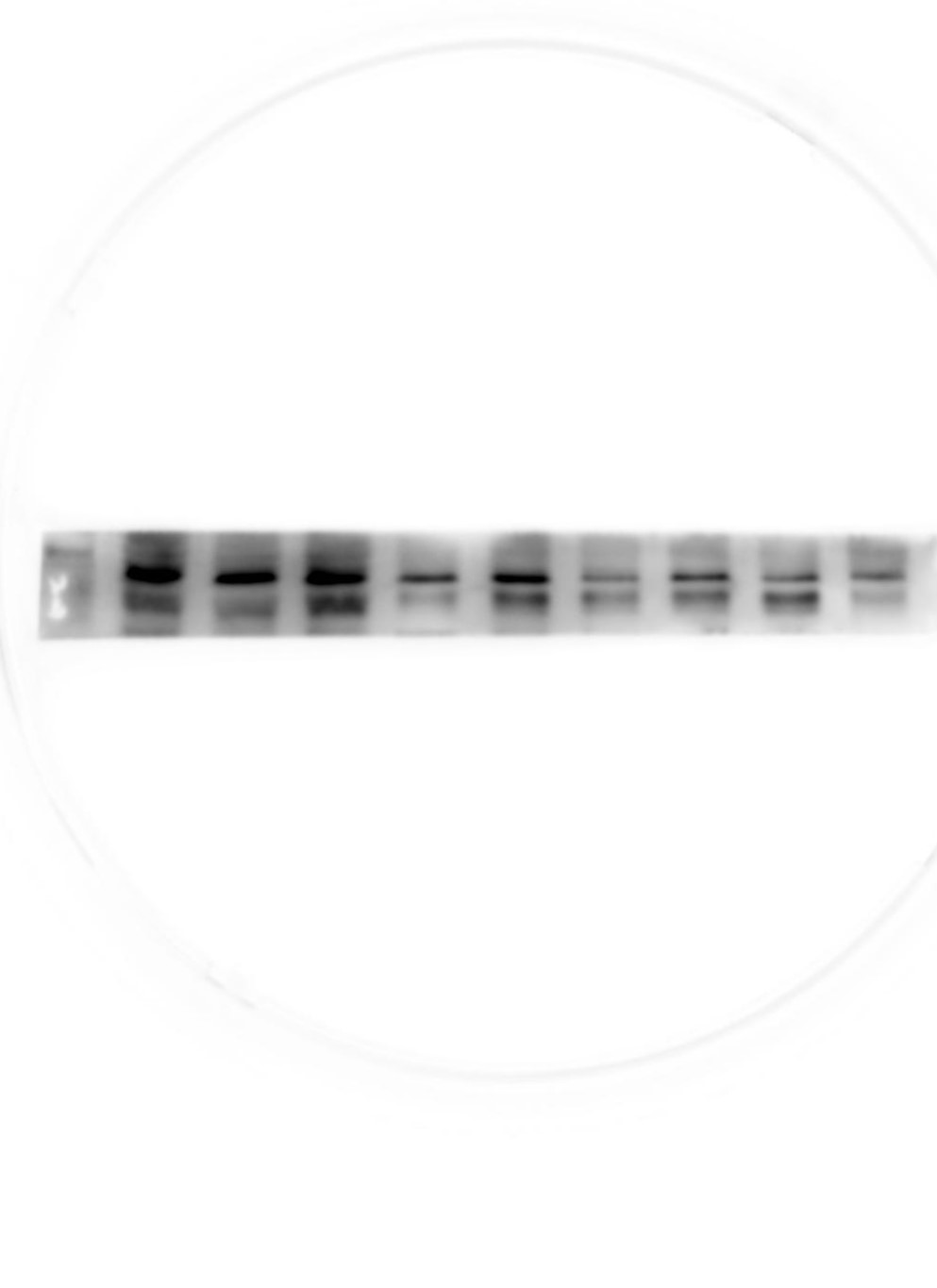

Supplement: Supplementary file 1 [file DataSheet_1.zip › raw original data-Fig1/WB/Fig1F-Bcl2.jpg]

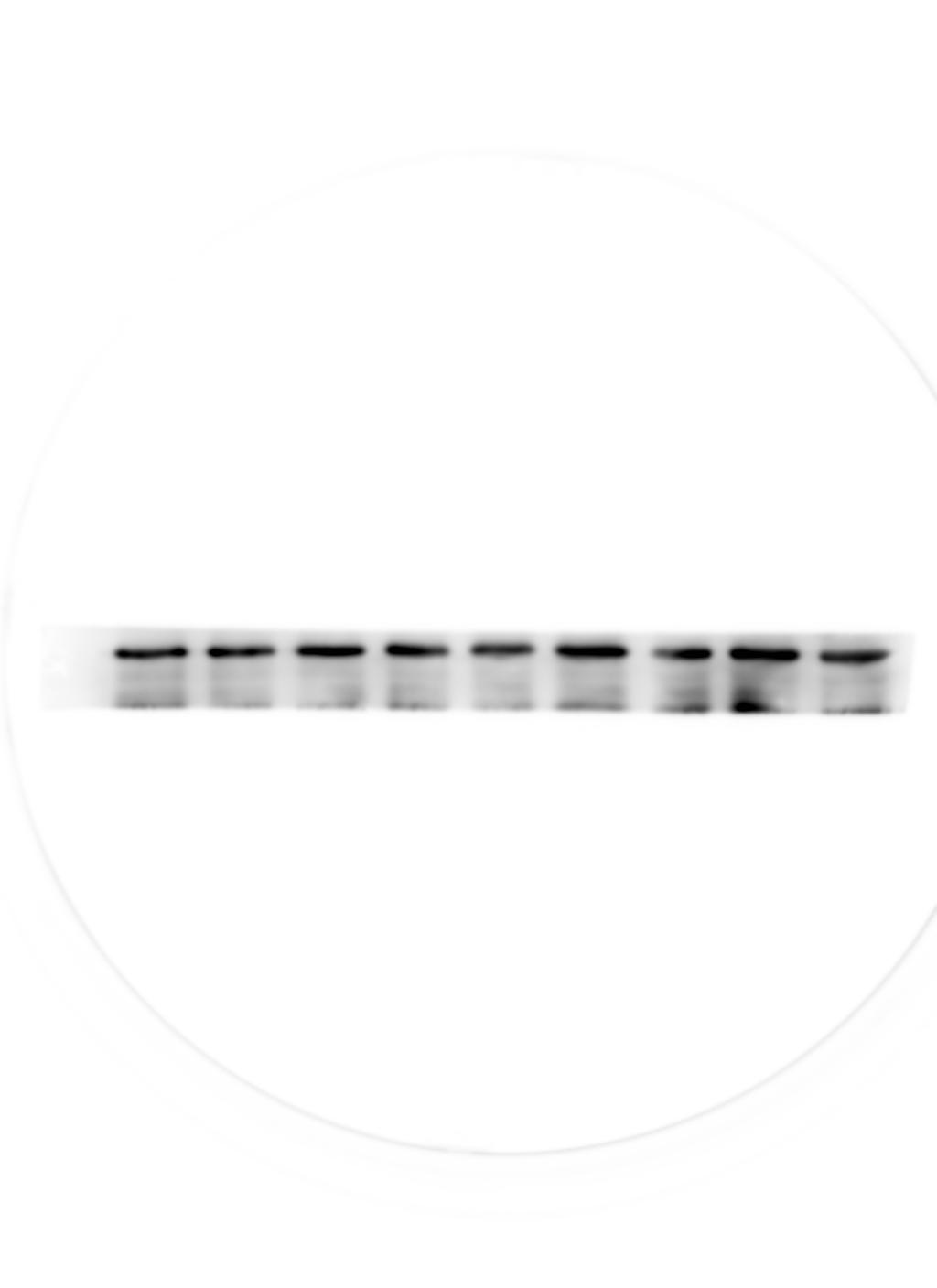

Supplement: Supplementary file 1 [file DataSheet_1.zip › raw original data-Fig1/WB/Fig1F-GAPDH.jpg]

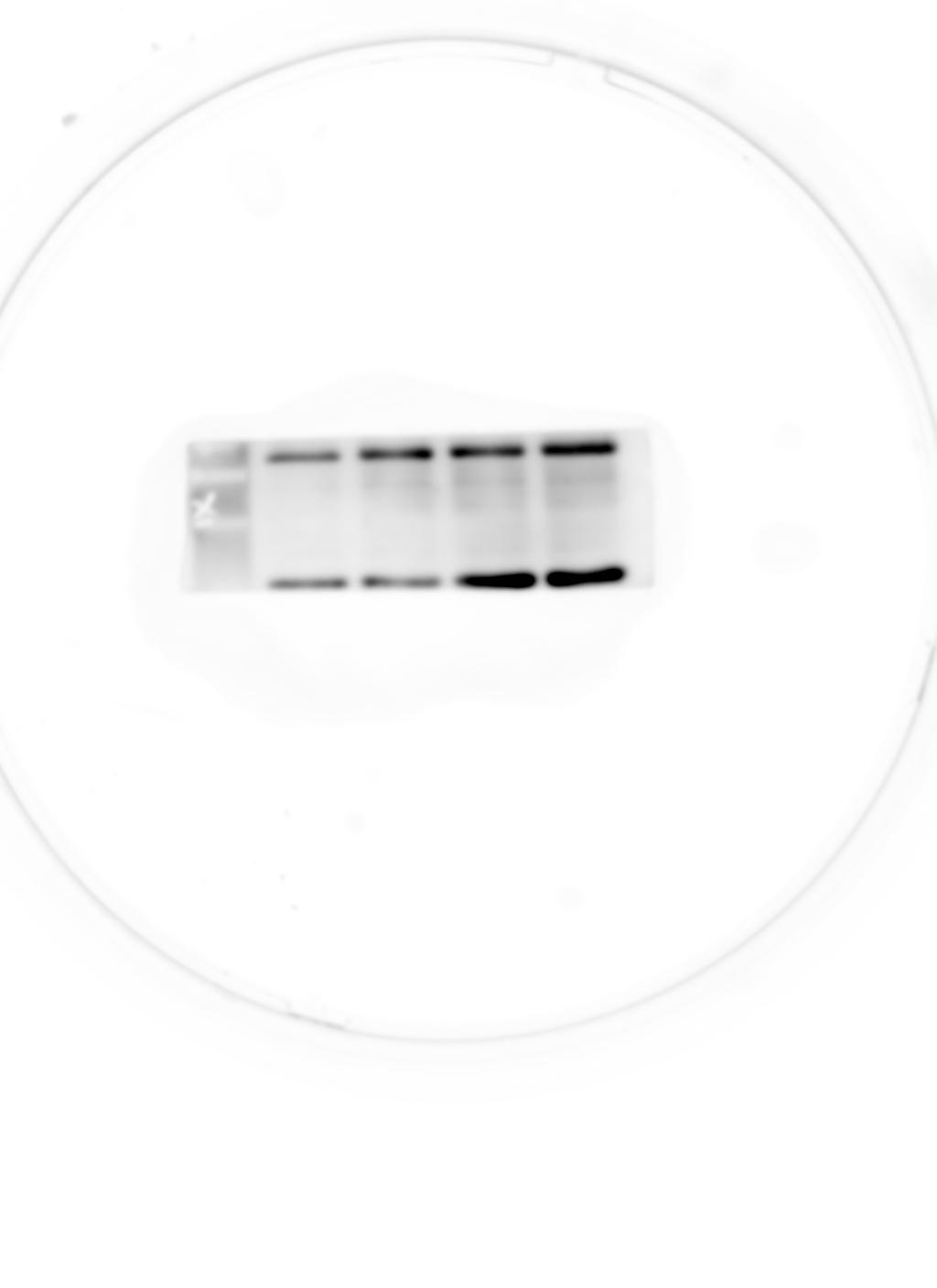

Supplement: Supplementary file 1 [file DataSheet_1.zip › raw original data-Fig1/WB/Fig1G-Bax.jpg]

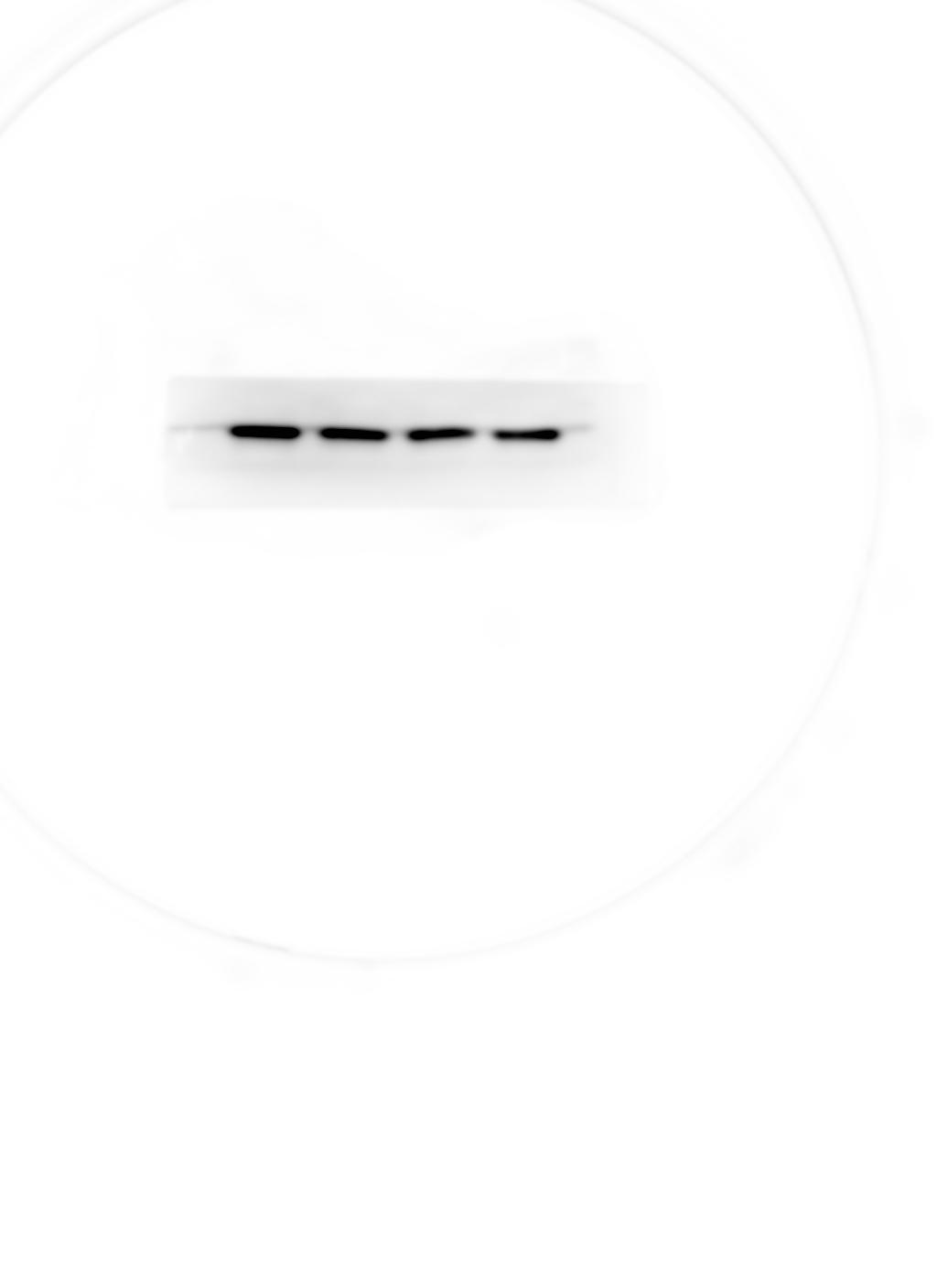

Supplement: Supplementary file 1 [file DataSheet_1.zip › raw original data-Fig1/WB/Fig1G-Bcl2.jpg]

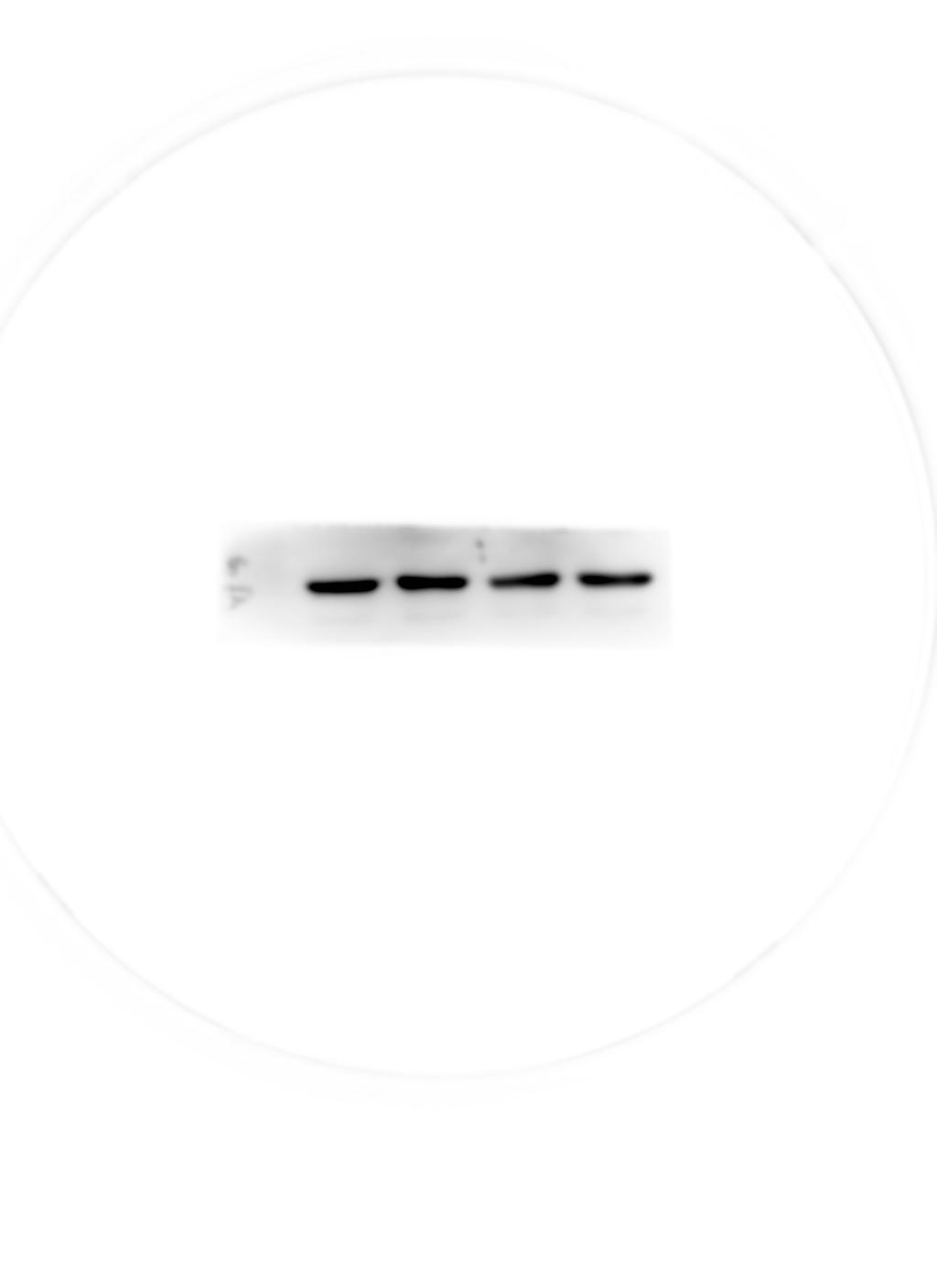

Supplement: Supplementary file 1 [file DataSheet_1.zip › raw original data-Fig1/WB/Fig1G-GAPDH.jpg]

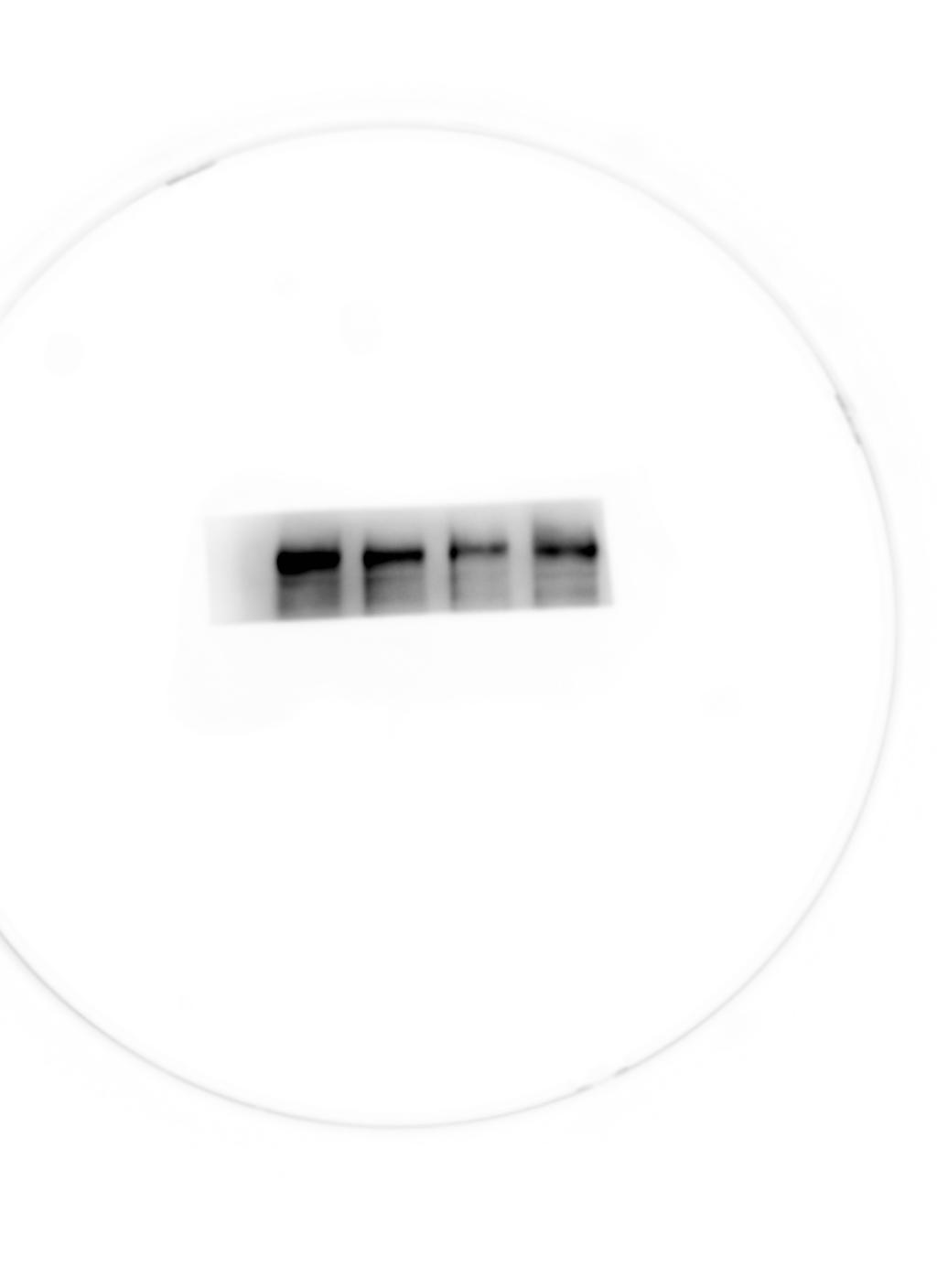

Supplement: Supplementary file 1 [file DataSheet_1.zip › raw original data-Fig1/WB/Fig1H-FN1.jpg]

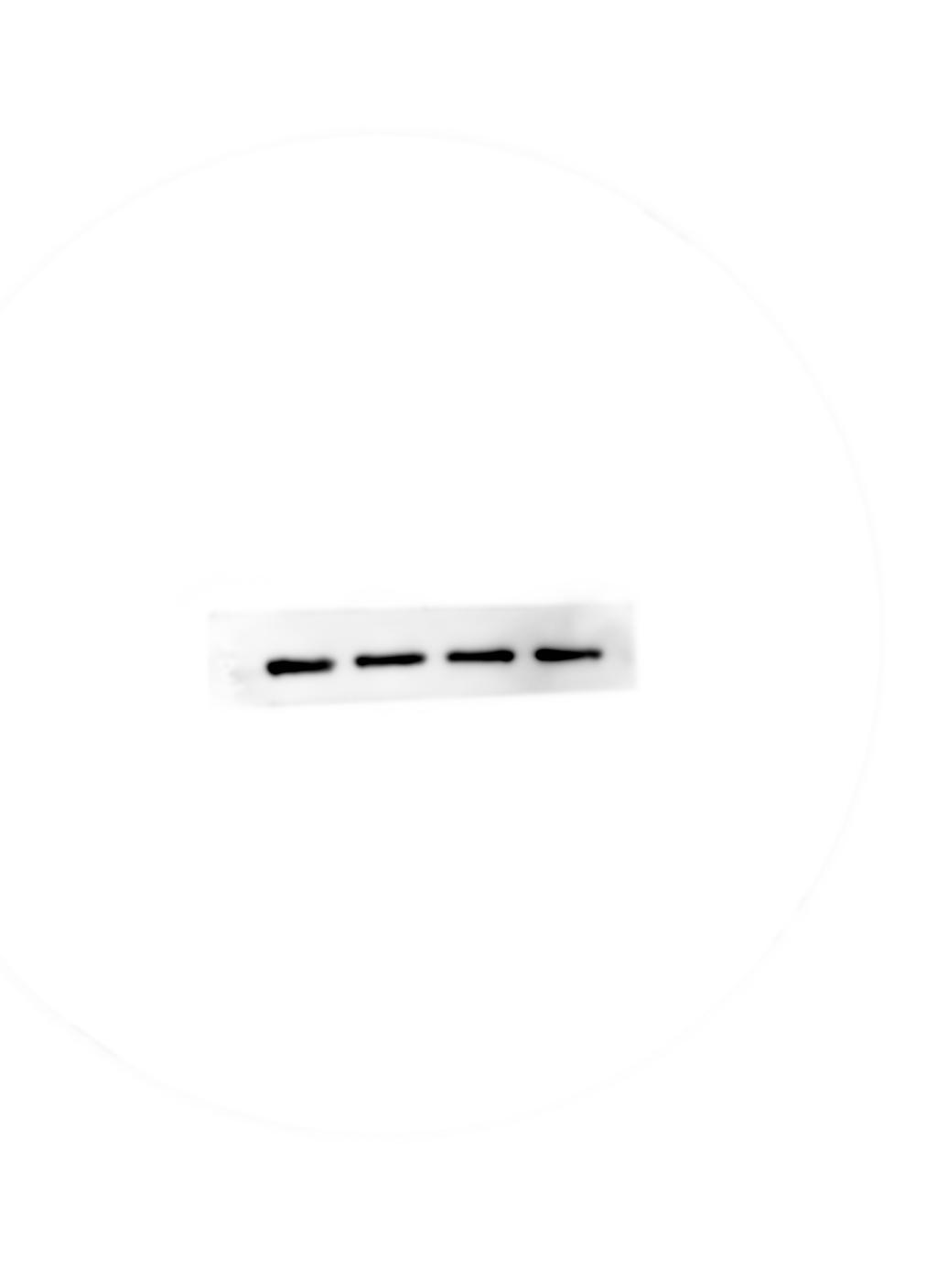

Supplement: Supplementary file 1 [file DataSheet_1.zip › raw original data-Fig1/WB/Fig1H-TUBULIN.jpg]

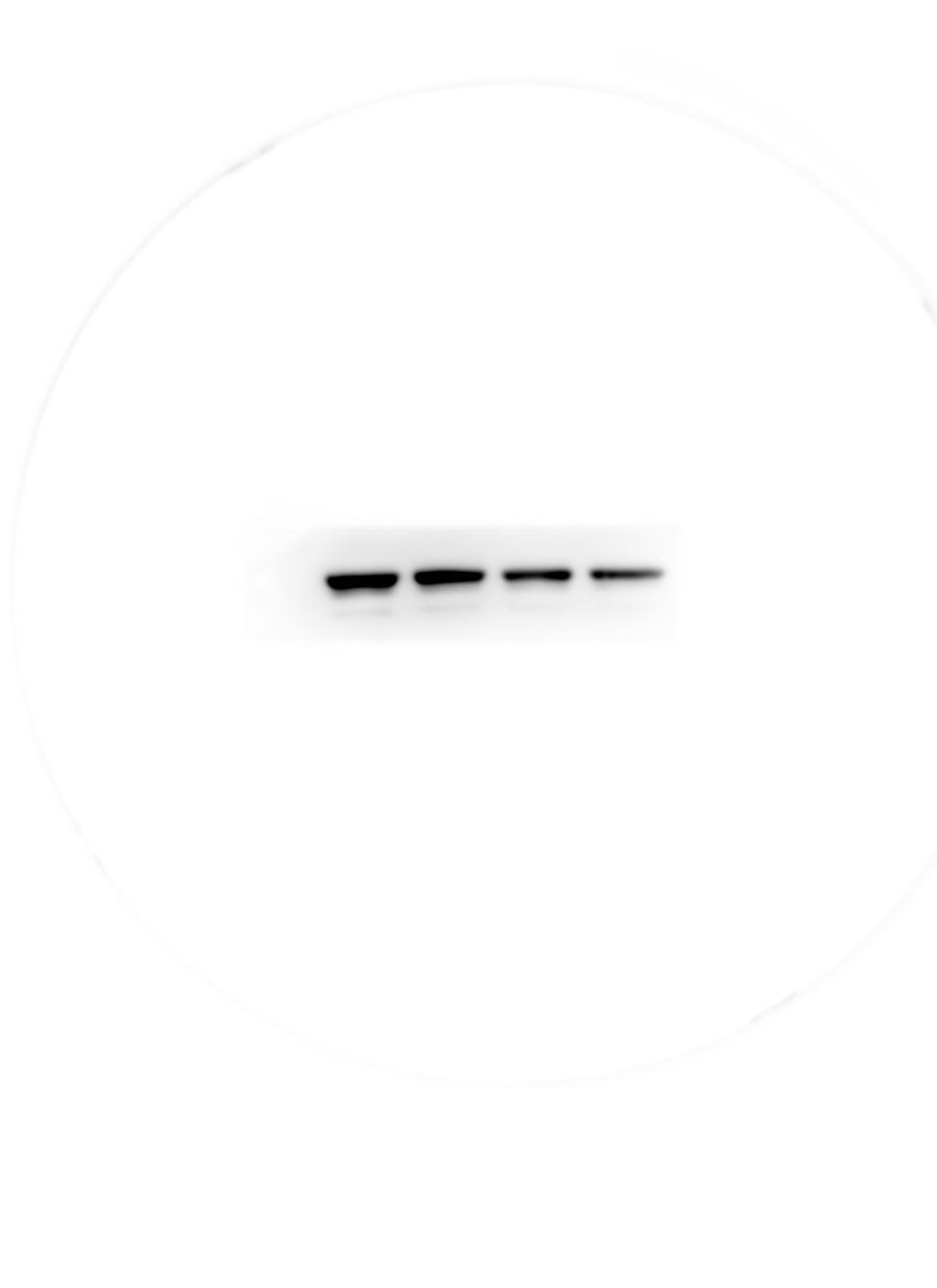

Supplement: Supplementary file 1 [file DataSheet_1.zip › raw original data-Fig1/WB/Fig1H-VIMENTIN.jpg]

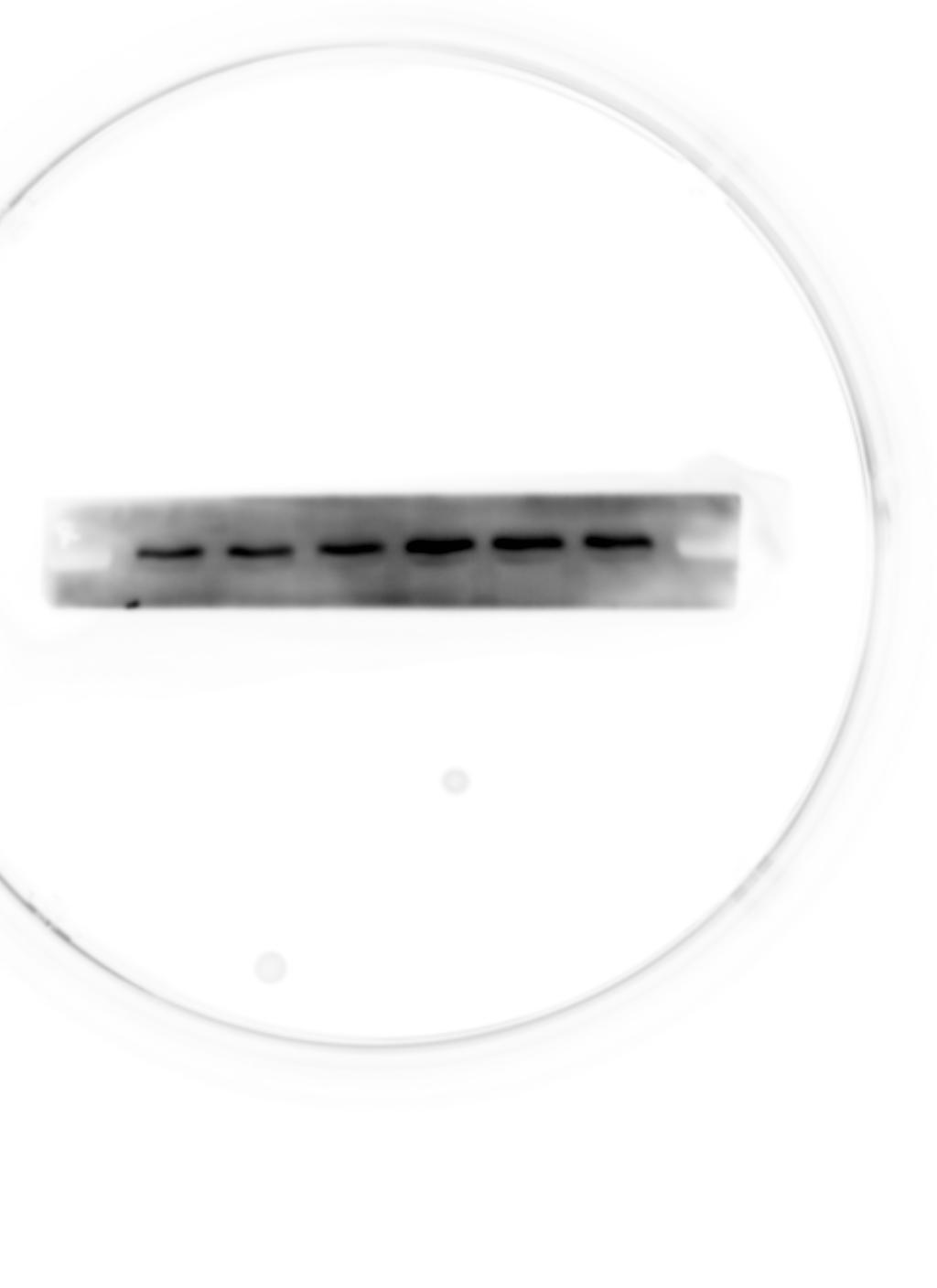

Supplement: Supplementary file 2 [file DataSheet_2.zip › raw original data-Fig3/WB/Fig3B-ERa.jpg]

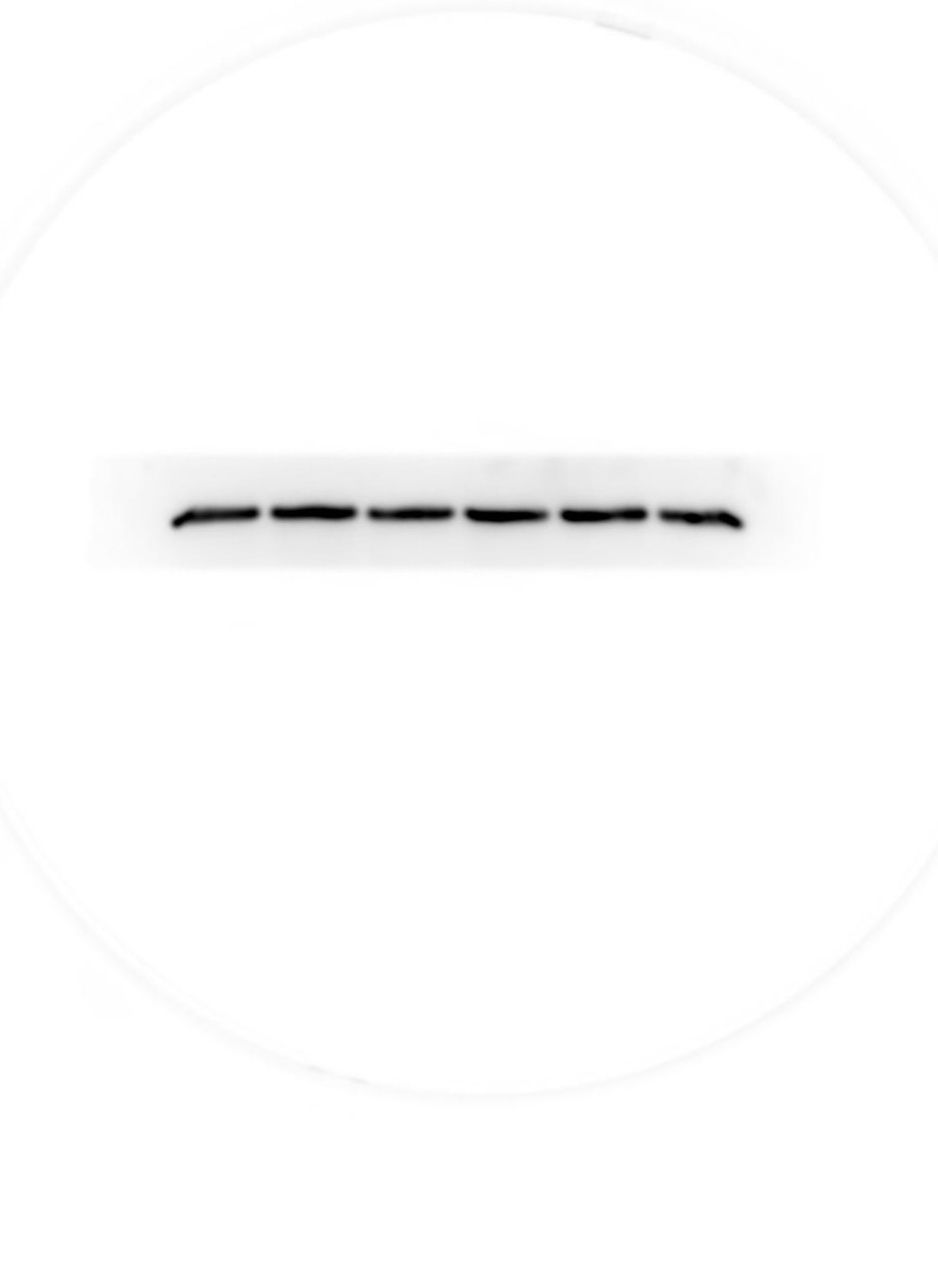

Supplement: Supplementary file 2 [file DataSheet_2.zip › raw original data-Fig3/WB/Fig3B-GAPDH.jpg]

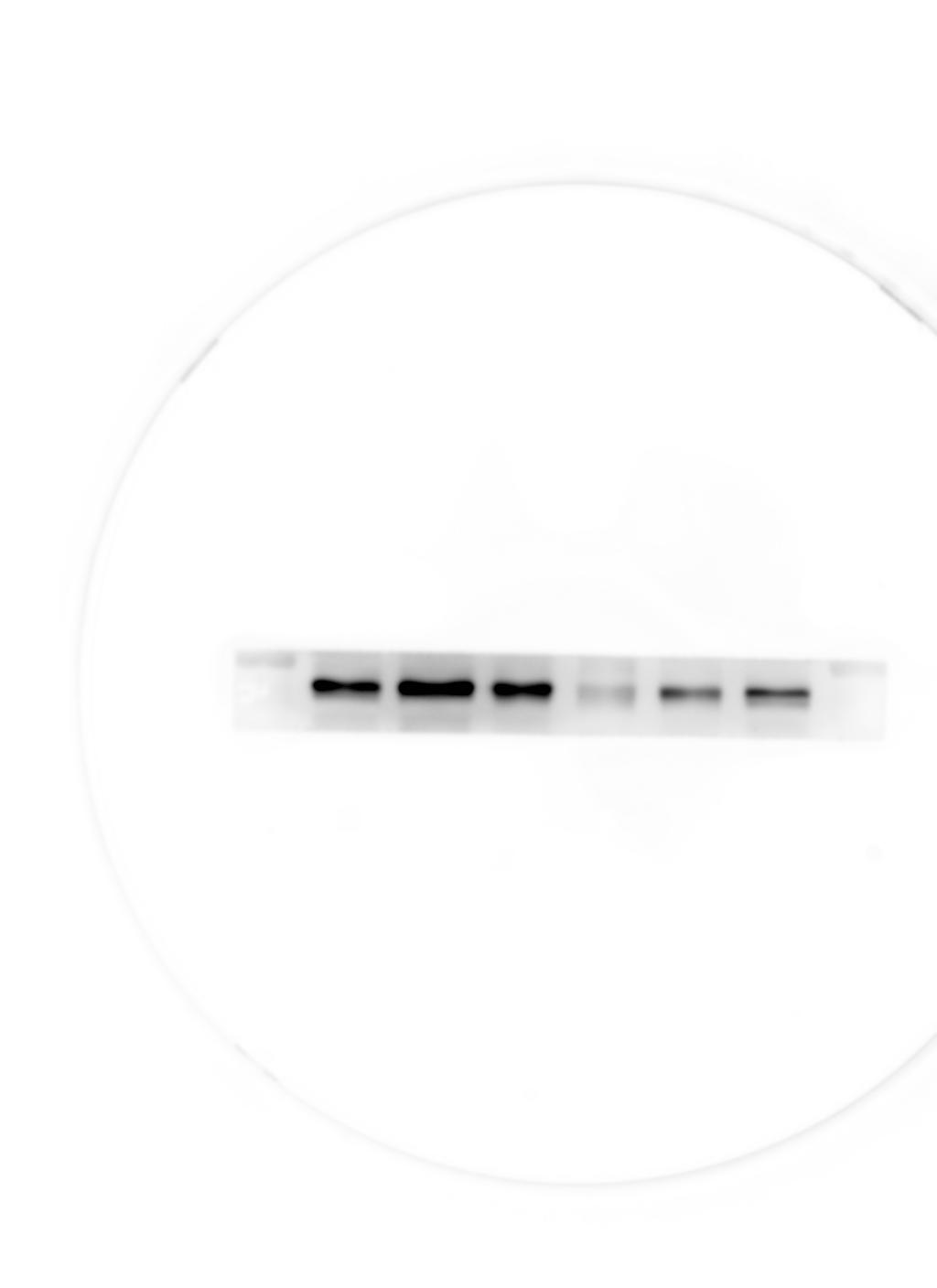

Supplement: Supplementary file 2 [file DataSheet_2.zip › raw original data-Fig3/WB/Fig3C-BECN1.jpg]

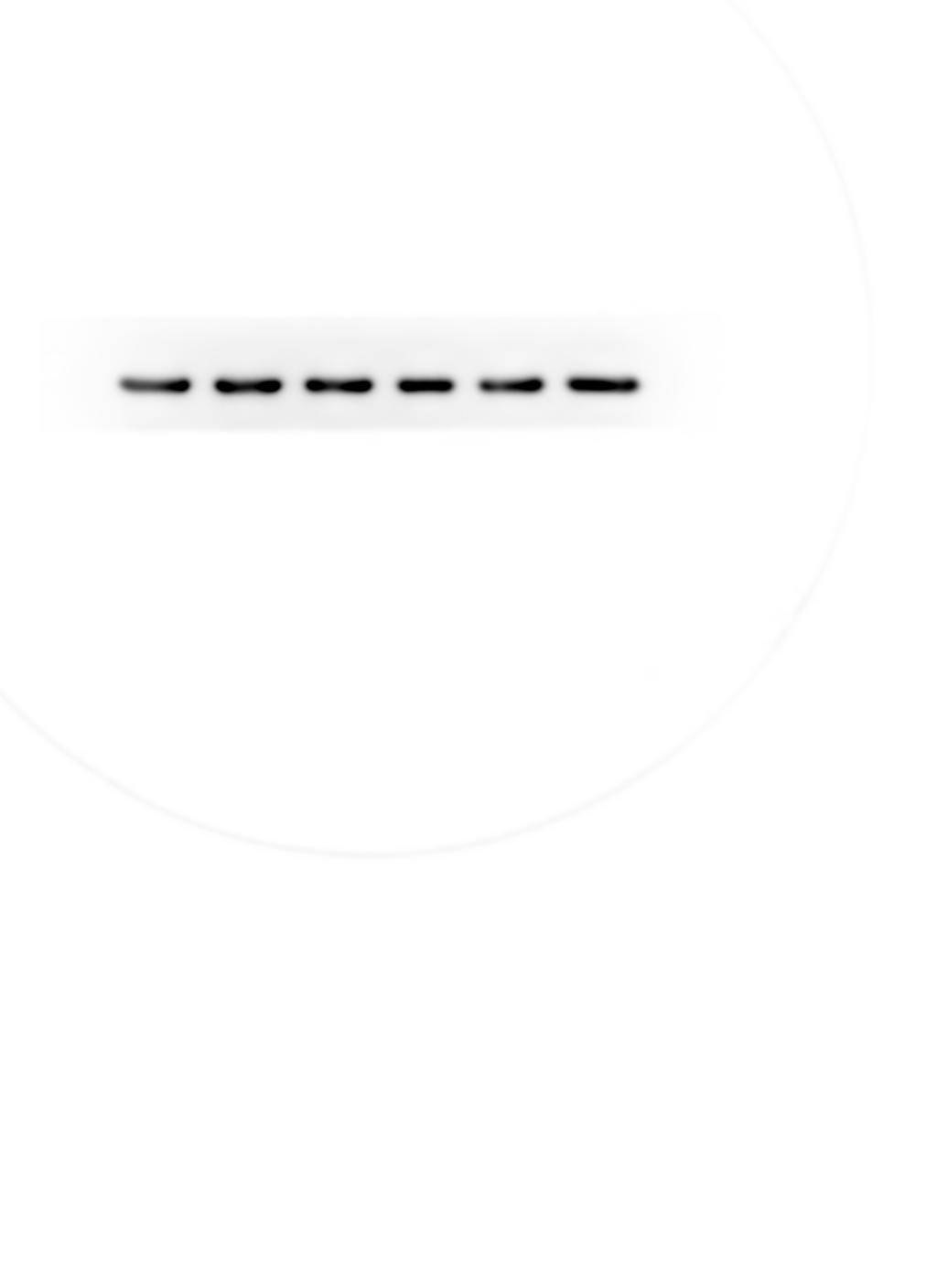

Supplement: Supplementary file 2 [file DataSheet_2.zip › raw original data-Fig3/WB/Fig3C-GAPDH.jpg]

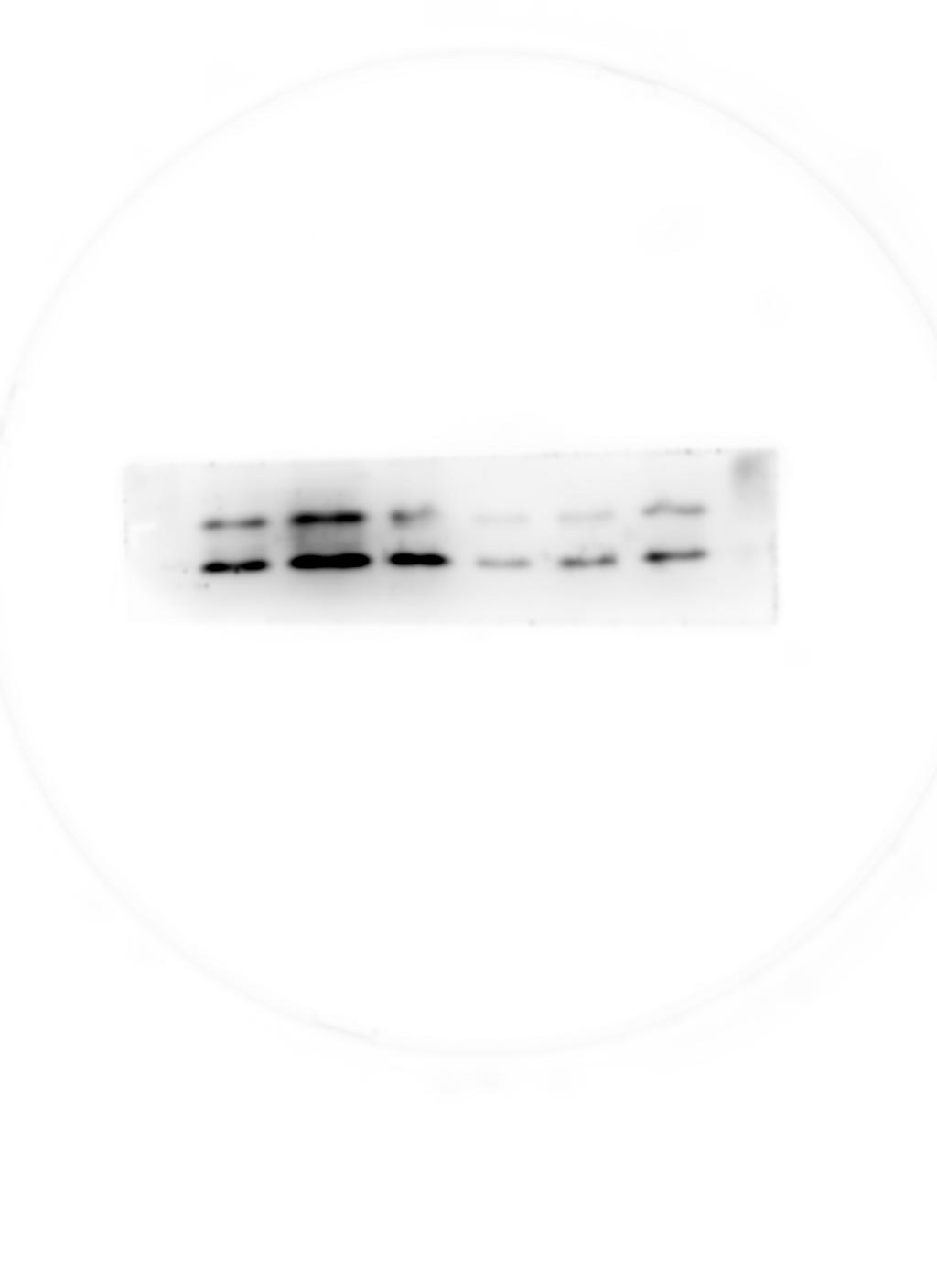

Supplement: Supplementary file 2 [file DataSheet_2.zip › raw original data-Fig3/WB/Fig3C-LC3B.jpg]

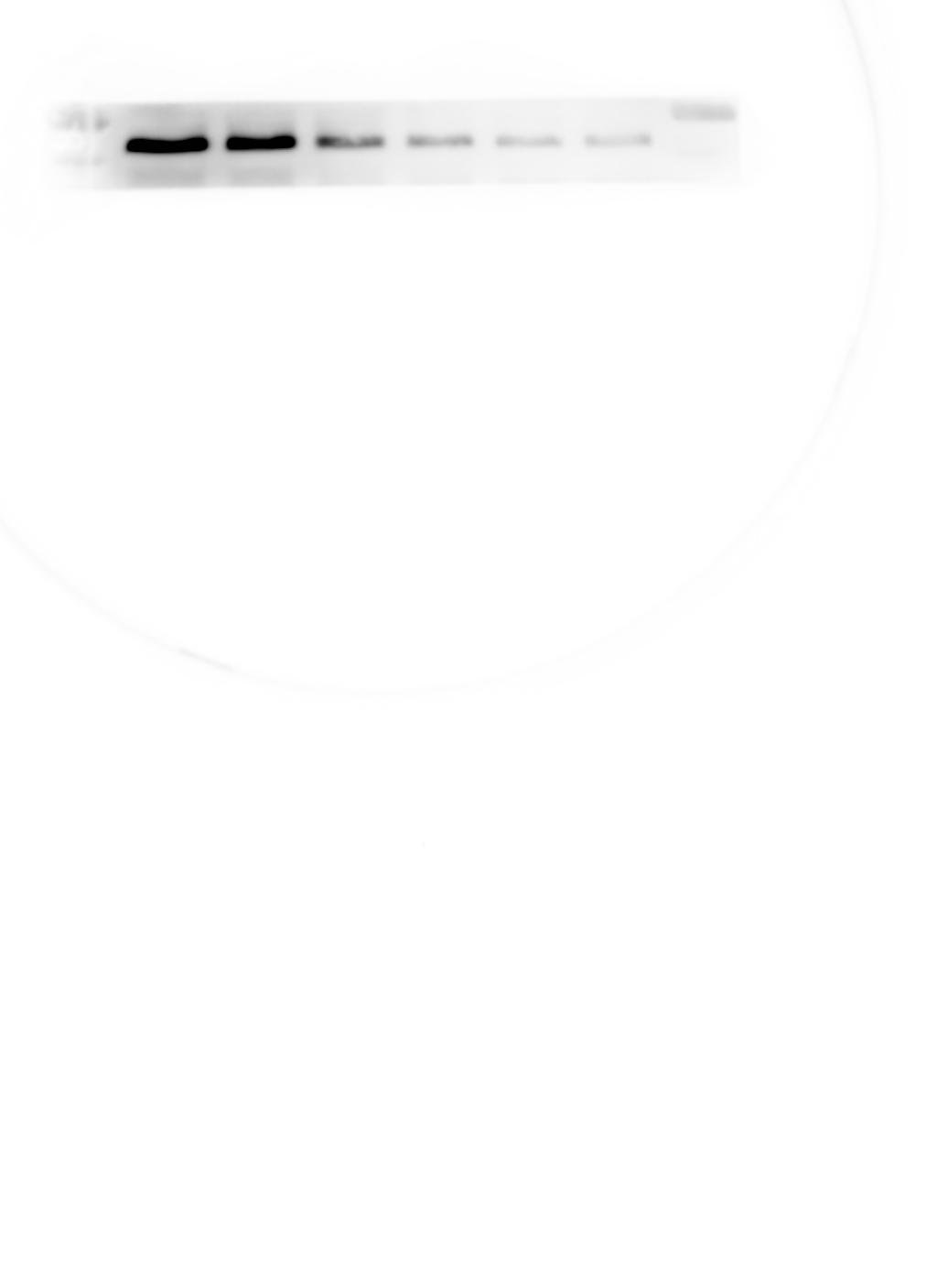

Supplement: Supplementary file 2 [file DataSheet_2.zip › raw original data-Fig3/WB/Fig3D-BECN1.jpg]

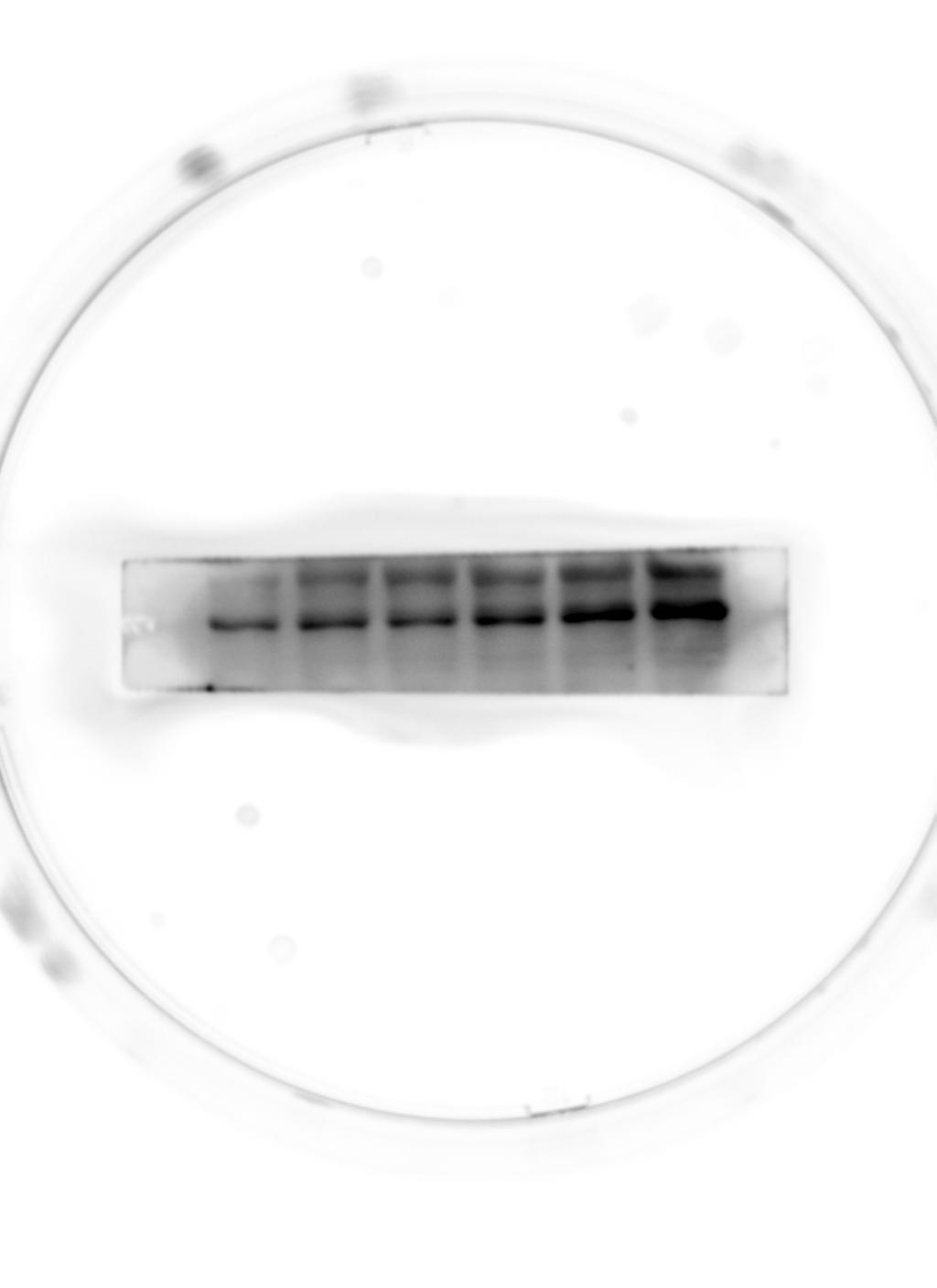

Supplement: Supplementary file 2 [file DataSheet_2.zip › raw original data-Fig3/WB/Fig3D-ERa.jpg]

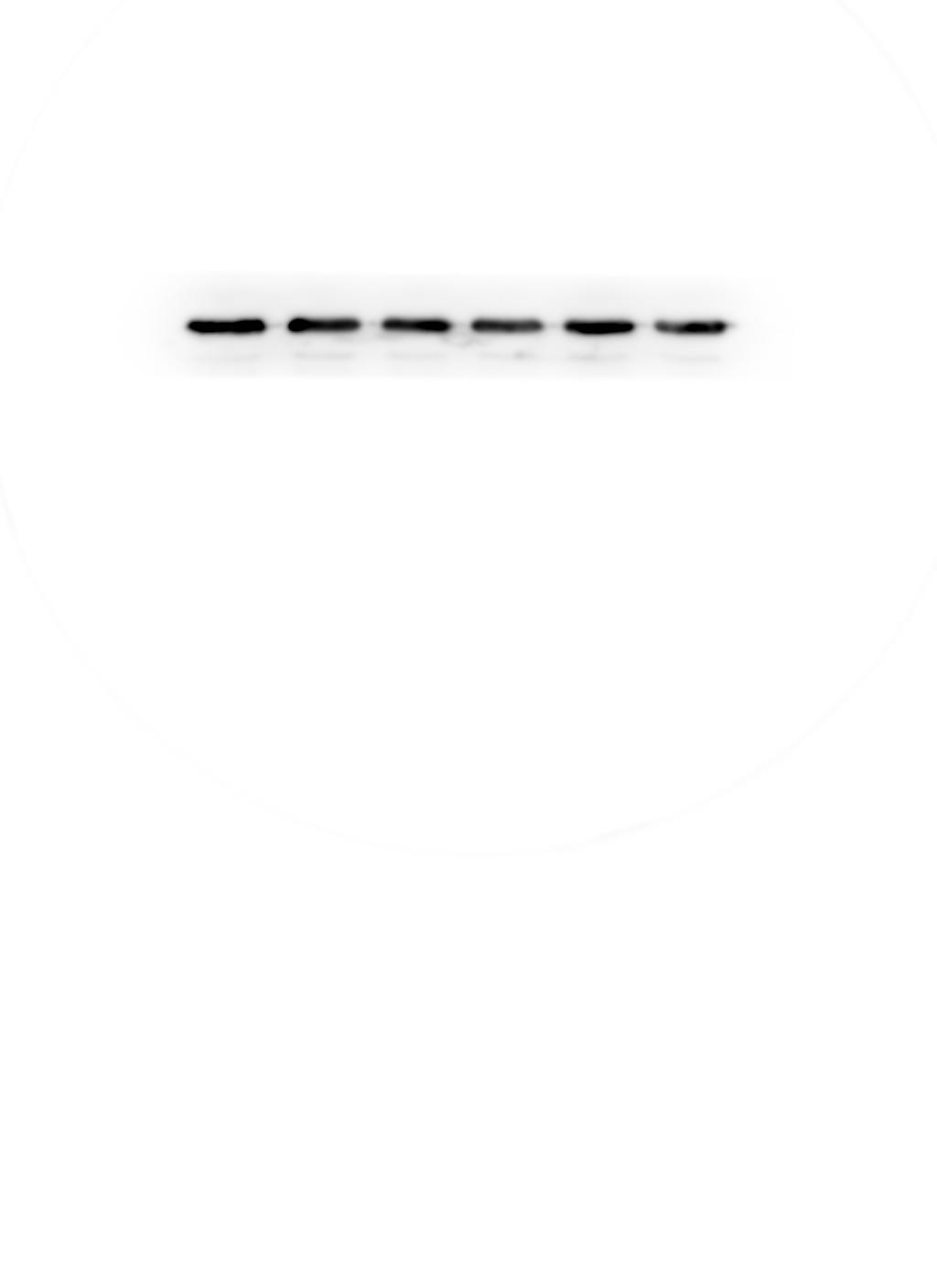

Supplement: Supplementary file 2 [file DataSheet_2.zip › raw original data-Fig3/WB/Fig3D-GAPDH.jpg]

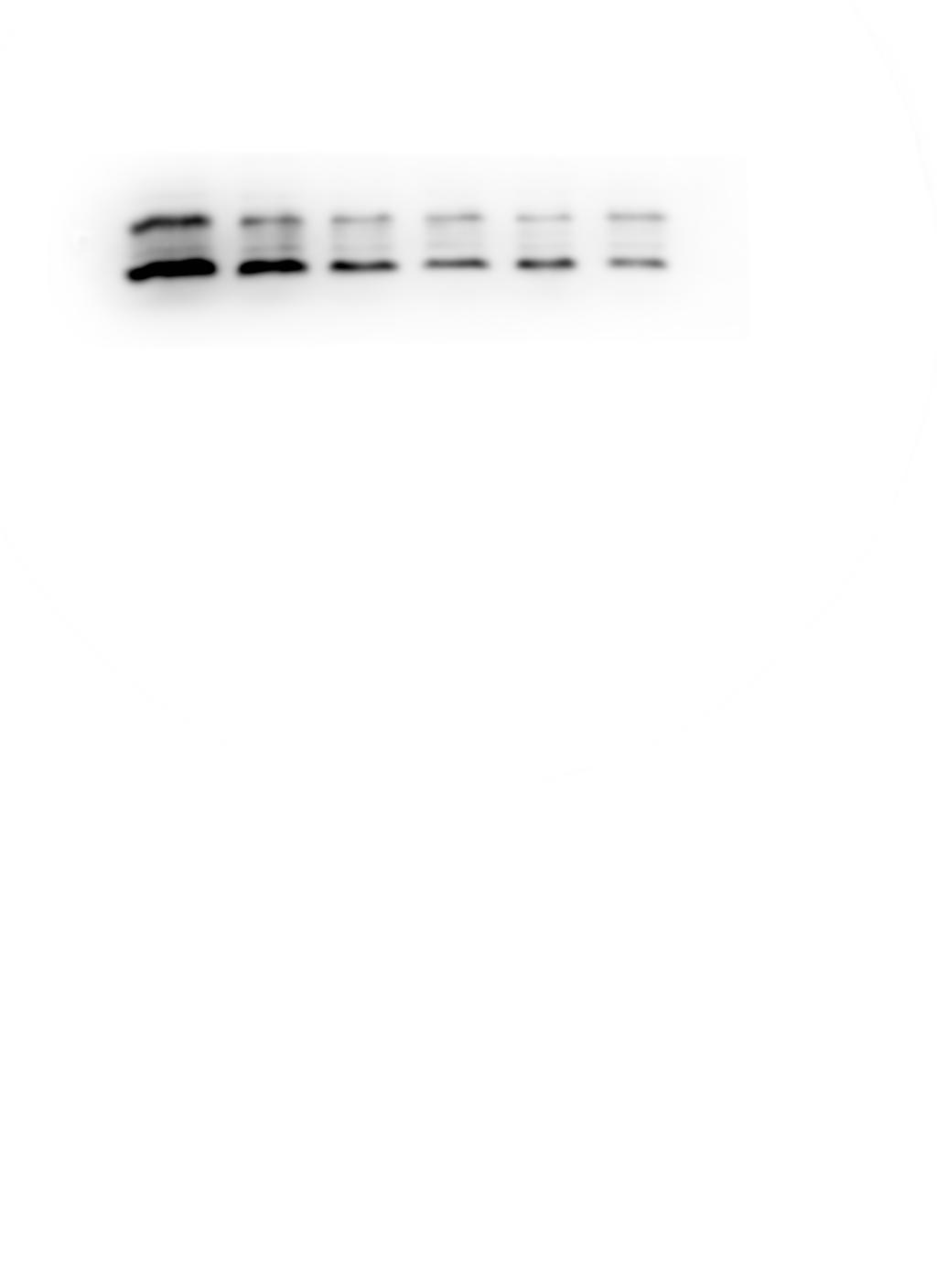

Supplement: Supplementary file 2 [file DataSheet_2.zip › raw original data-Fig3/WB/Fig3D-LC3B.jpg]

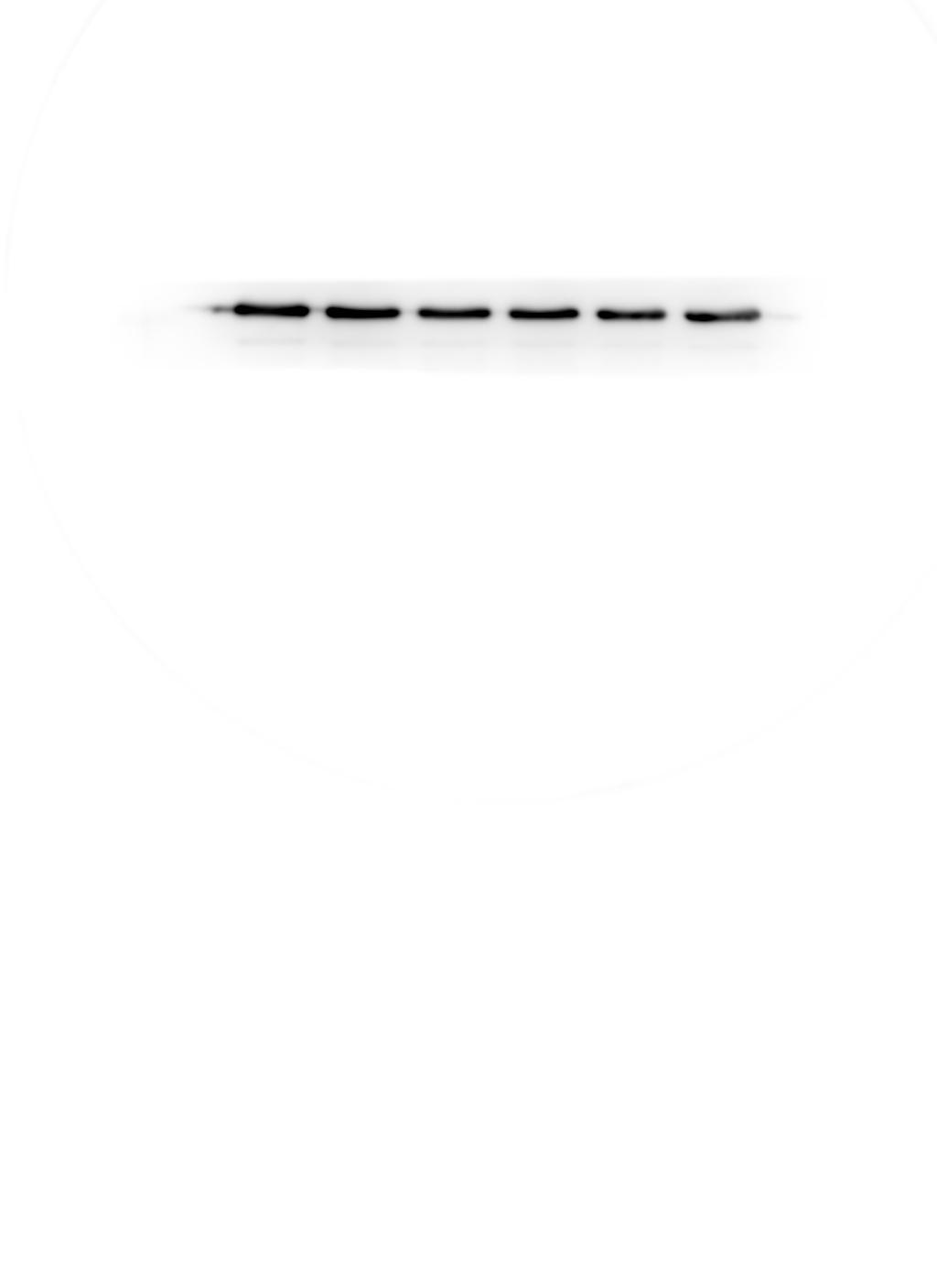

Supplement: Supplementary file 2 [file DataSheet_2.zip › raw original data-Fig3/WB/Fig3E-GAPDH.jpg]

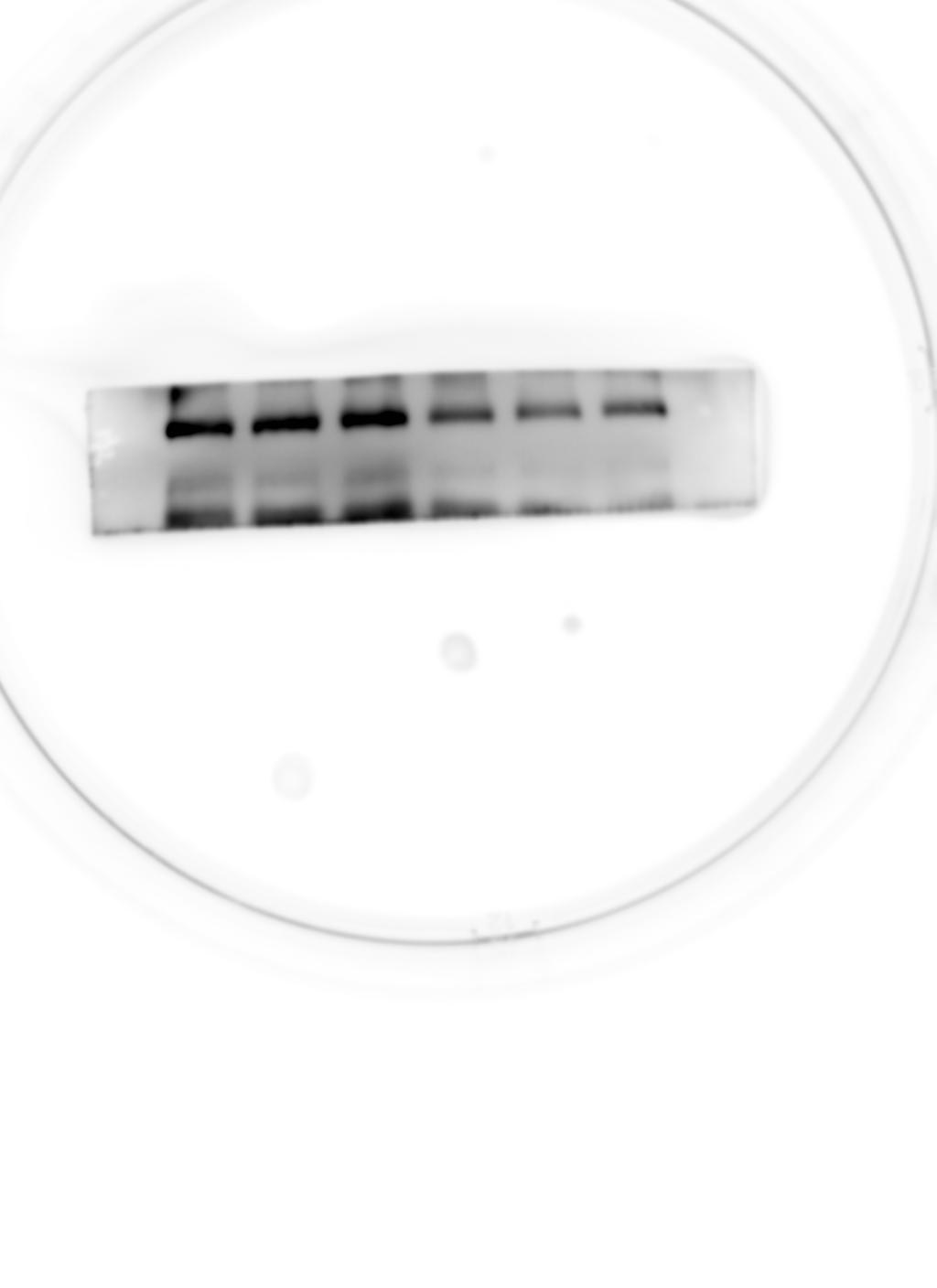

Supplement: Supplementary file 2 [file DataSheet_2.zip › raw original data-Fig3/WB/Fig3E-PR.jpg]

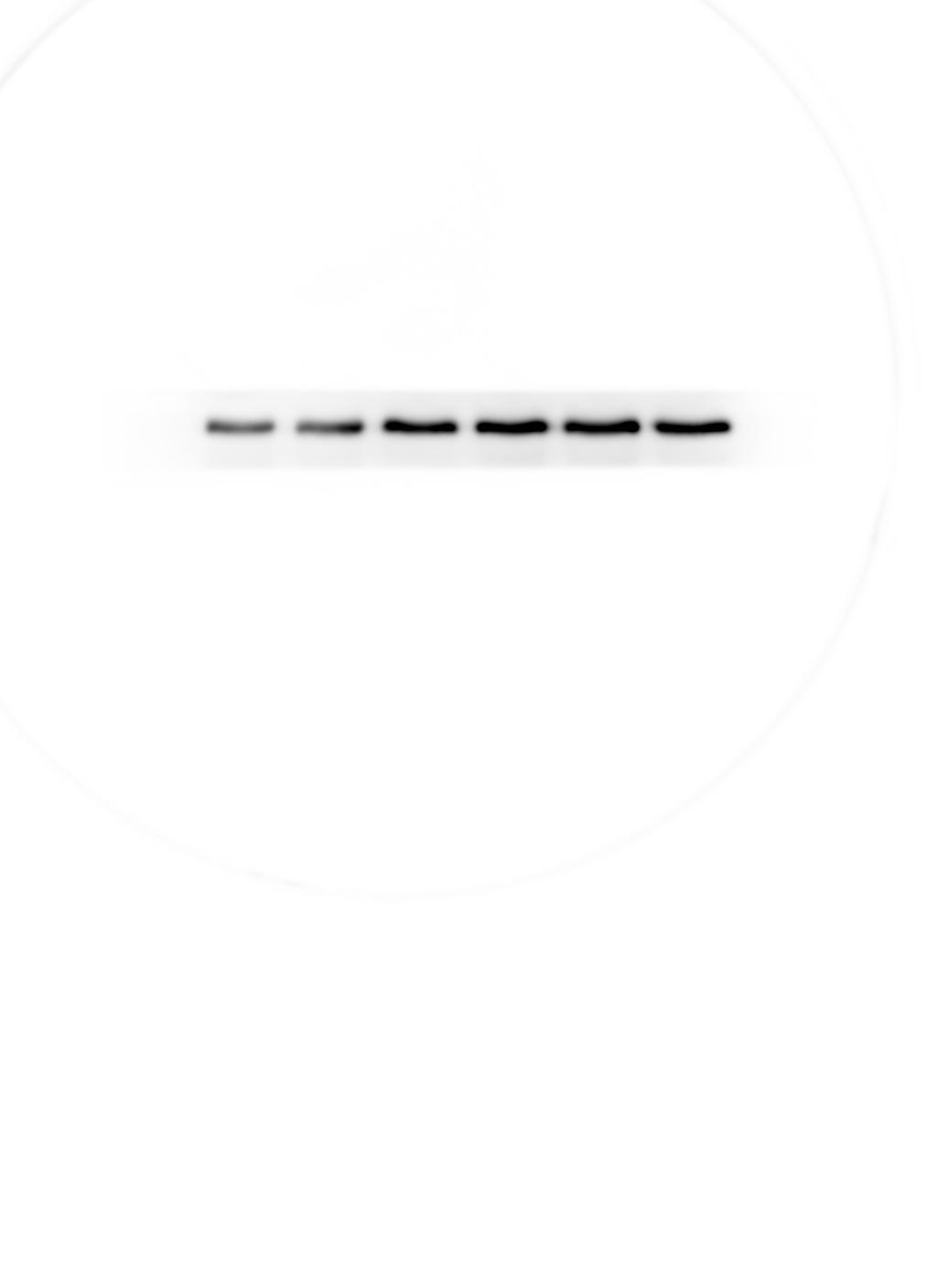

Supplement: Supplementary file 2 [file DataSheet_2.zip › raw original data-Fig3/WB/Fig3F-BECN1.jpg]

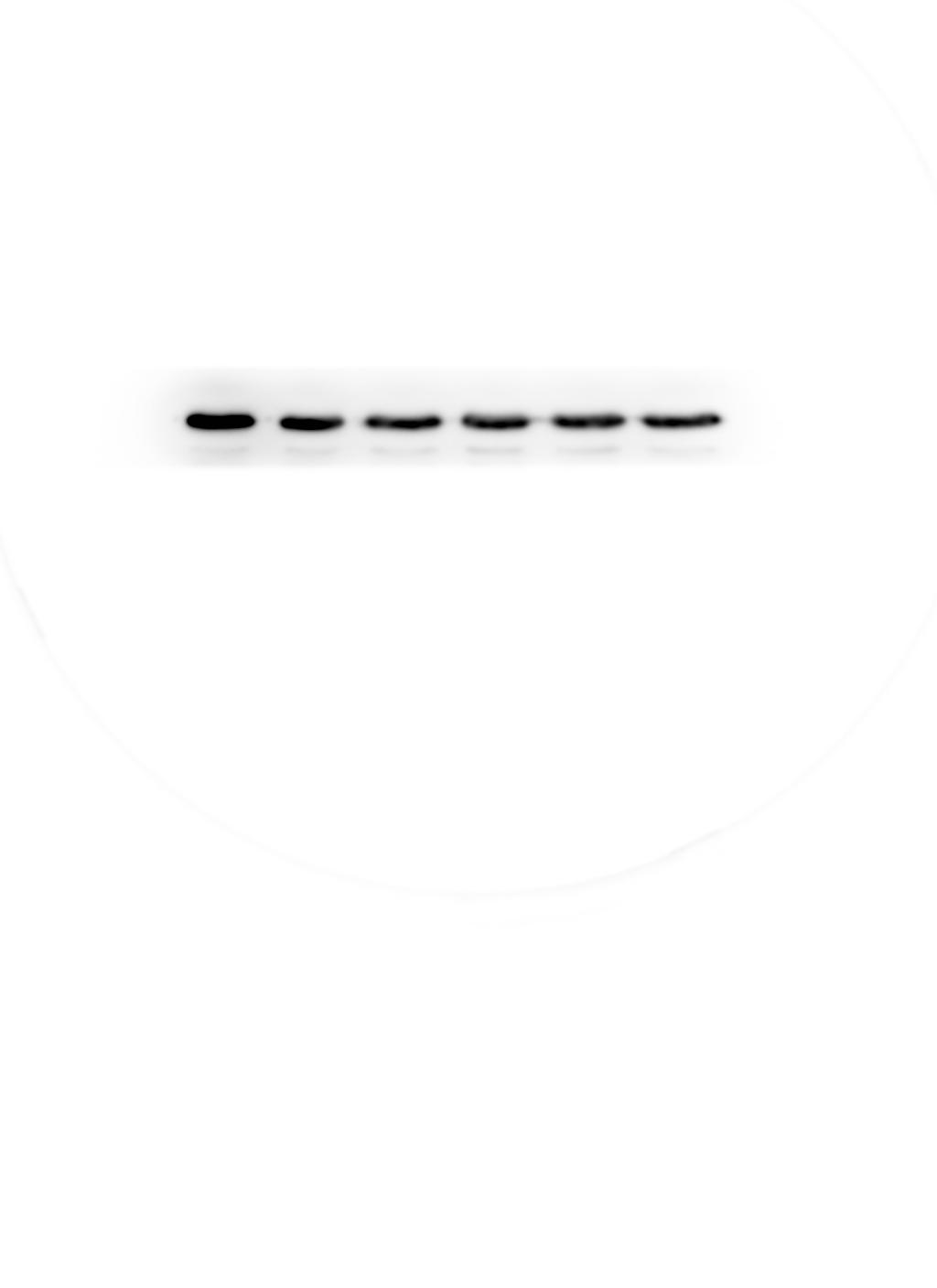

Supplement: Supplementary file 2 [file DataSheet_2.zip › raw original data-Fig3/WB/Fig3F-GAPDH.jpg]

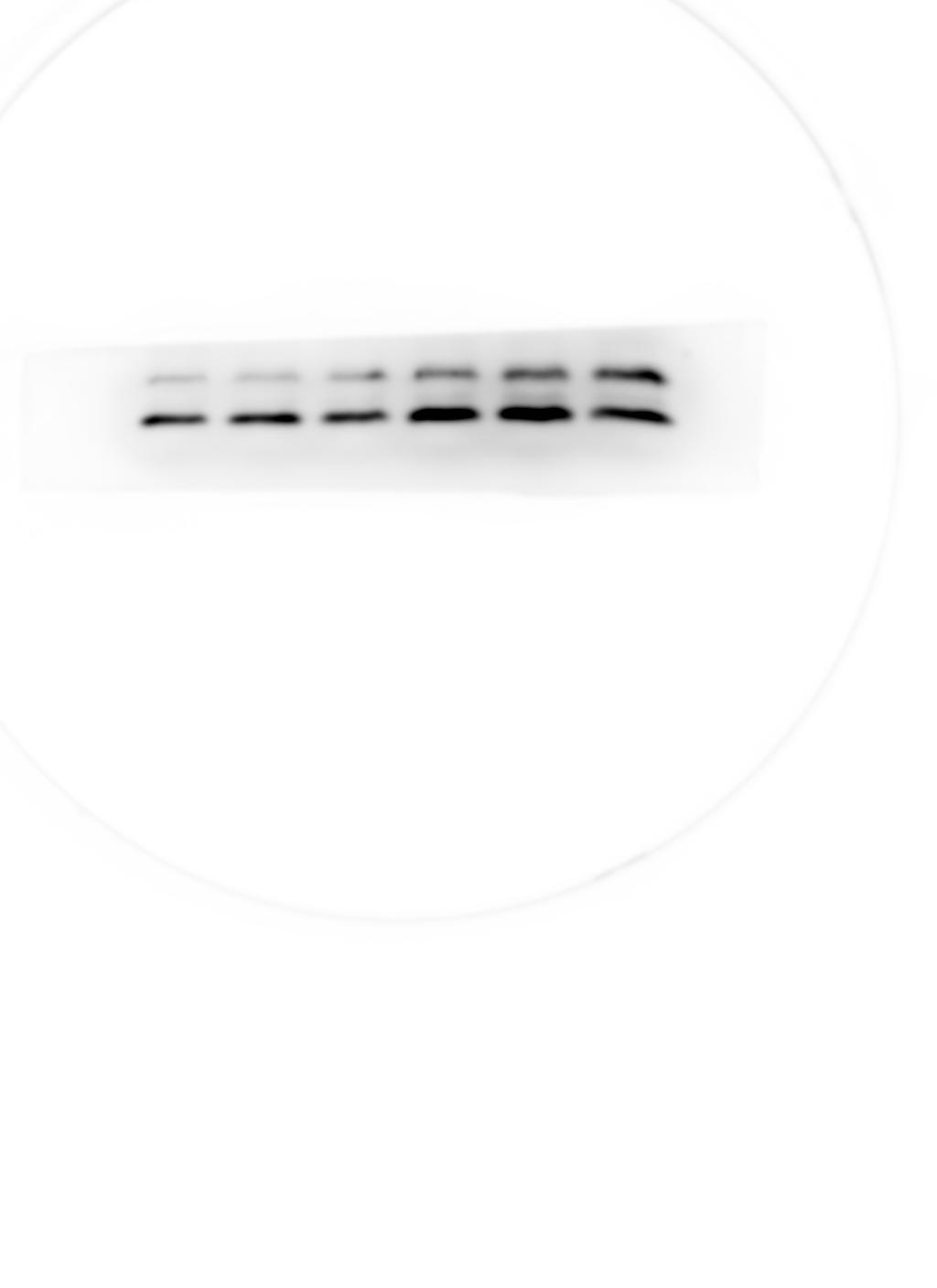

Supplement: Supplementary file 2 [file DataSheet_2.zip › raw original data-Fig3/WB/Fig3F-LC3B.jpg]

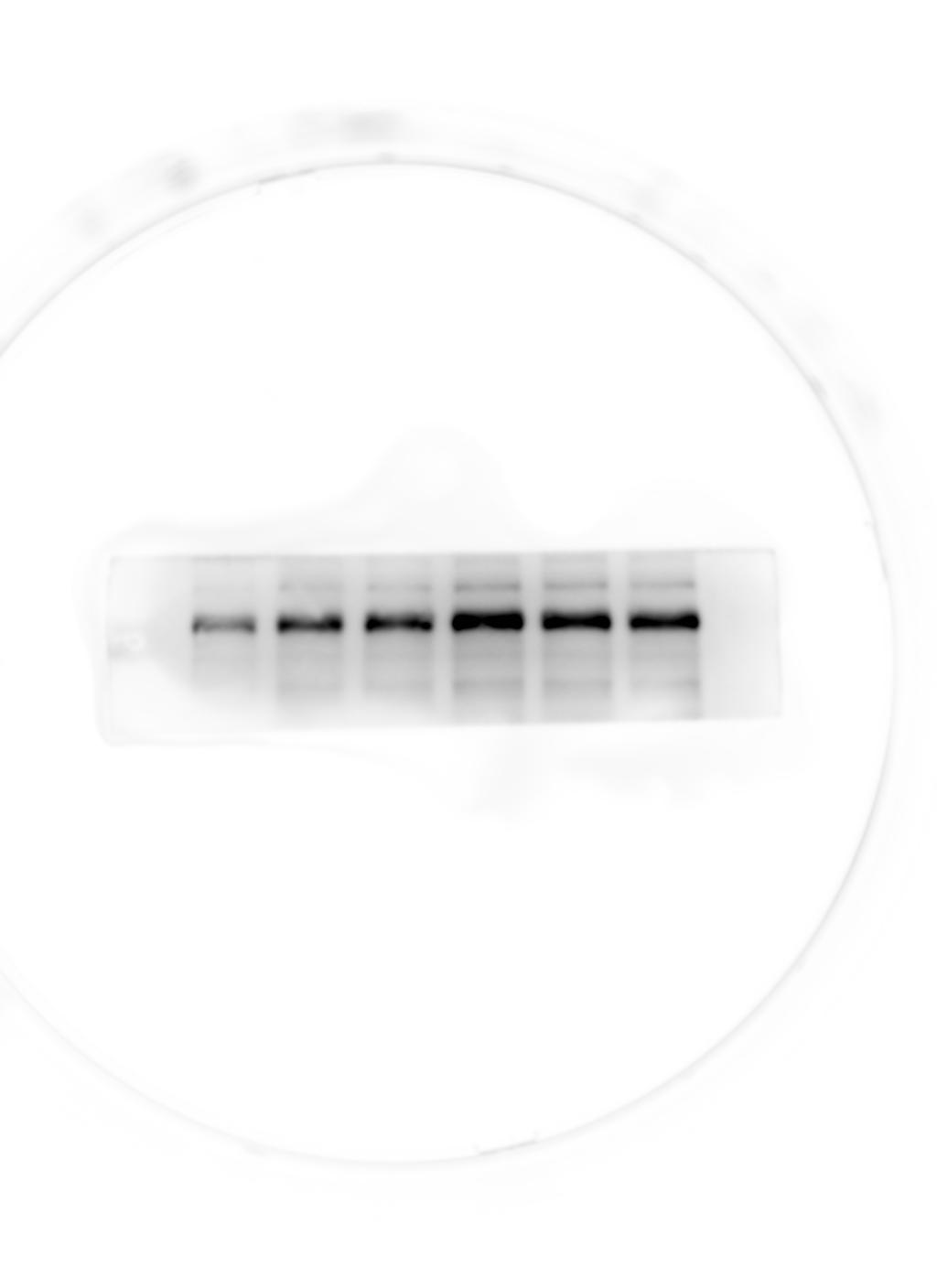

Supplement: Supplementary file 2 [file DataSheet_2.zip › raw original data-Fig3/WB/Fig3F-PR.jpg]

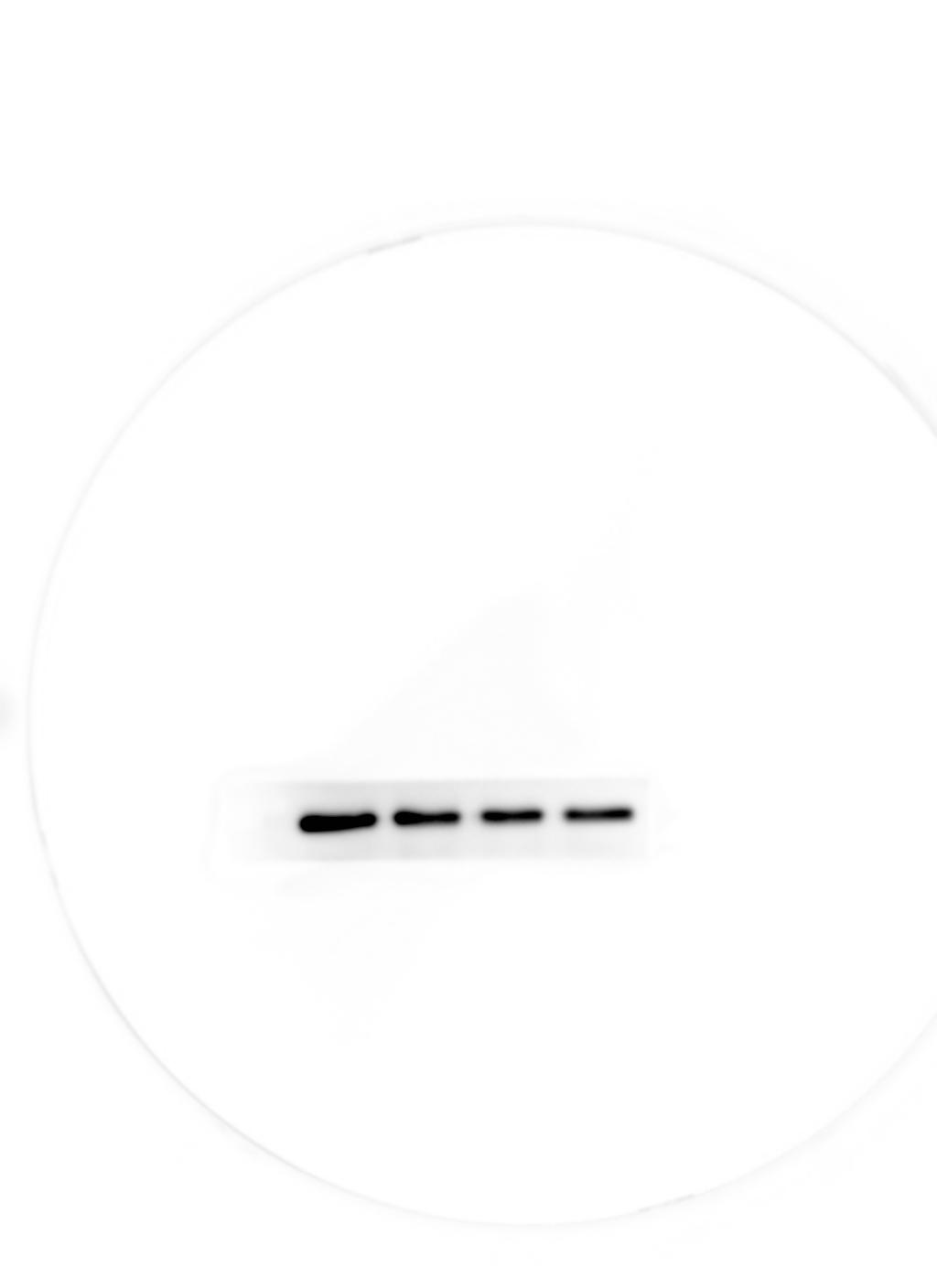

Supplement: Supplementary file 2 [file DataSheet_2.zip › raw original data-Fig3/WB/Fig3G-BECN1.jpg]

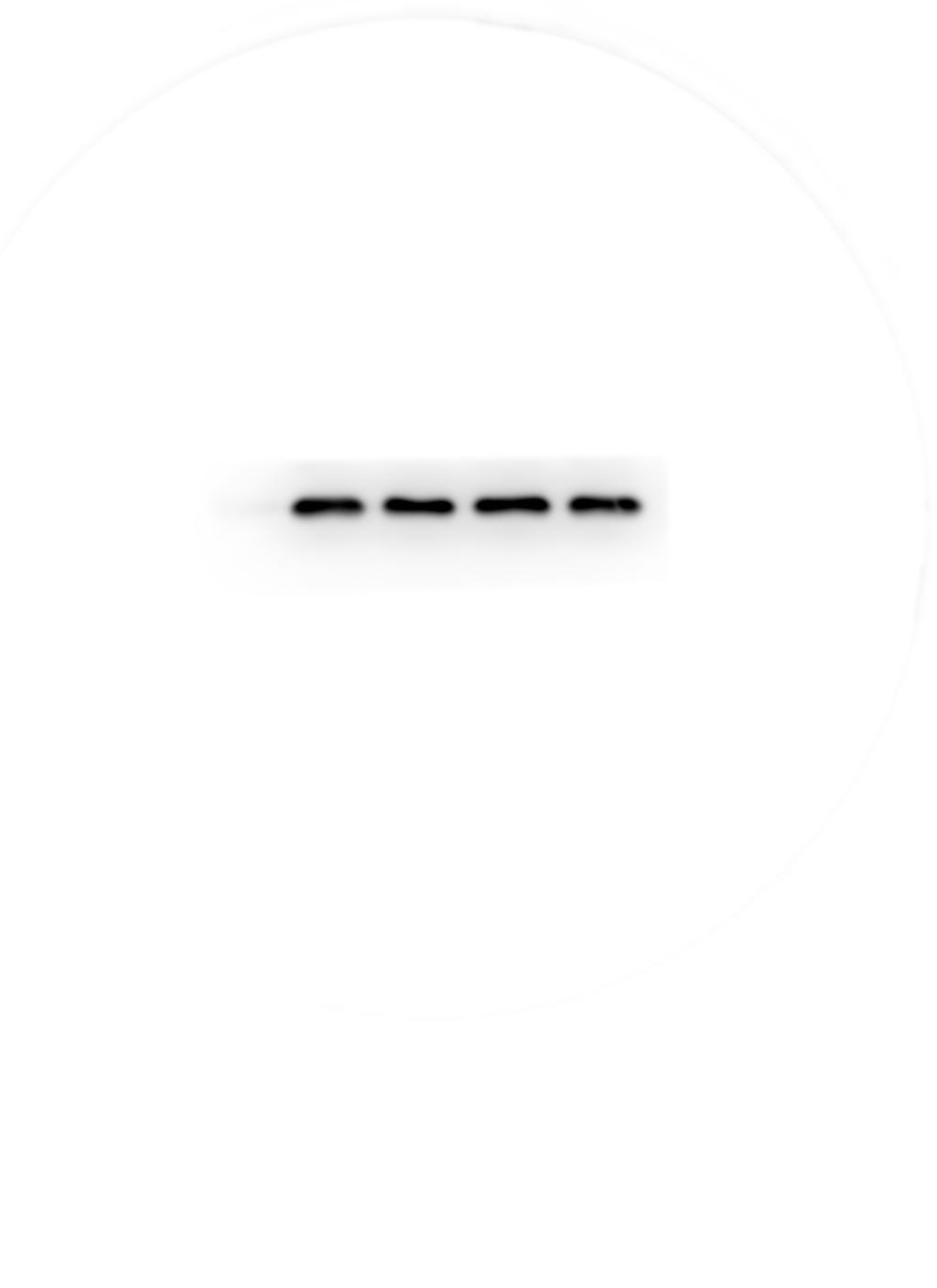

Supplement: Supplementary file 2 [file DataSheet_2.zip › raw original data-Fig3/WB/Fig3G-GAPDH.jpg]

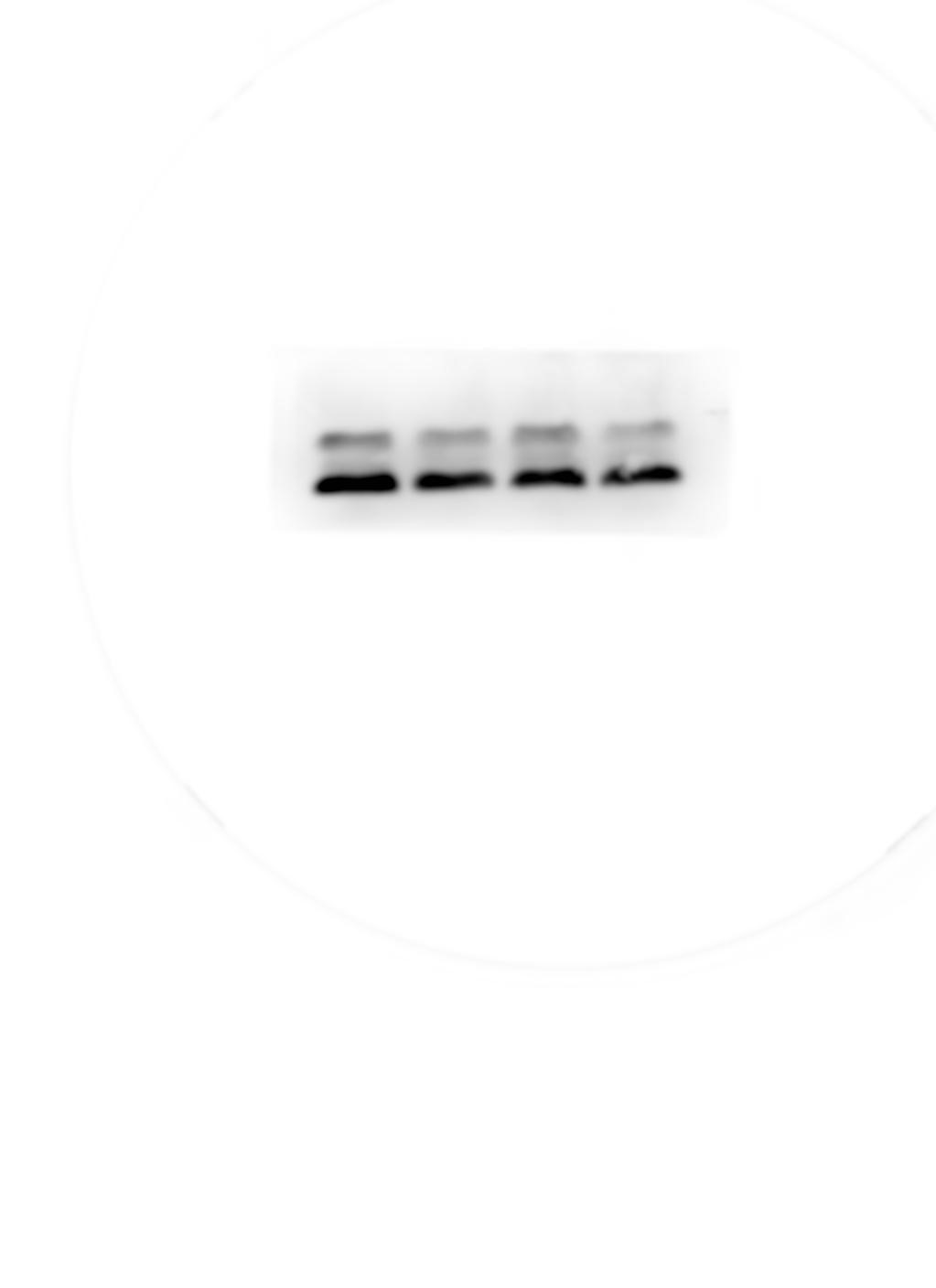

Supplement: Supplementary file 2 [file DataSheet_2.zip › raw original data-Fig3/WB/Fig3G-LC3B.jpg]

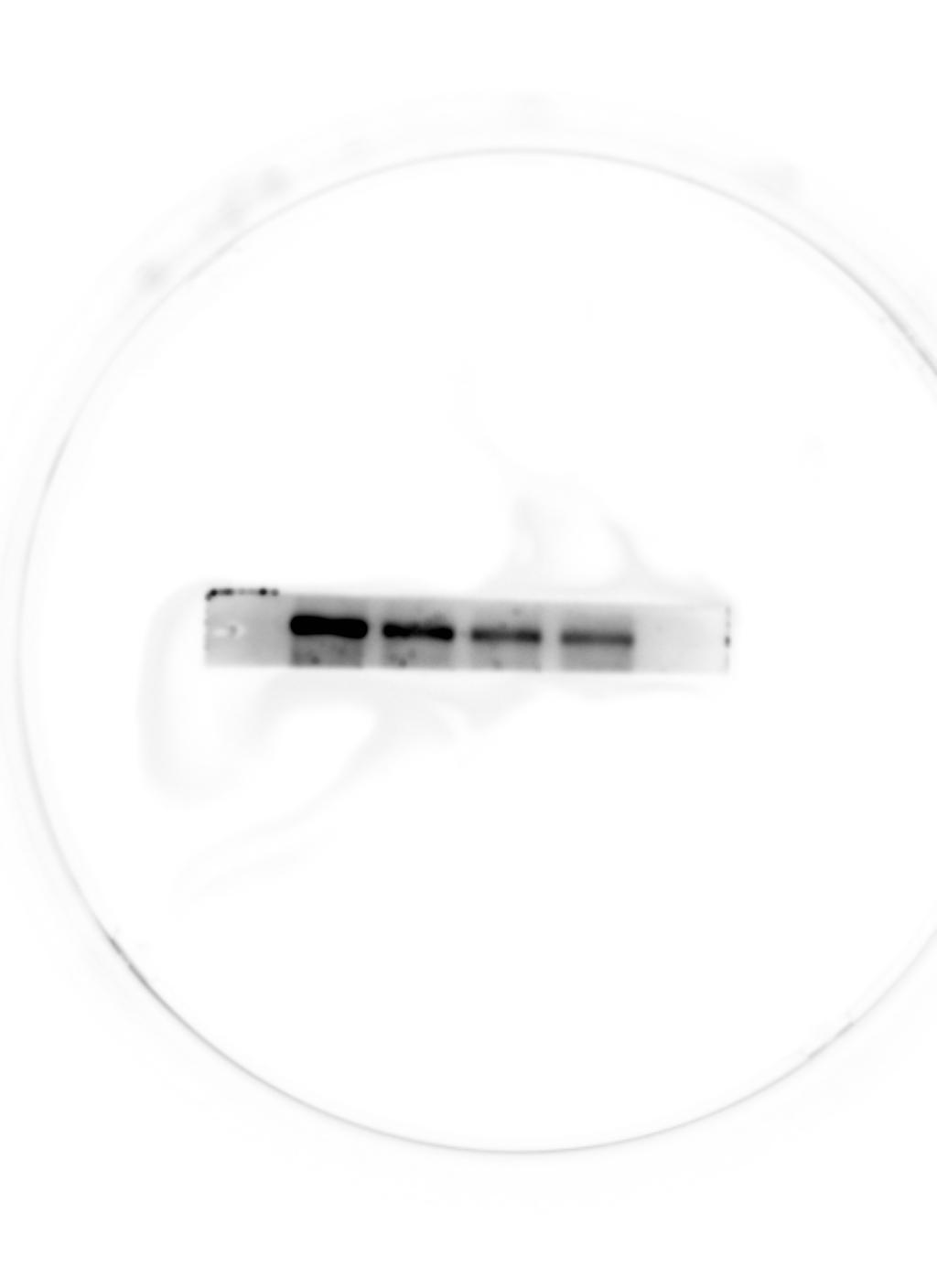

Supplement: Supplementary file 2 [file DataSheet_2.zip › raw original data-Fig3/WB/Fig3G-PR.jpg]

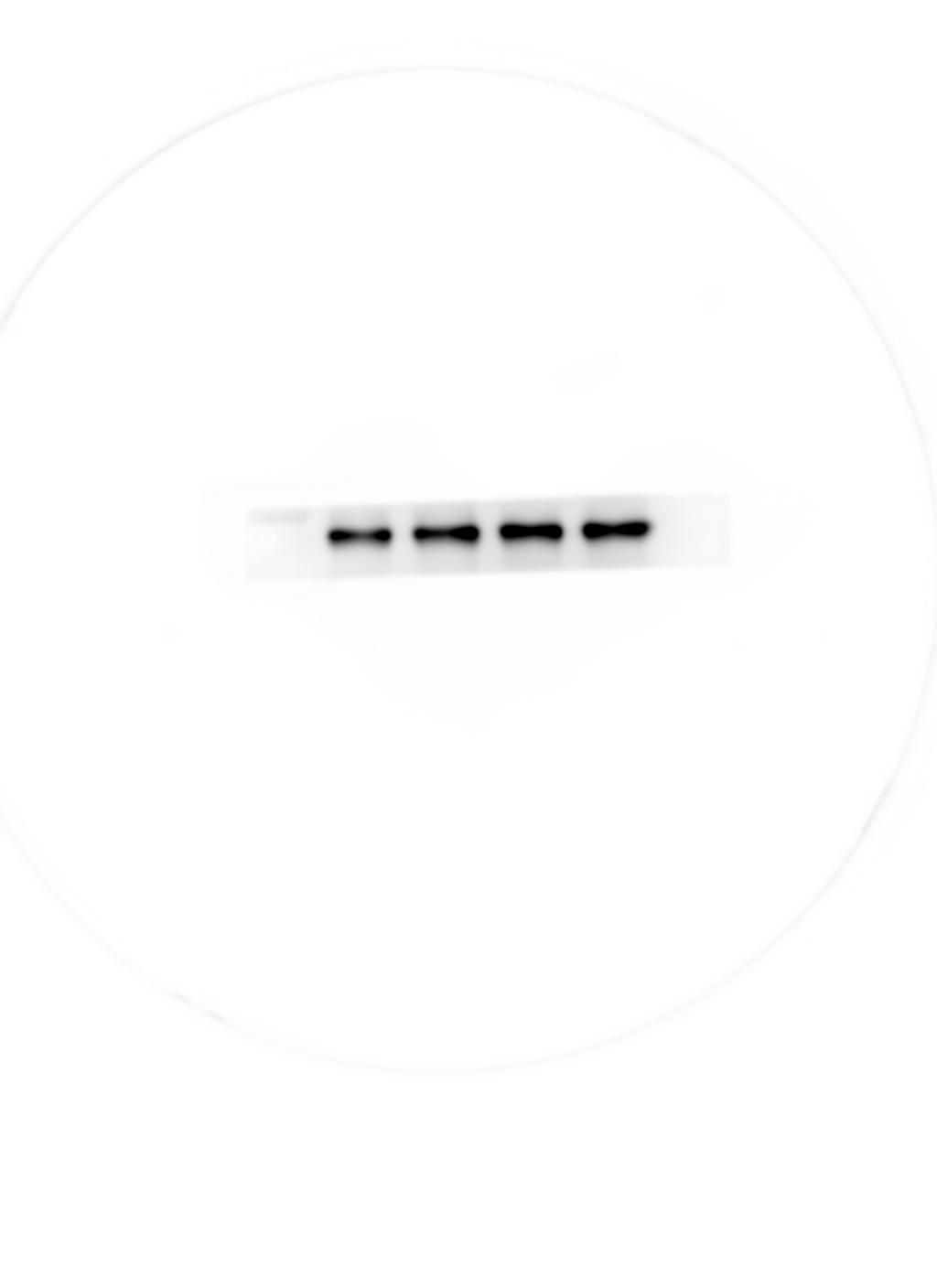

Supplement: Supplementary file 3 [file DataSheet_3.zip › raw original data-Fig4/WB/Fig4B-BECN1.jpg]

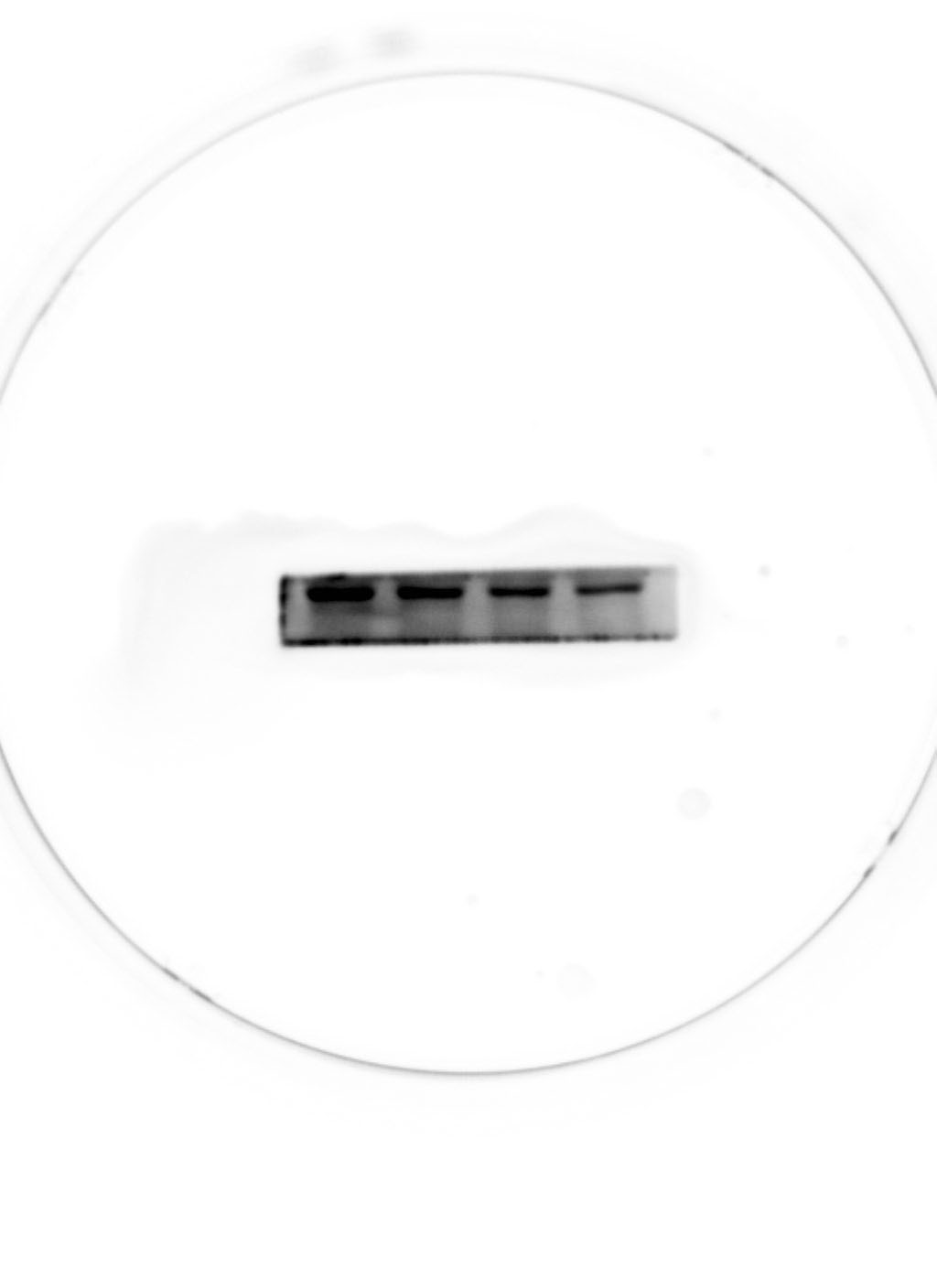

Supplement: Supplementary file 3 [file DataSheet_3.zip › raw original data-Fig4/WB/Fig4B-ERa.jpg]

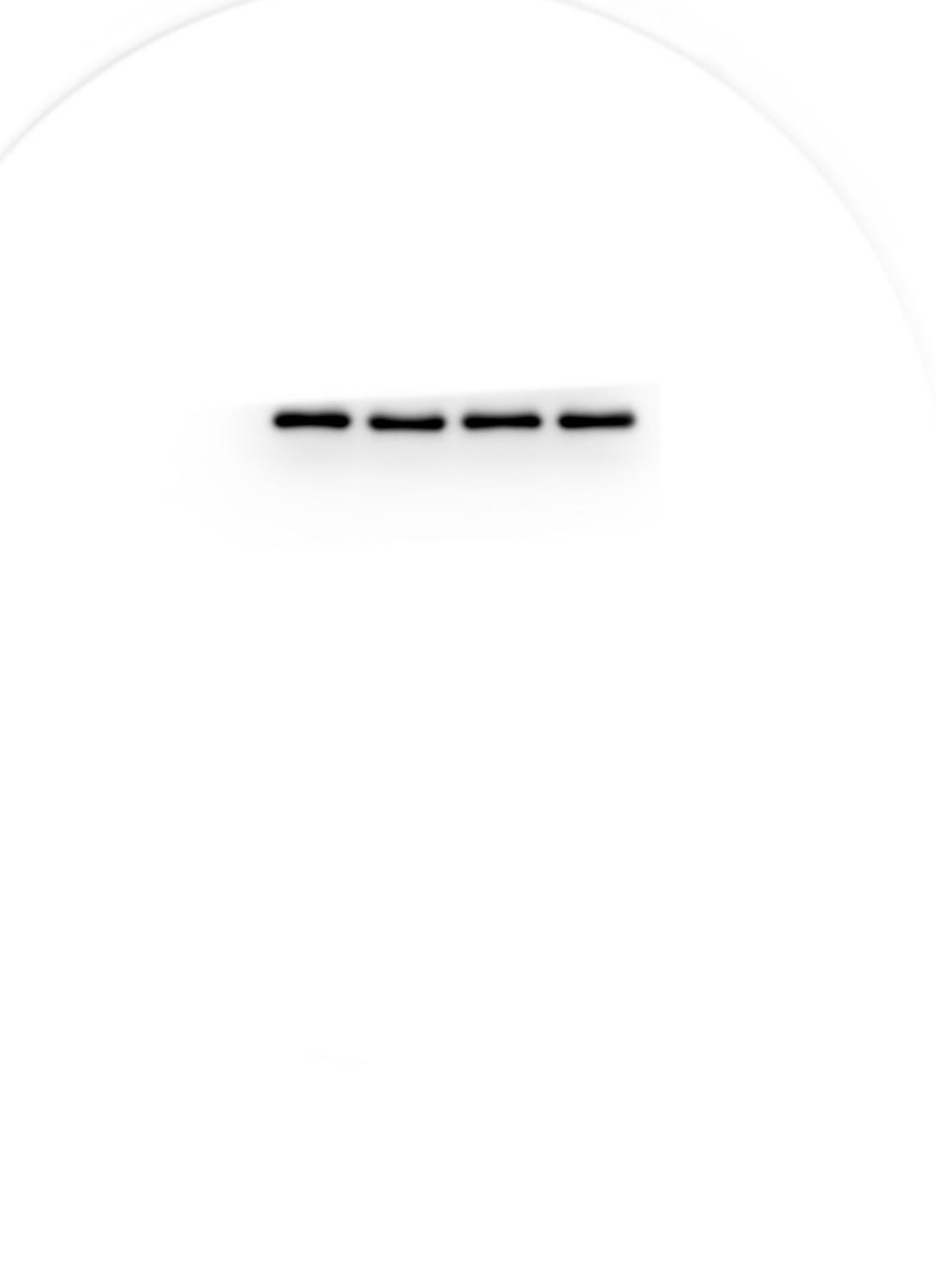

Supplement: Supplementary file 3 [file DataSheet_3.zip › raw original data-Fig4/WB/Fig4B-GAPDH.jpg]

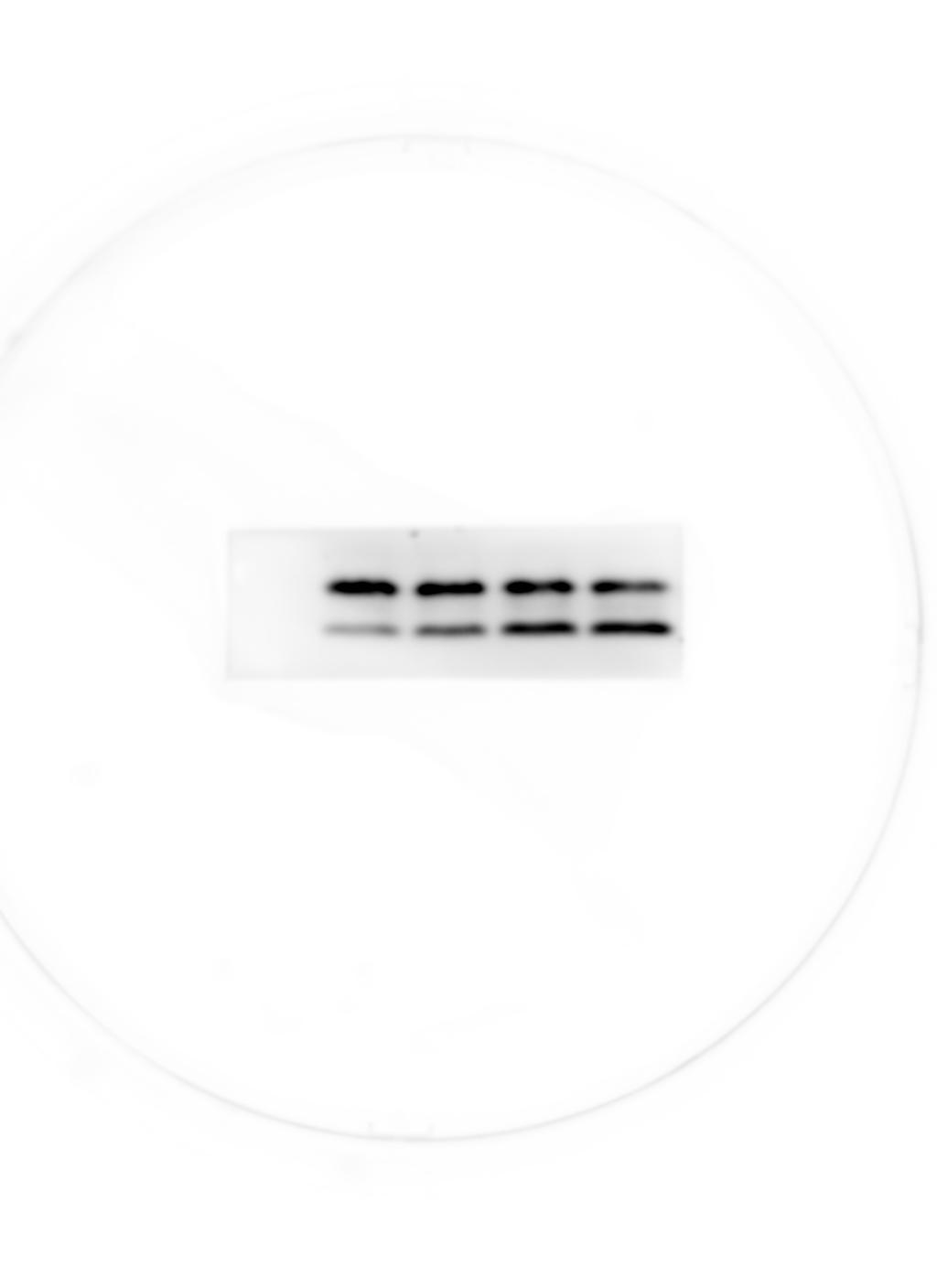

Supplement: Supplementary file 3 [file DataSheet_3.zip › raw original data-Fig4/WB/Fig4B-LC3B.jpg]

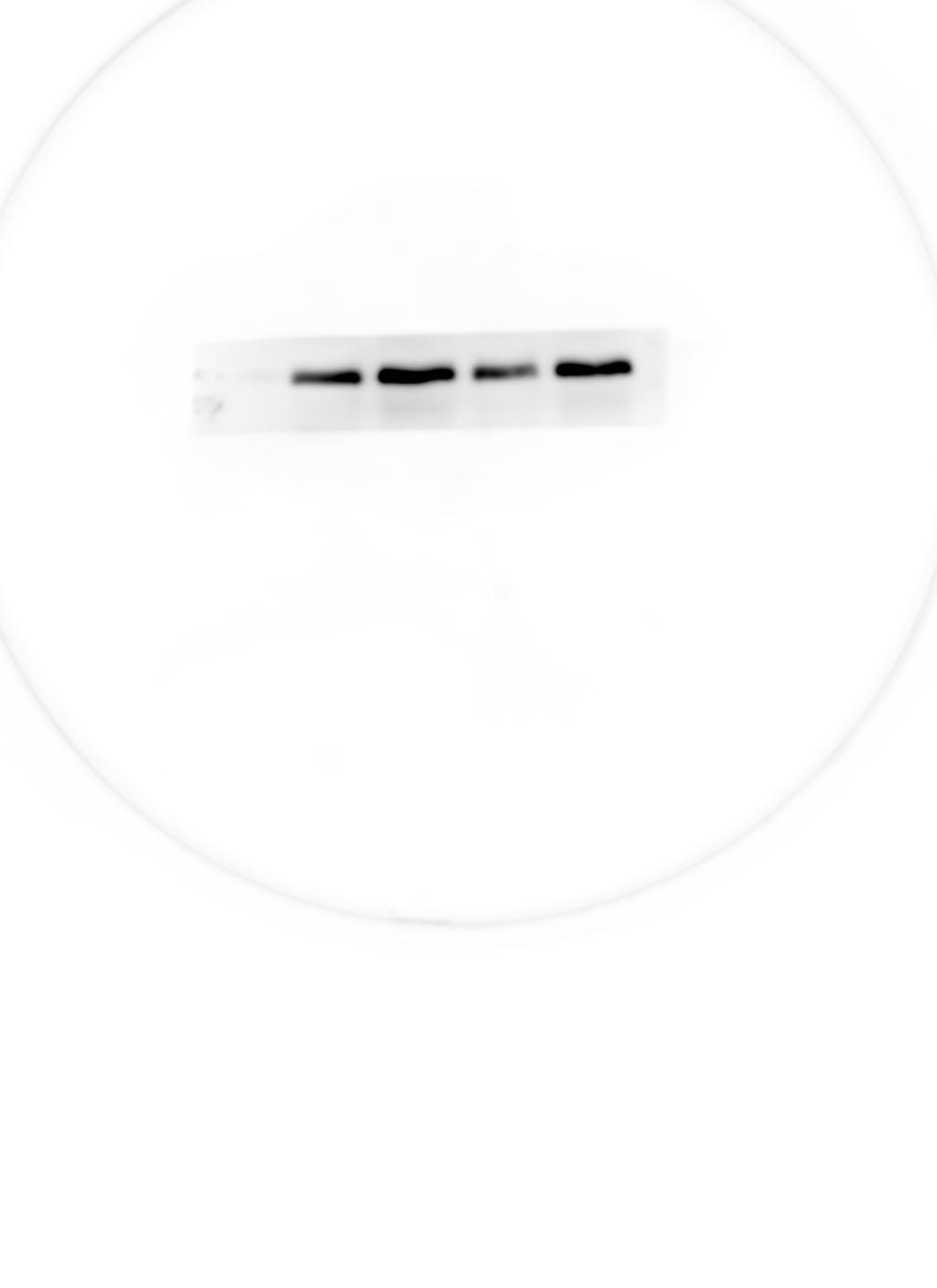

Supplement: Supplementary file 3 [file DataSheet_3.zip › raw original data-Fig4/WB/Fig4C-BECN1.jpg]

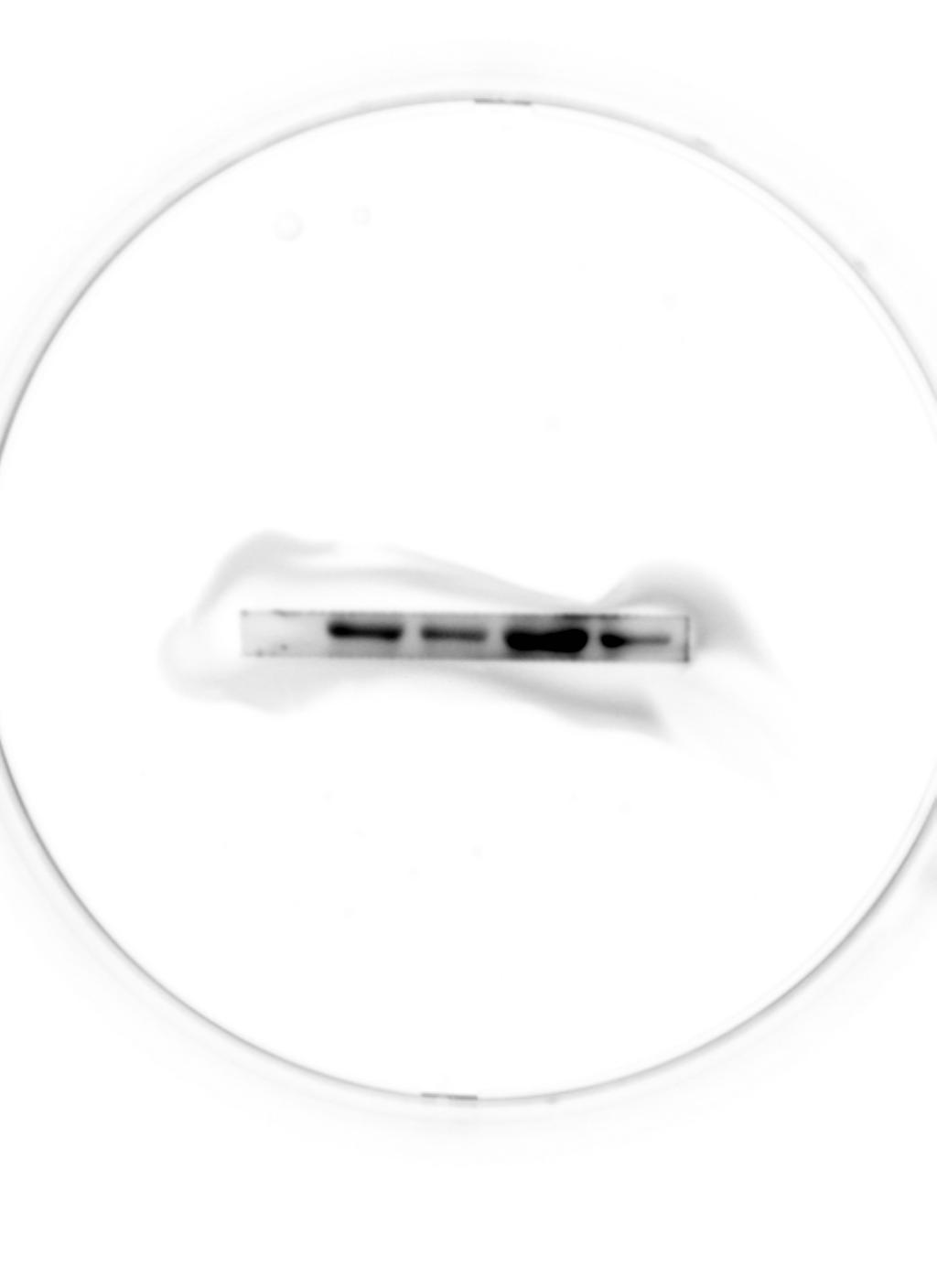

Supplement: Supplementary file 3 [file DataSheet_3.zip › raw original data-Fig4/WB/Fig4C-ERa.jpg]

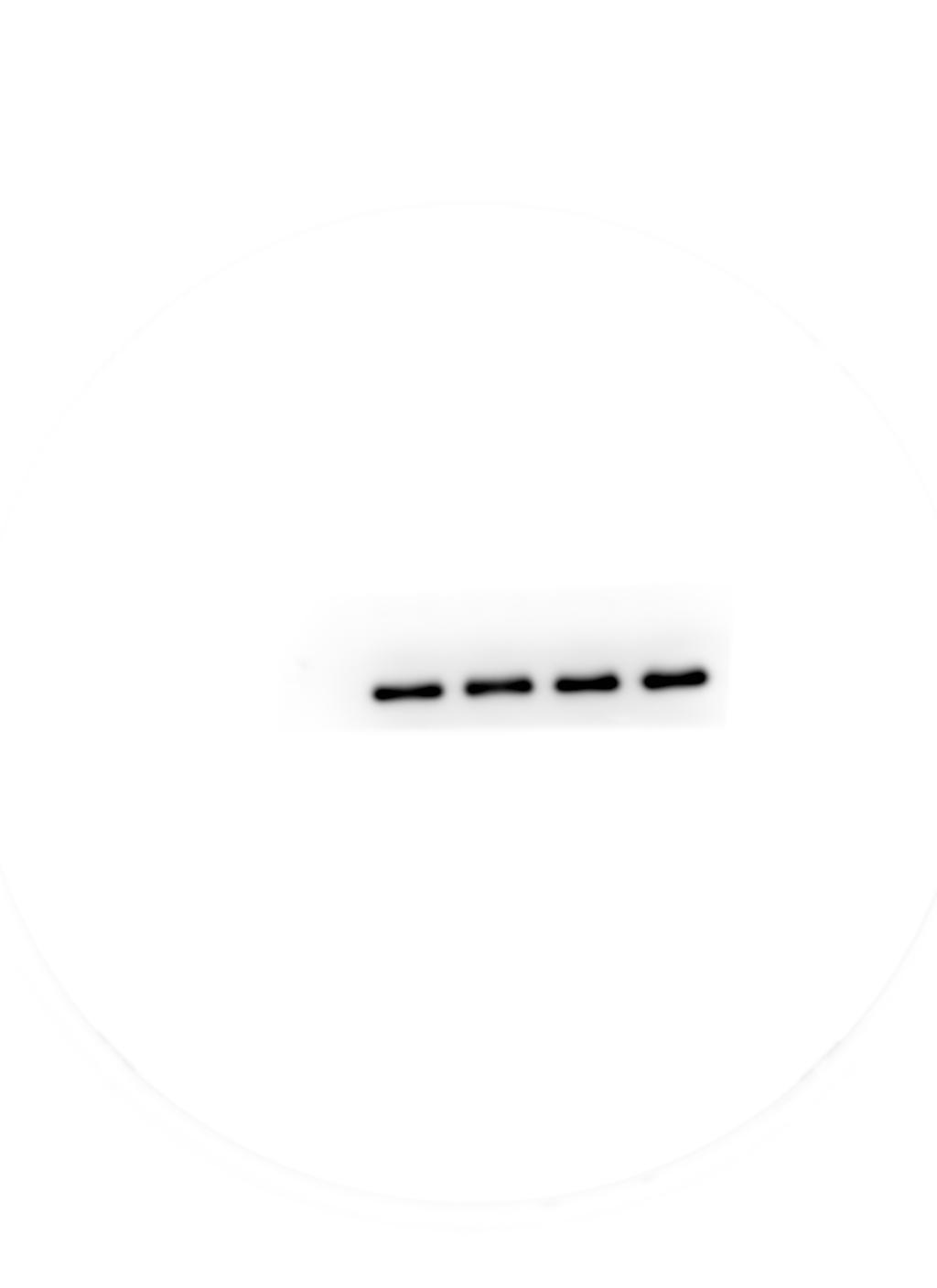

Supplement: Supplementary file 3 [file DataSheet_3.zip › raw original data-Fig4/WB/Fig4C-GAPDH.jpg]

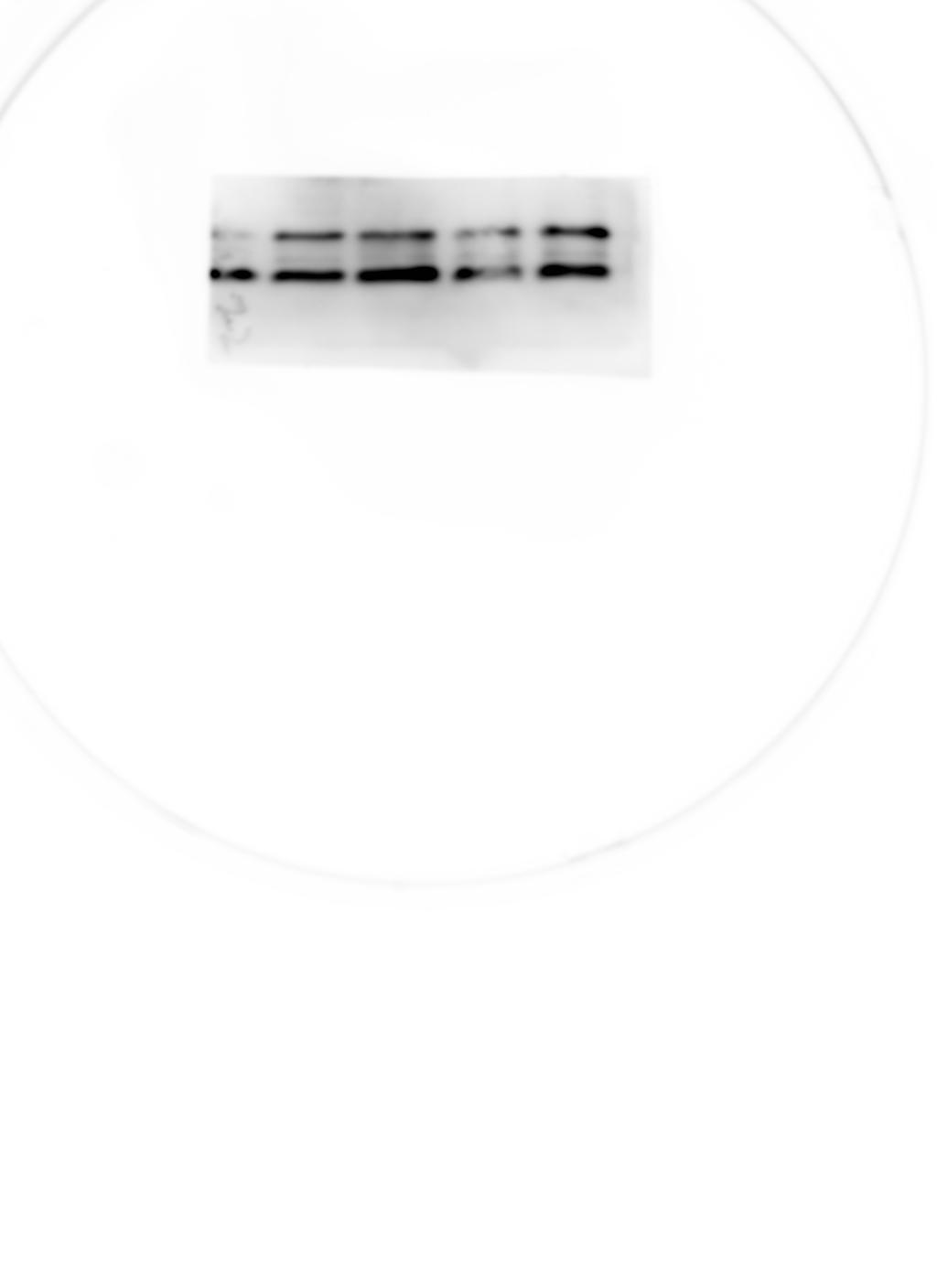

Supplement: Supplementary file 3 [file DataSheet_3.zip › raw original data-Fig4/WB/Fig4C-LC3B.jpg]

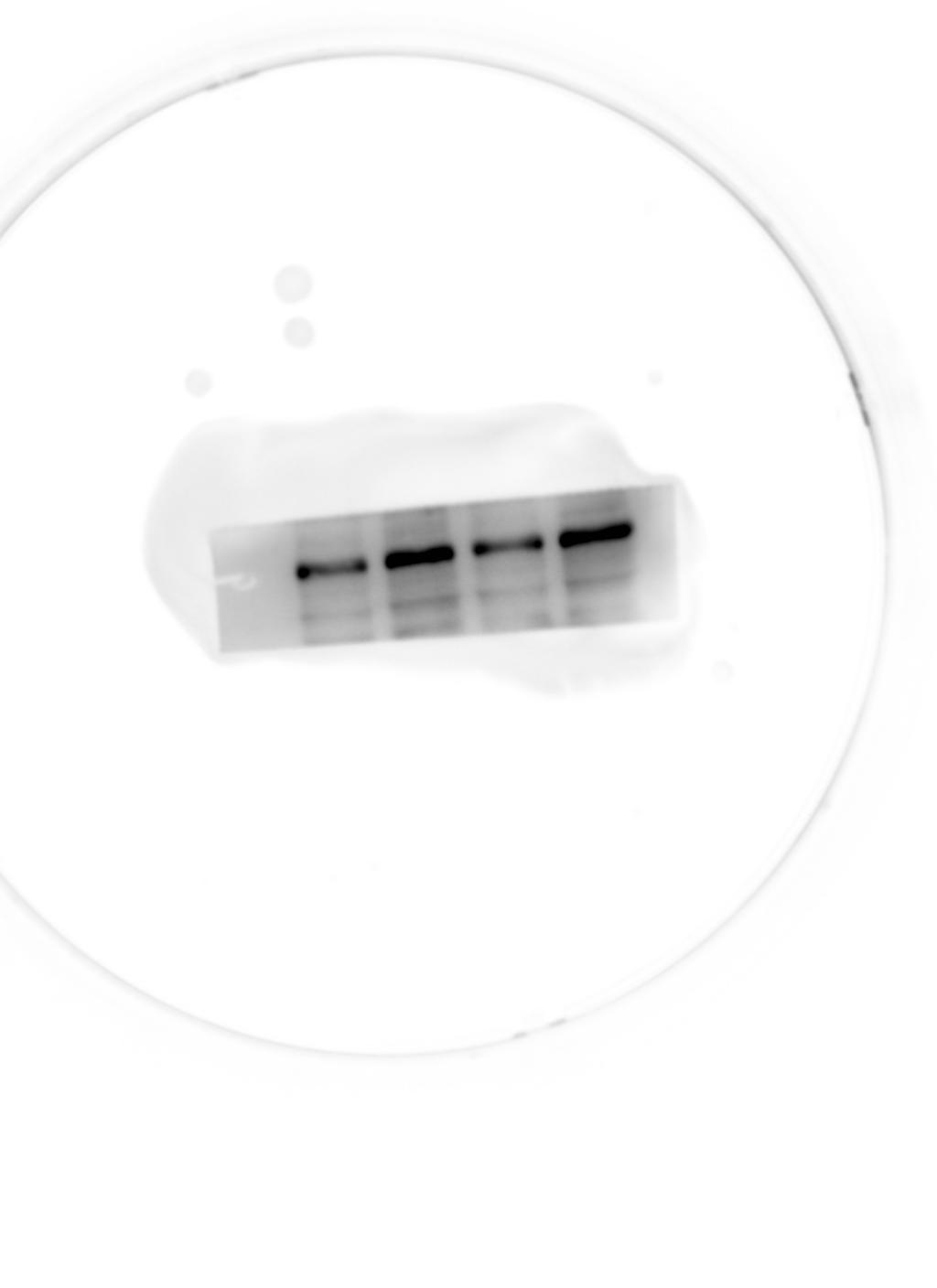

Supplement: Supplementary file 3 [file DataSheet_3.zip › raw original data-Fig4/WB/Fig4C-PR.jpg]

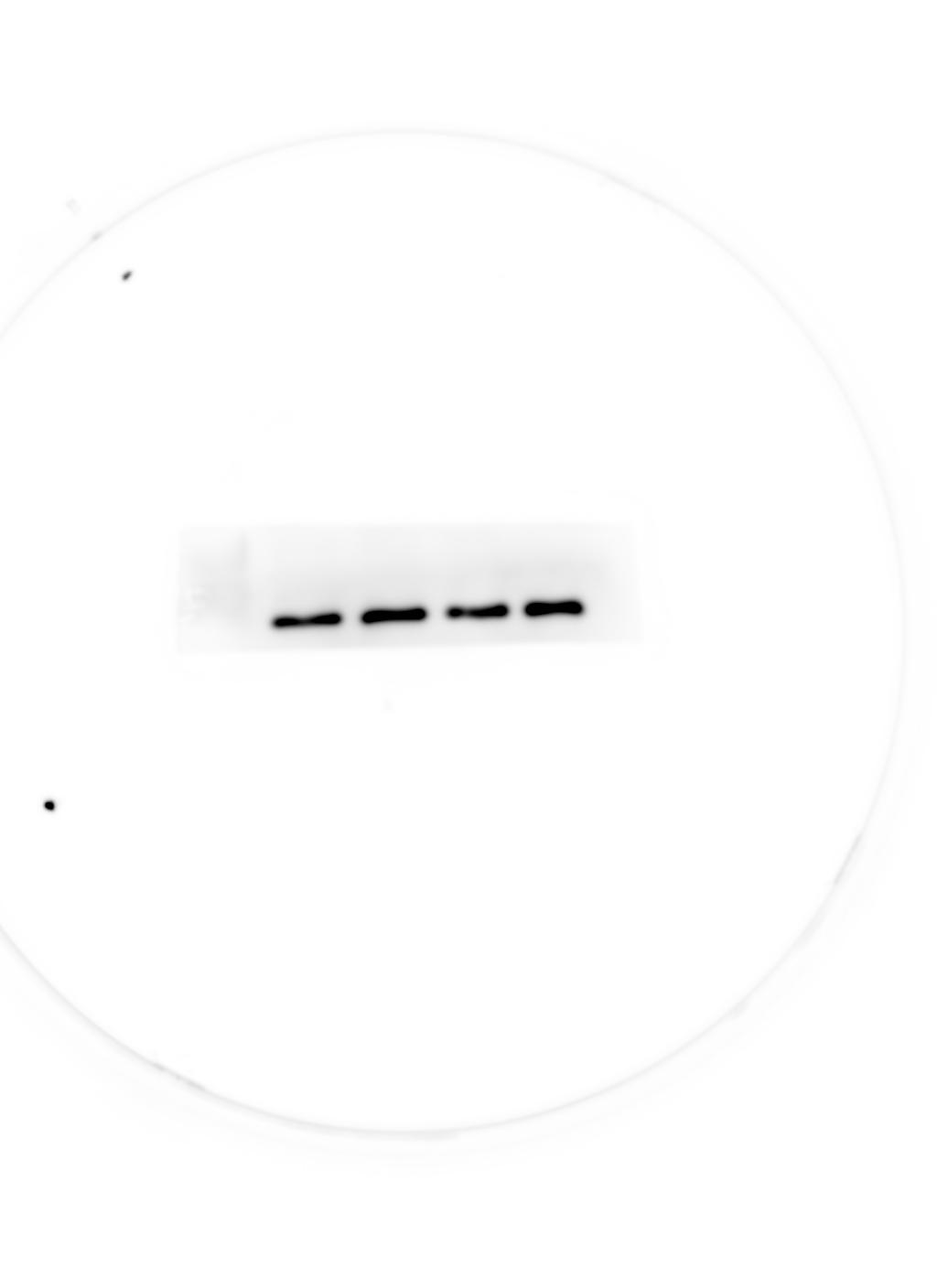

Supplement: Supplementary file 3 [file DataSheet_3.zip › raw original data-Fig4/WB/Fig4D-GAPDH.jpg]

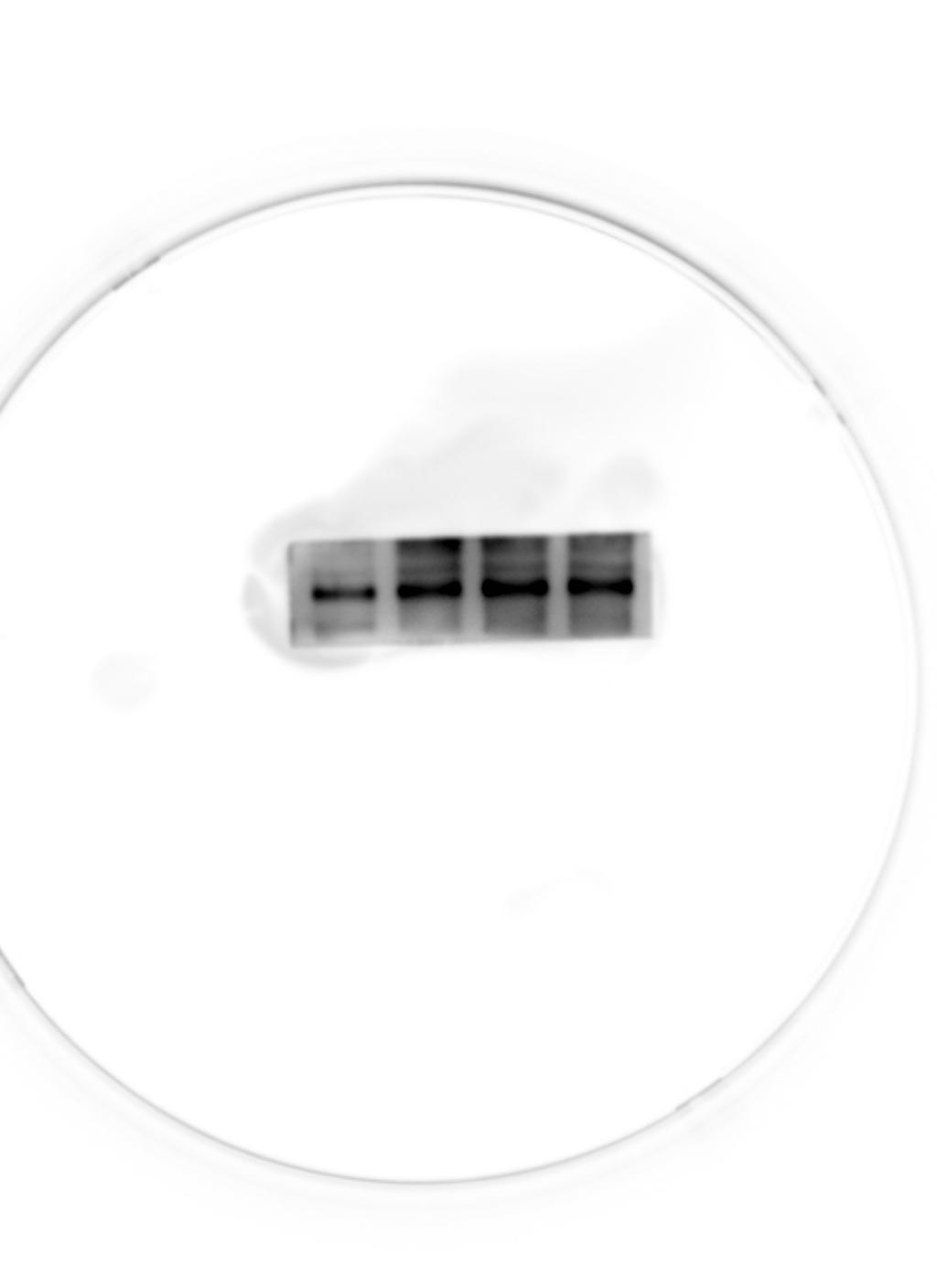

Supplement: Supplementary file 3 [file DataSheet_3.zip › raw original data-Fig4/WB/Fig4D-PR.jpg]

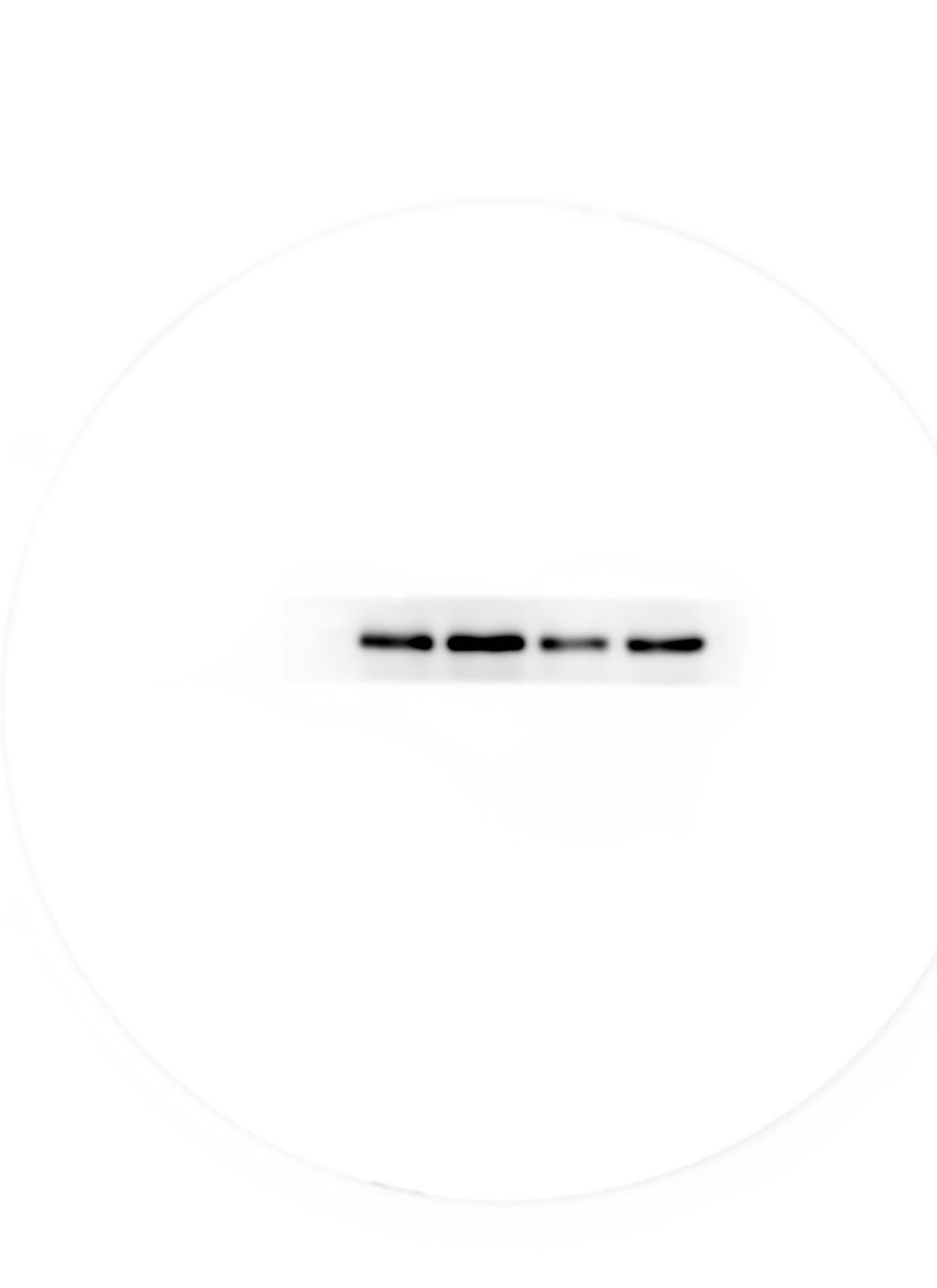

Supplement: Supplementary file 3 [file DataSheet_3.zip › raw original data-Fig4/WB/Fig4E-BECN1.jpg]

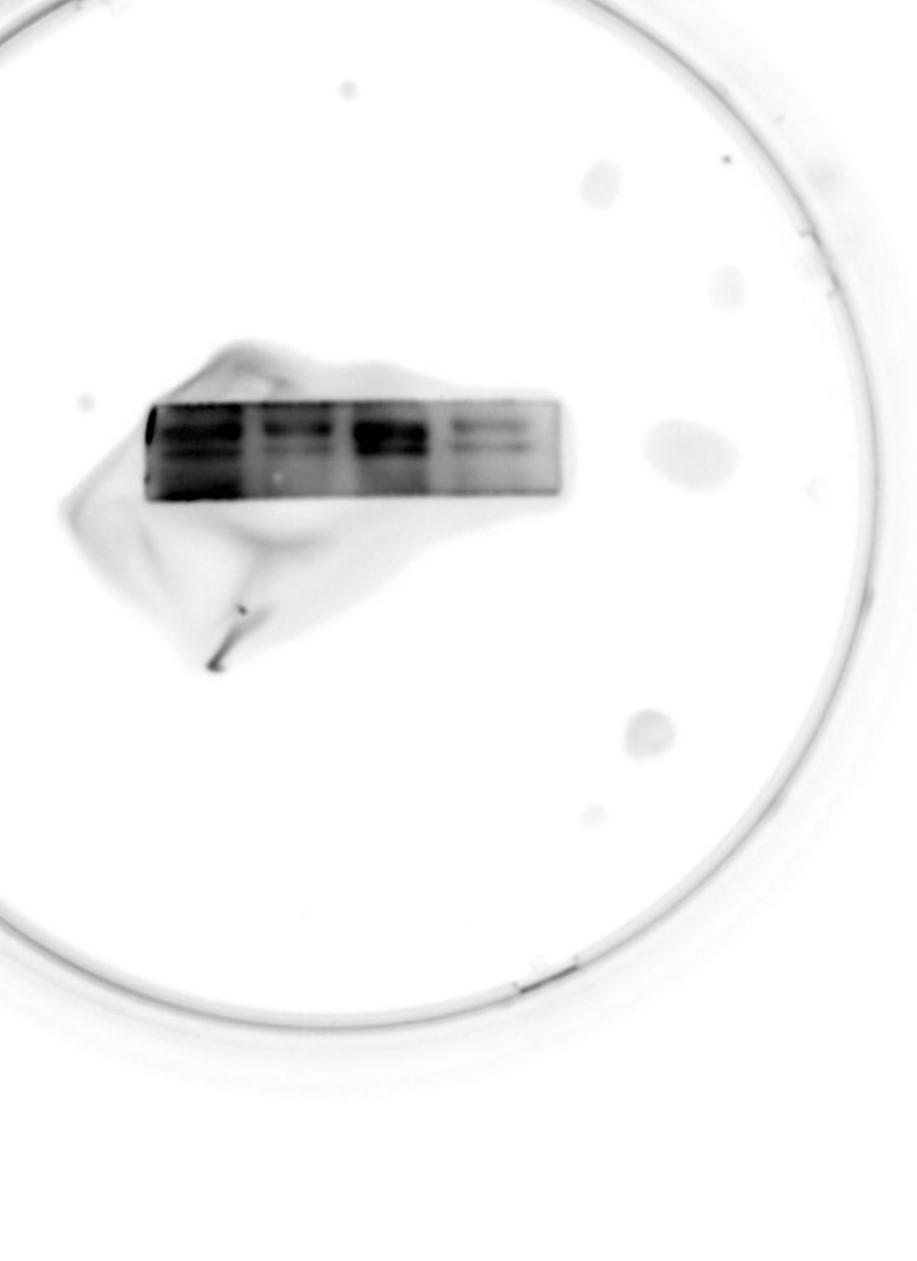

Supplement: Supplementary file 3 [file DataSheet_3.zip › raw original data-Fig4/WB/Fig4E-ERa.jpg]

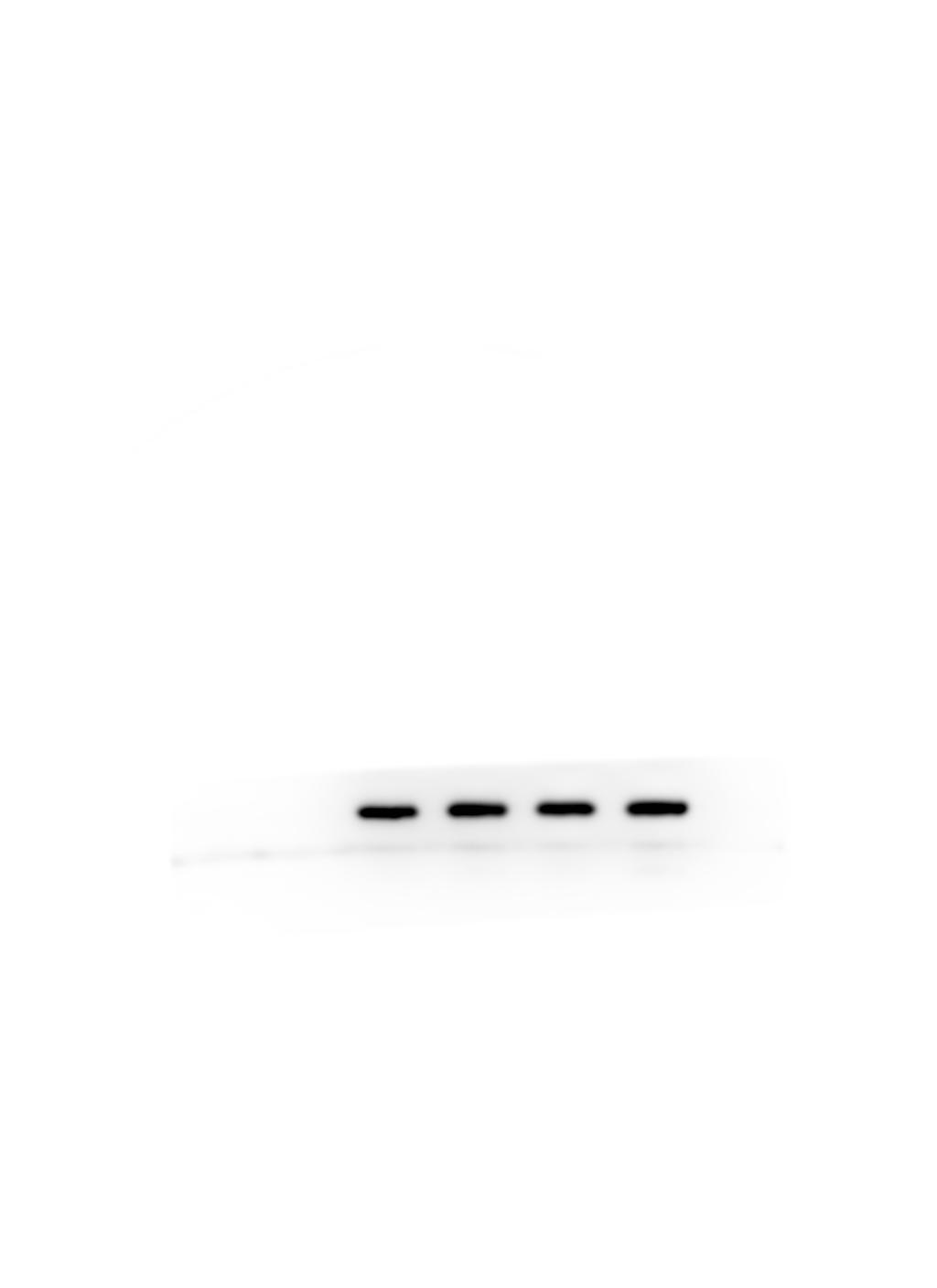

Supplement: Supplementary file 3 [file DataSheet_3.zip › raw original data-Fig4/WB/Fig4E-GAPDH.jpg]

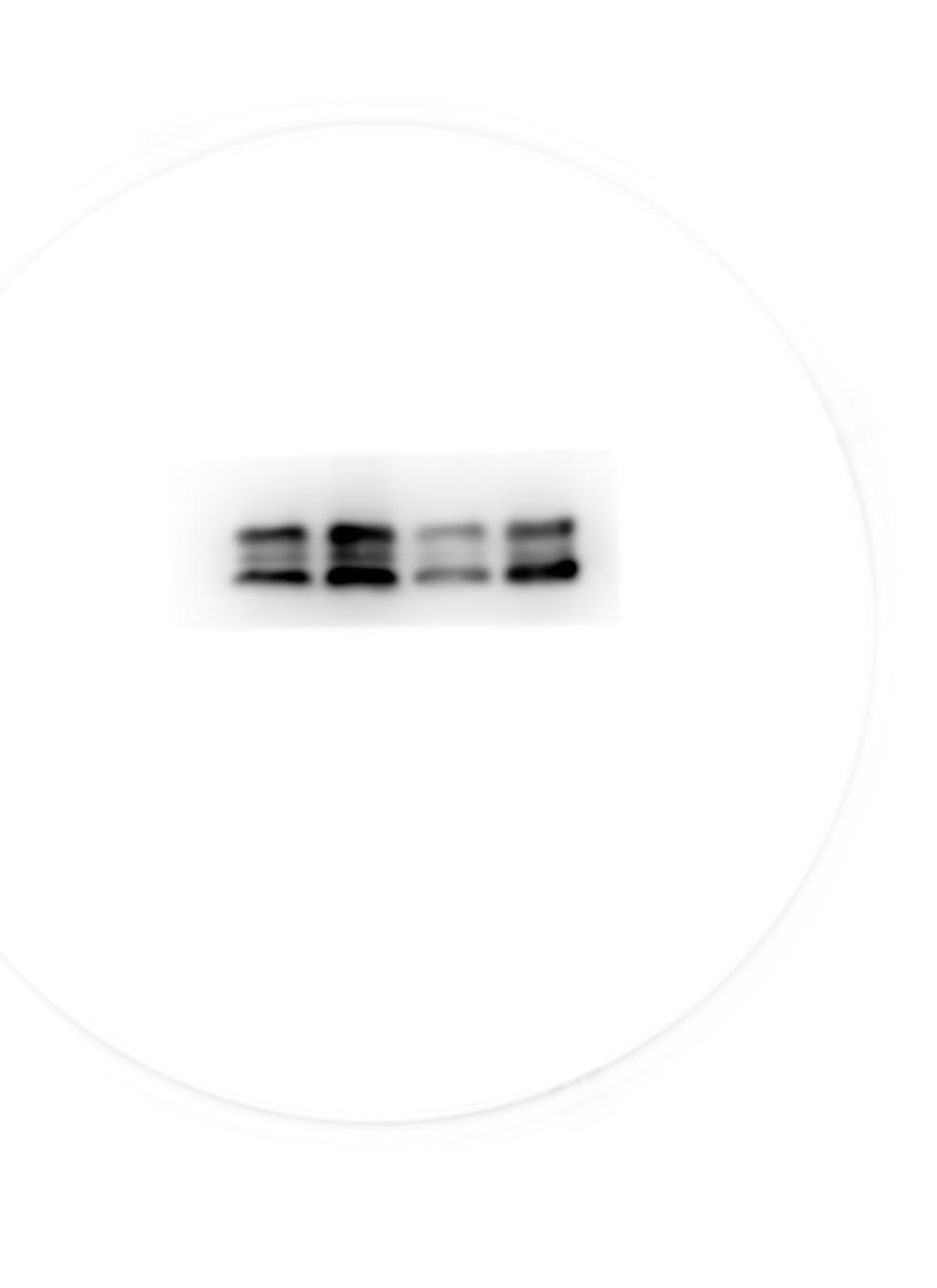

Supplement: Supplementary file 3 [file DataSheet_3.zip › raw original data-Fig4/WB/Fig4E-LC3B.jpg]

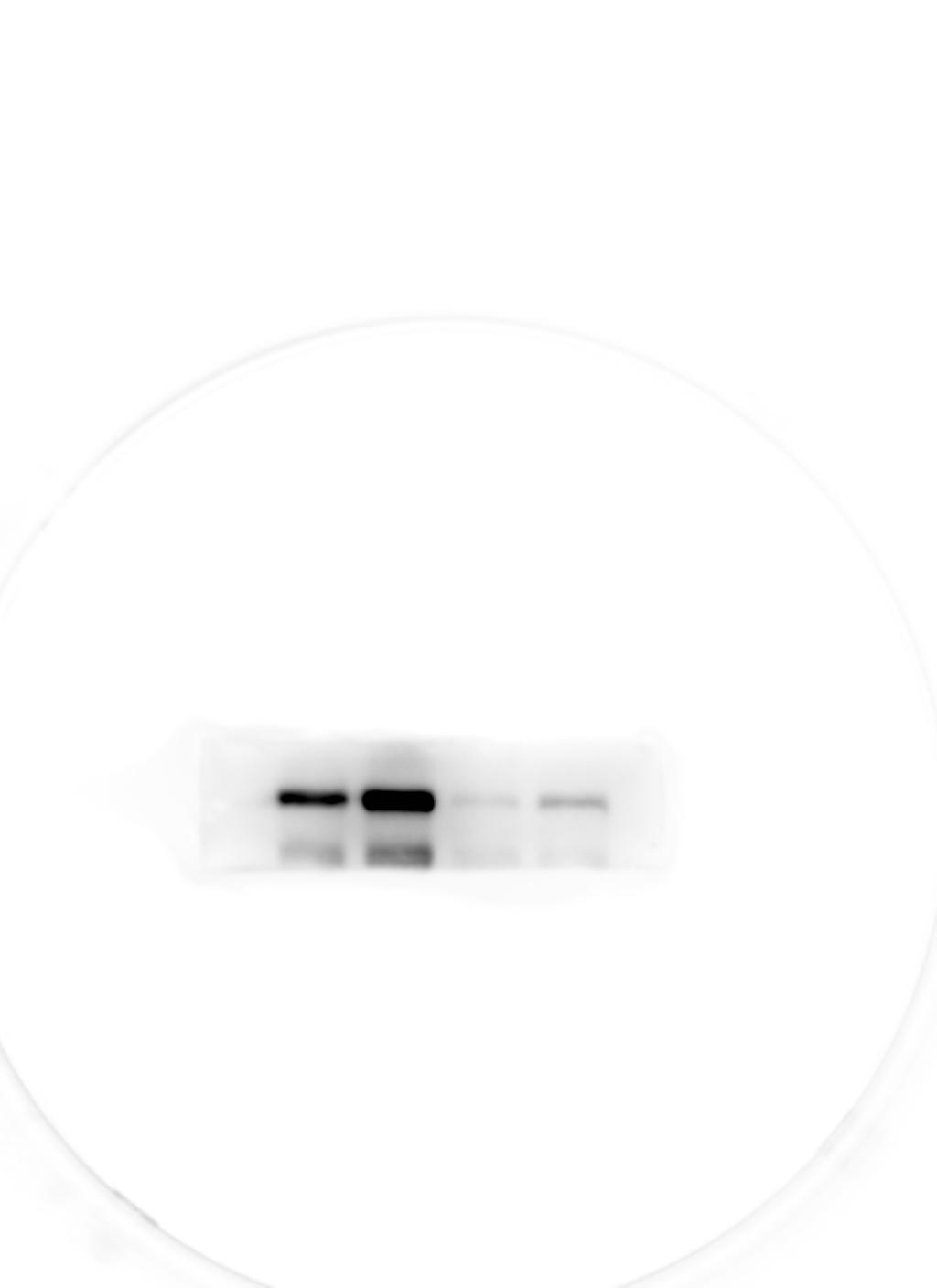

Supplement: Supplementary file 3 [file DataSheet_3.zip › raw original data-Fig4/WB/Fig4E-PR.jpg]

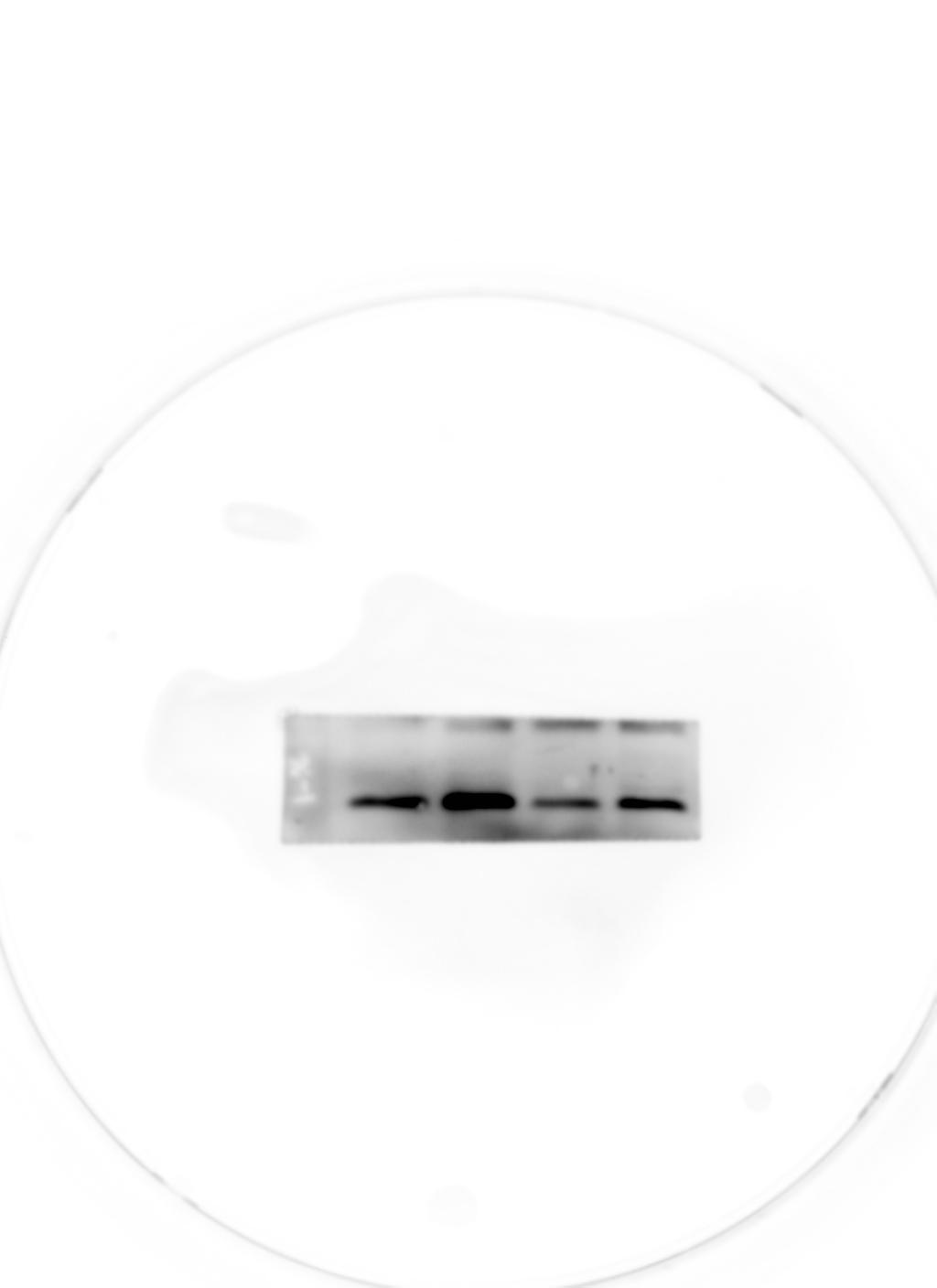

Supplement: Supplementary file 3 [file DataSheet_3.zip › raw original data-Fig4/WB/Fig4F-Bax.jpg]

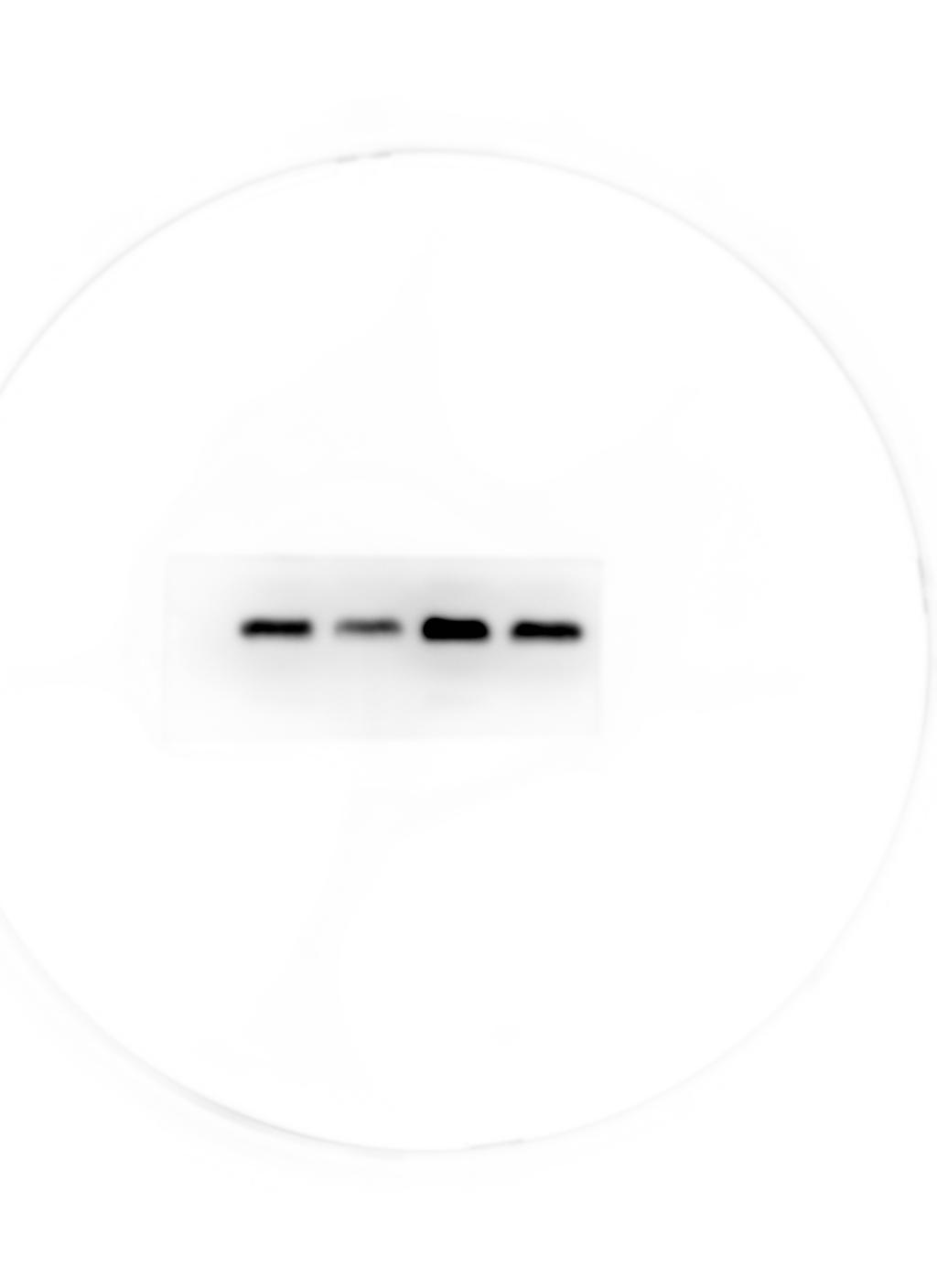

Supplement: Supplementary file 3 [file DataSheet_3.zip › raw original data-Fig4/WB/Fig4F-Bcl2.jpg]

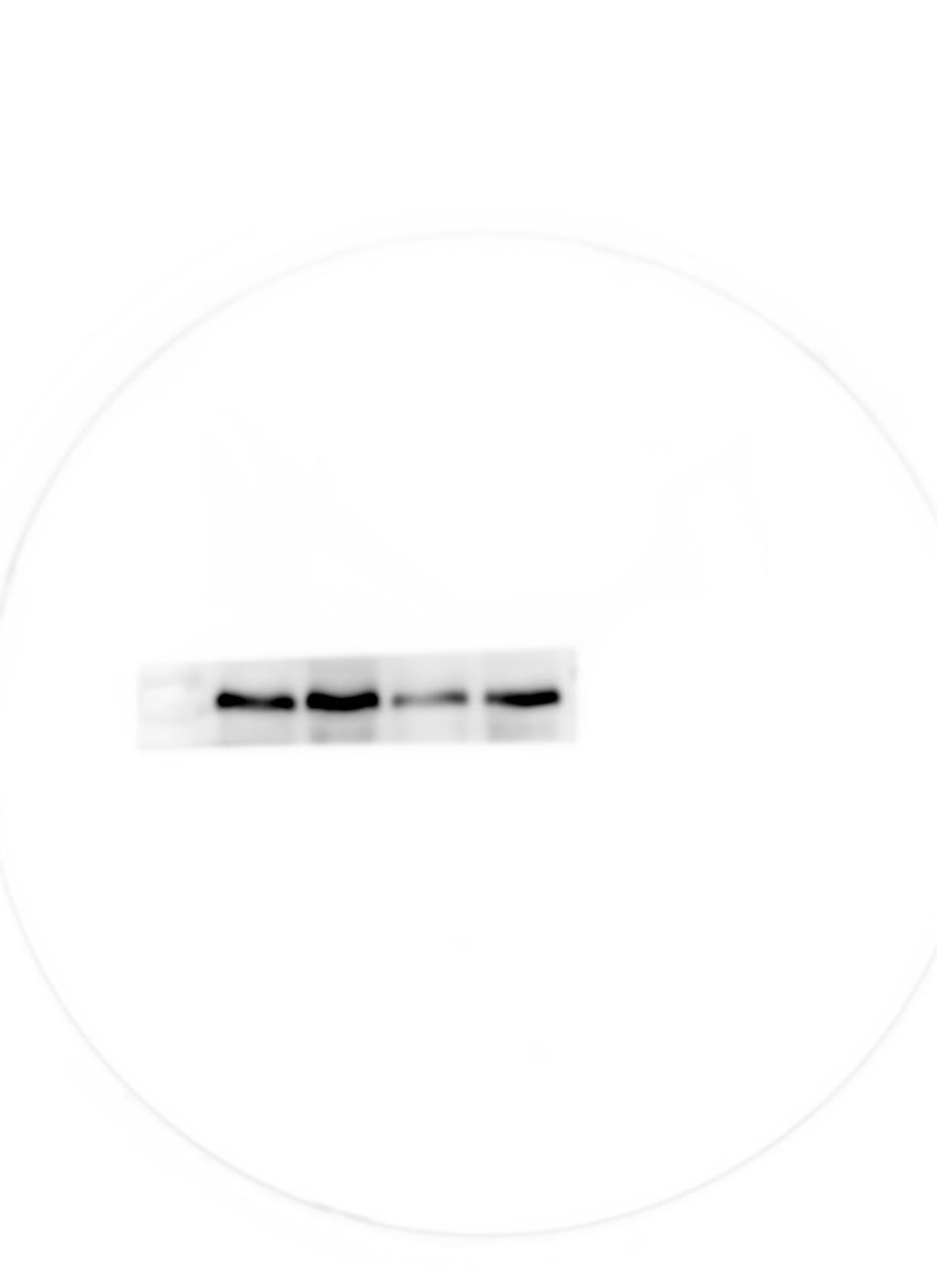

Supplement: Supplementary file 3 [file DataSheet_3.zip › raw original data-Fig4/WB/Fig4F-BECN1.jpg]

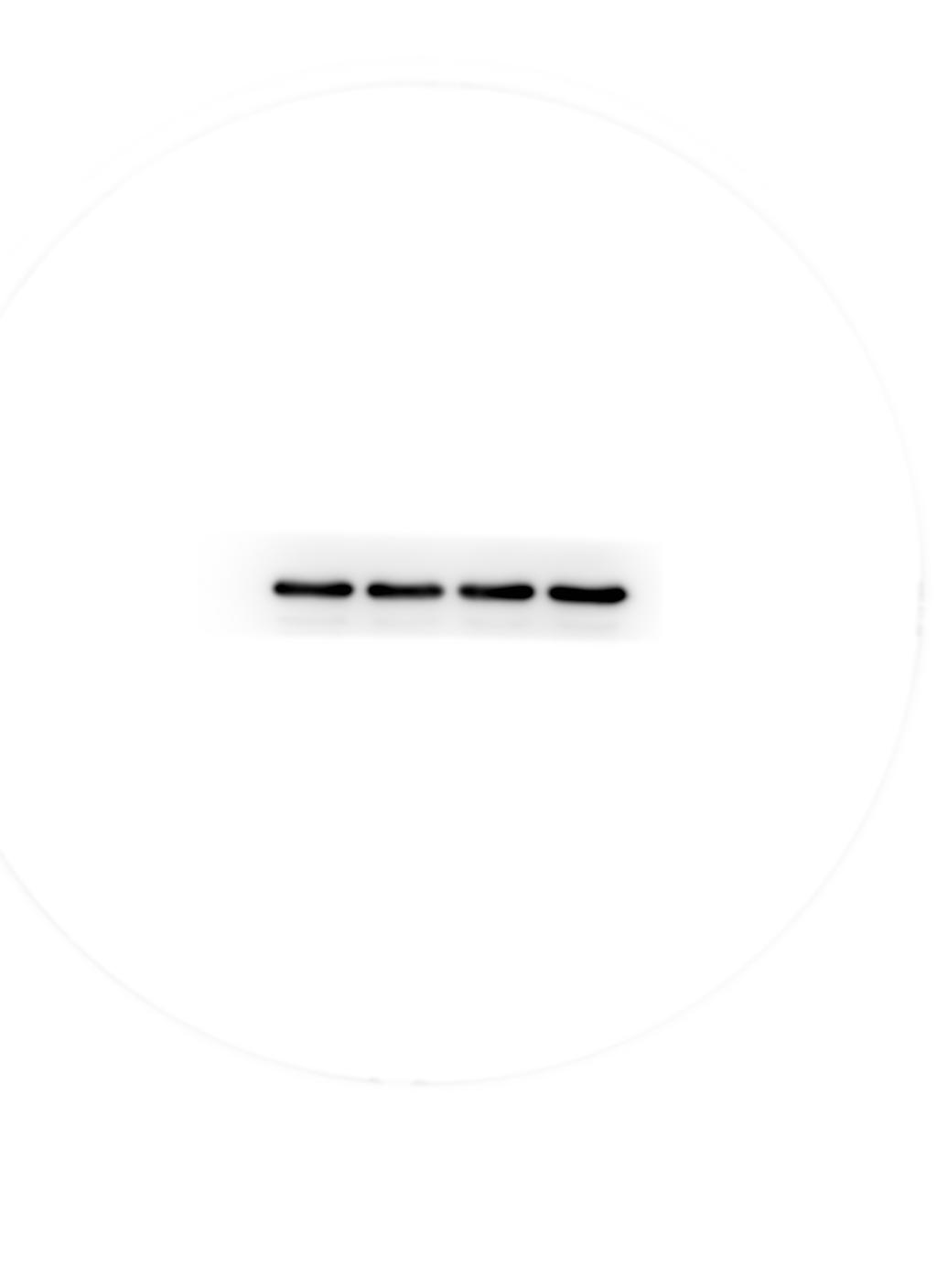

Supplement: Supplementary file 3 [file DataSheet_3.zip › raw original data-Fig4/WB/Fig4F-GAPDH.jpg]

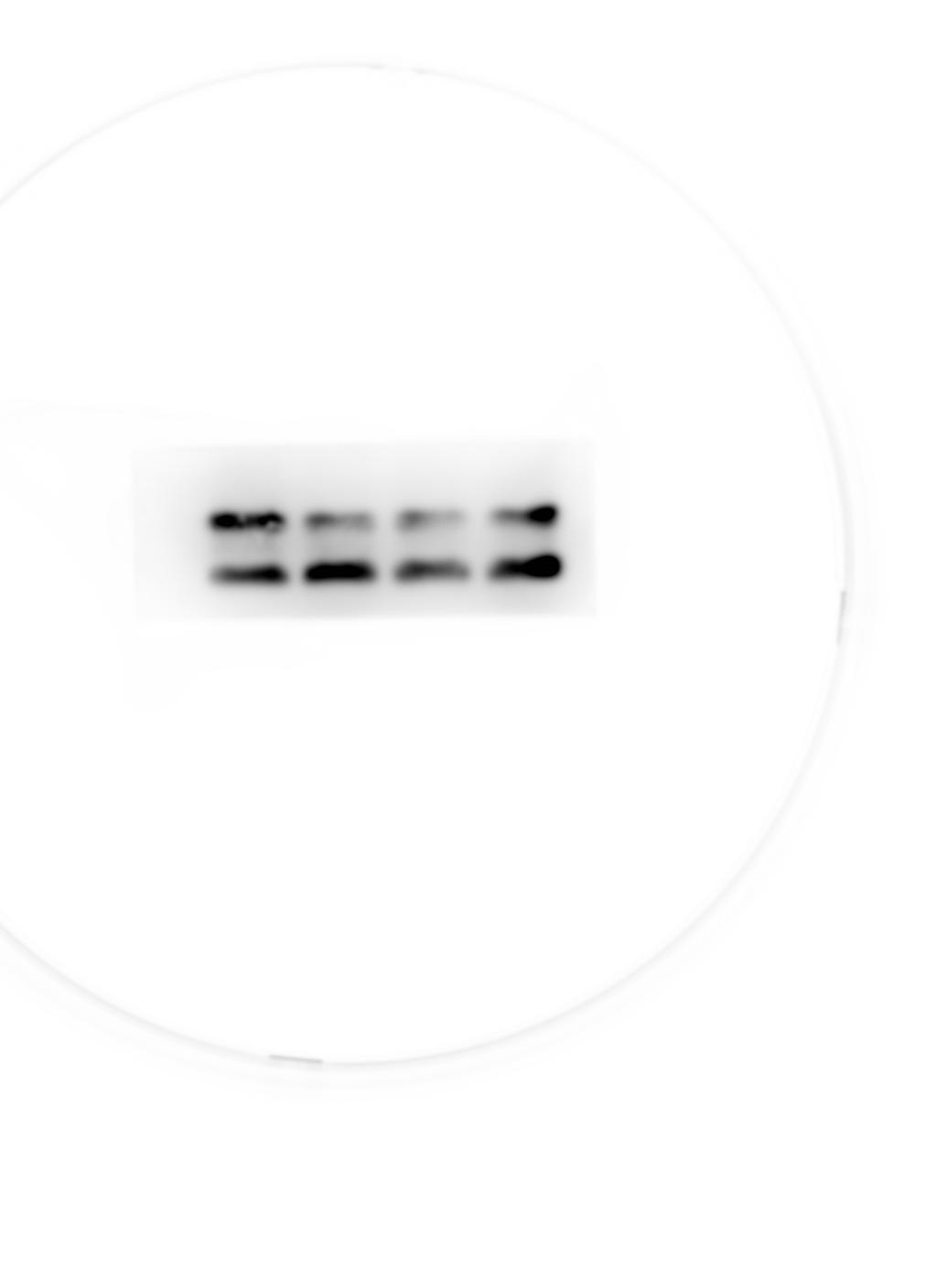

Supplement: Supplementary file 3 [file DataSheet_3.zip › raw original data-Fig4/WB/Fig4F-LC3B.jpg]

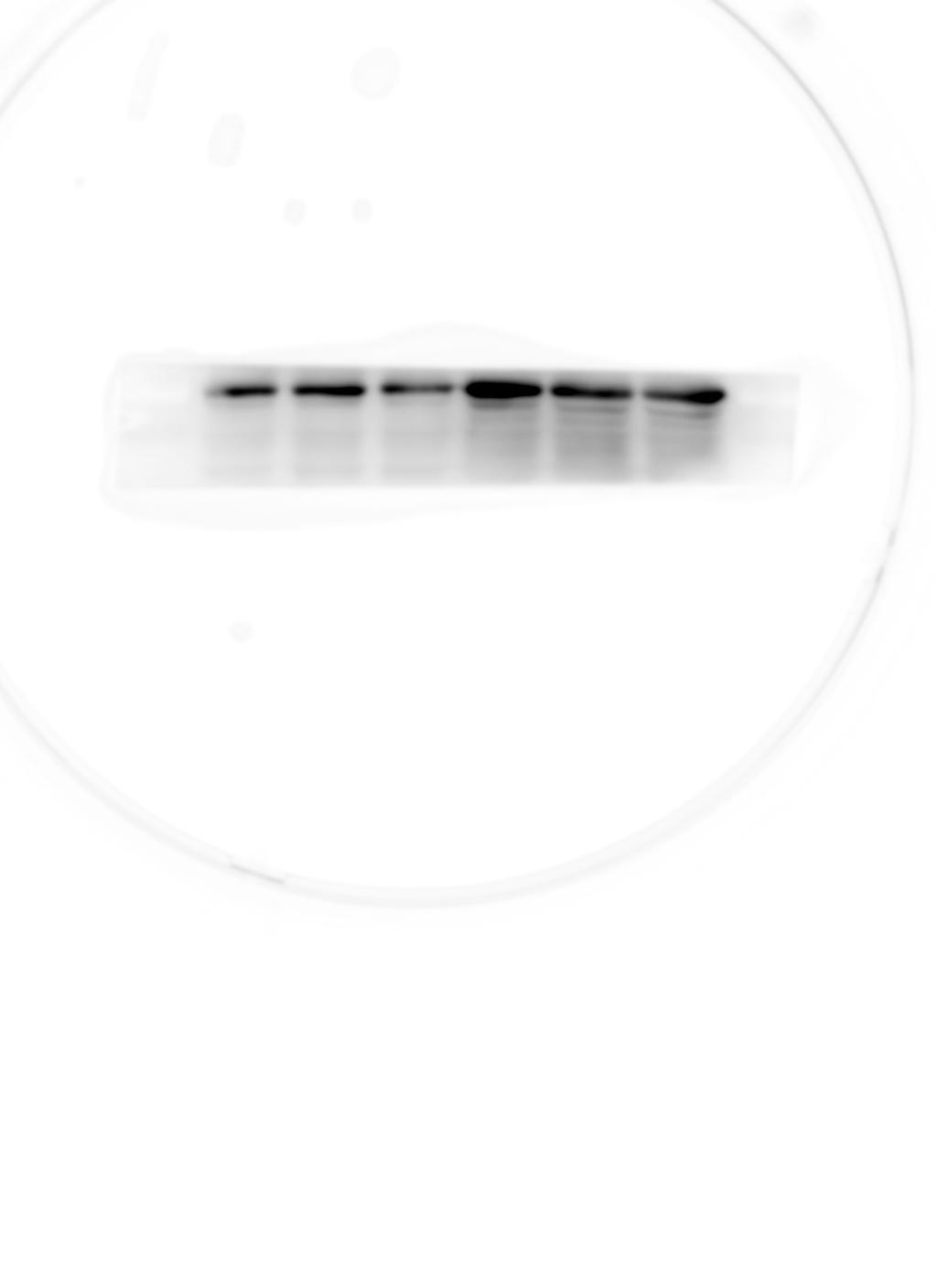

Supplement: Supplementary file 4 [file DataSheet_4.zip › raw original data-Fig5/WB/Fig5B-Aromatase.jpg]

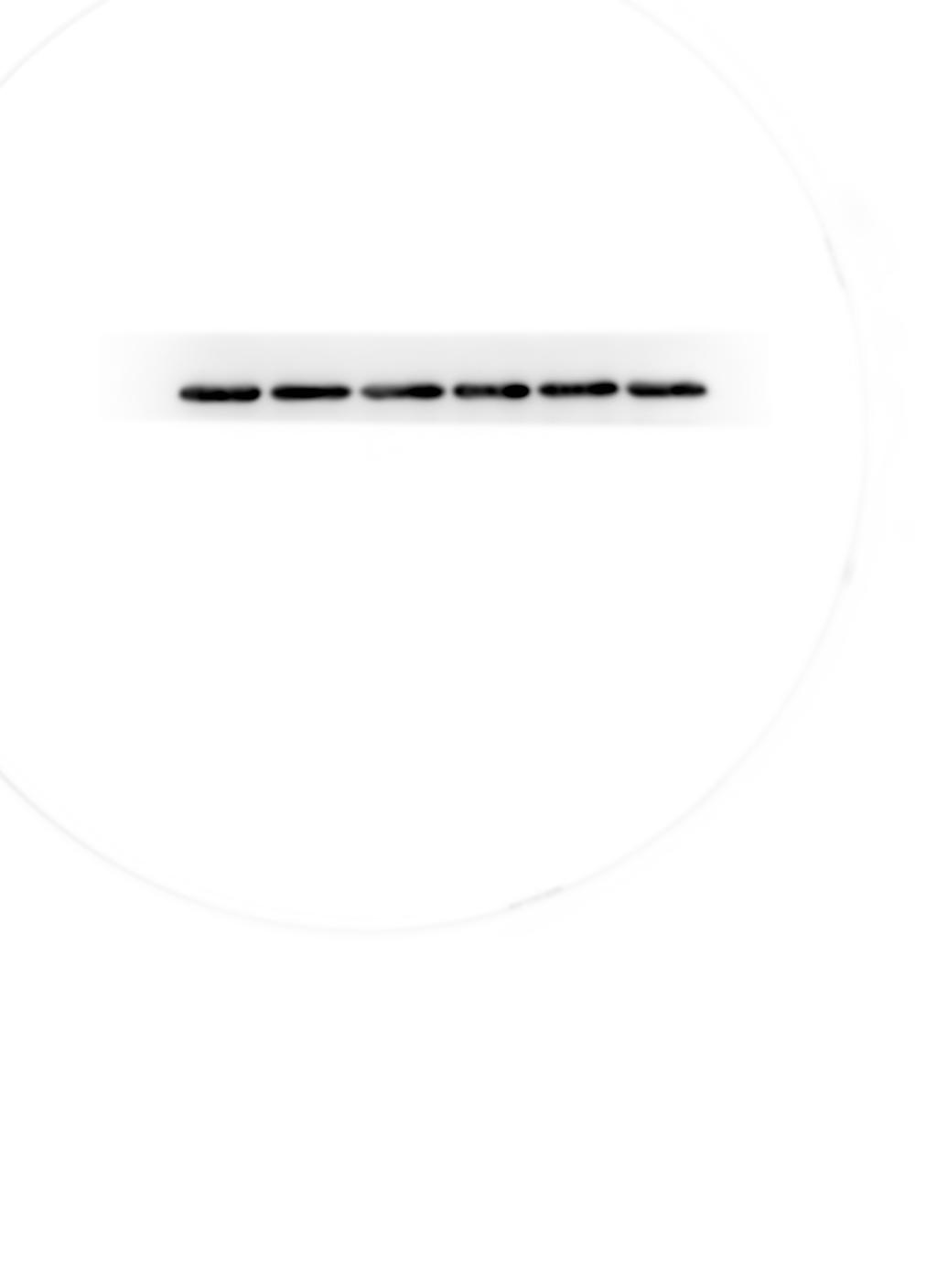

Supplement: Supplementary file 4 [file DataSheet_4.zip › raw original data-Fig5/WB/Fig5B-GAPDH.jpg]

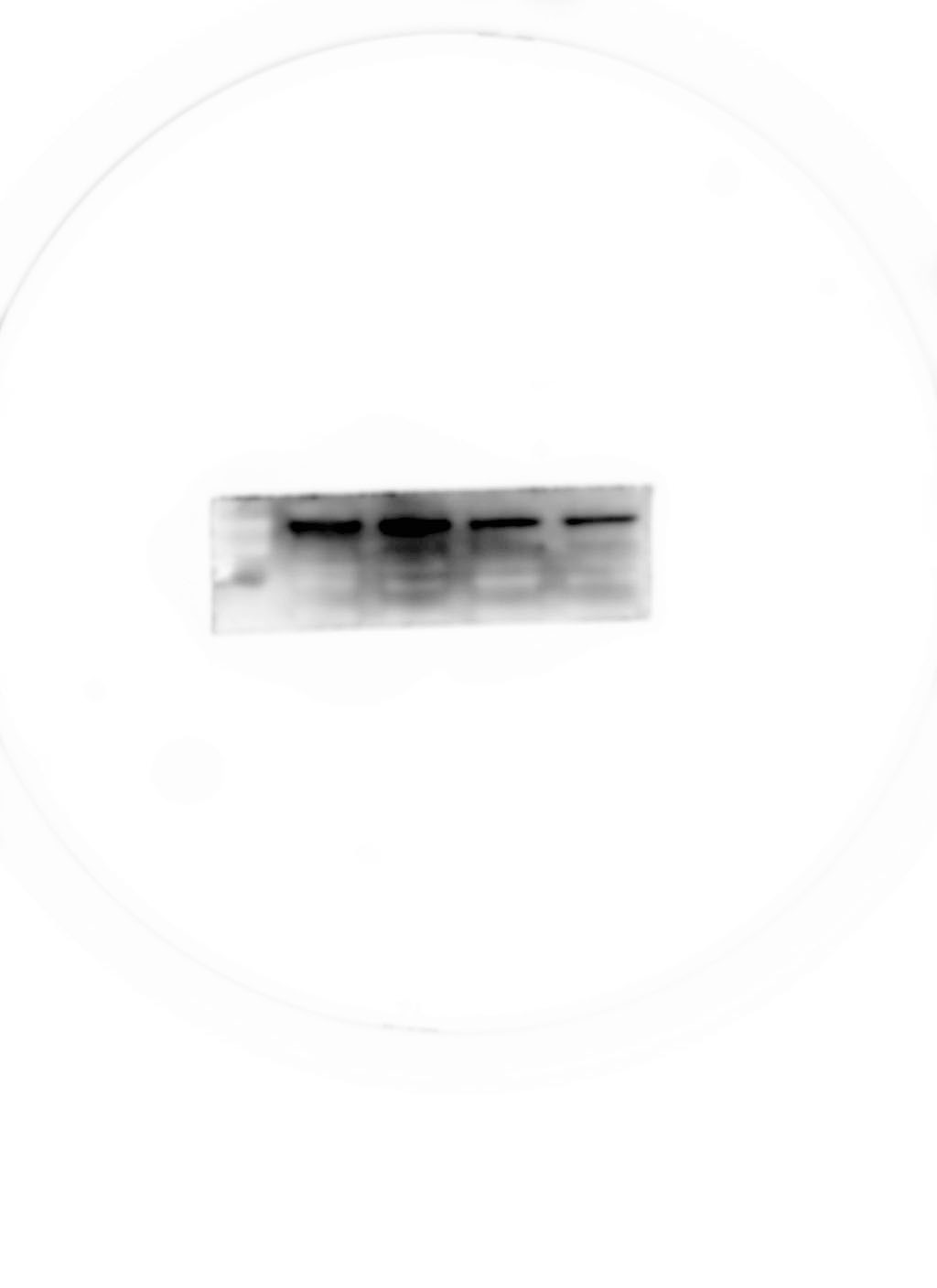

Supplement: Supplementary file 4 [file DataSheet_4.zip › raw original data-Fig5/WB/Fig5C-Aromatase.jpg]

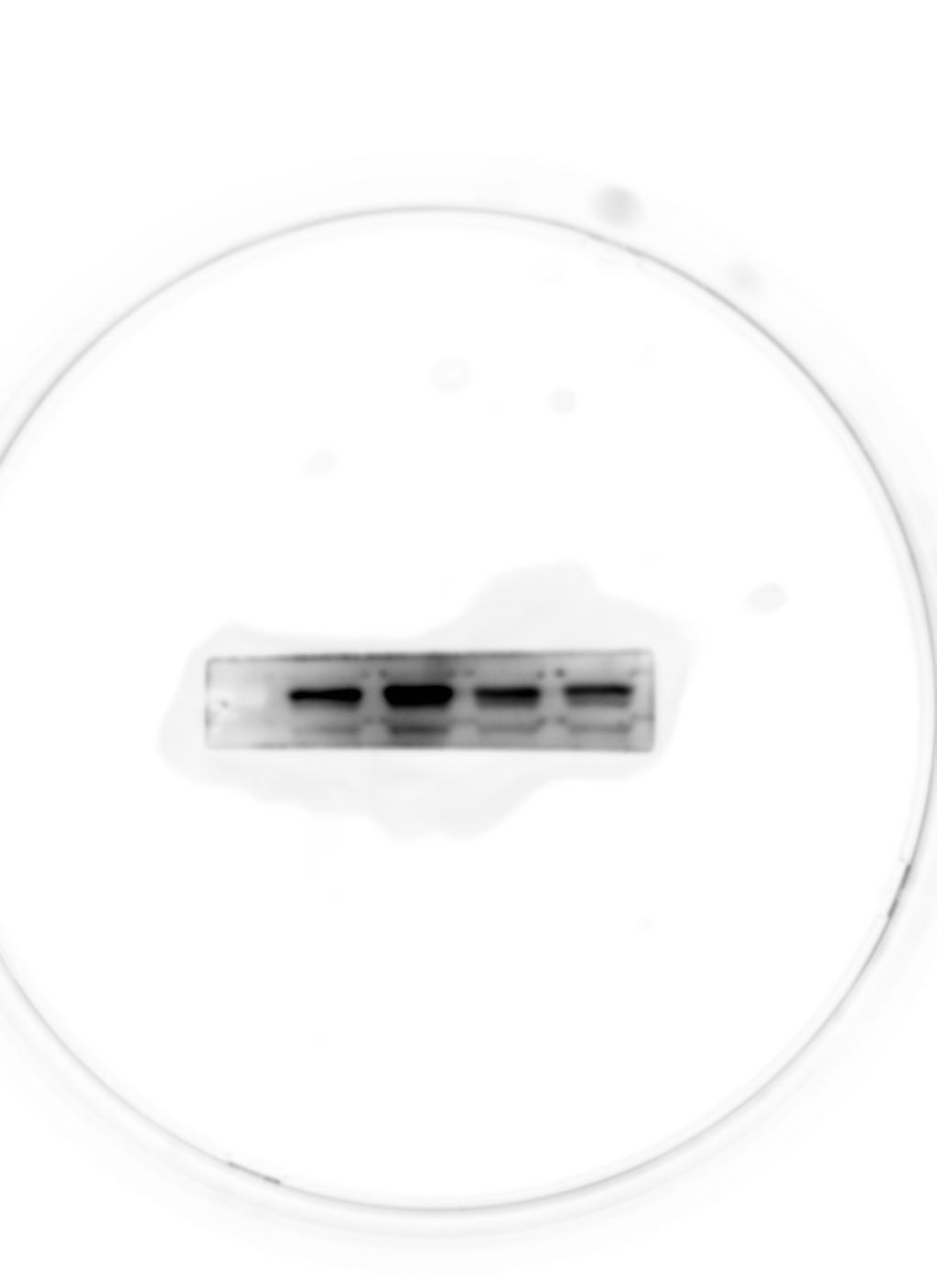

Supplement: Supplementary file 4 [file DataSheet_4.zip › raw original data-Fig5/WB/Fig5C-ERa.jpg]

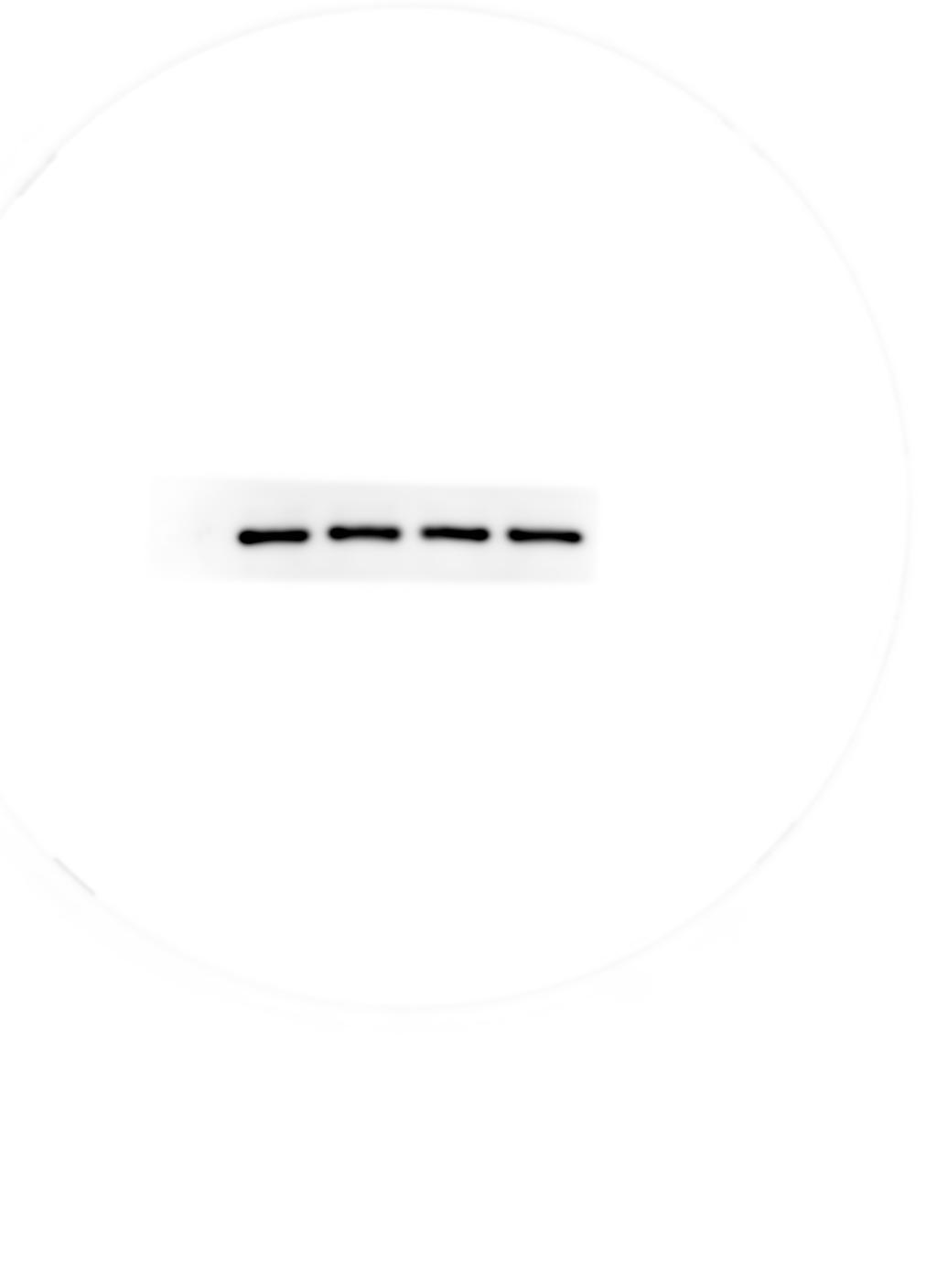

Supplement: Supplementary file 4 [file DataSheet_4.zip › raw original data-Fig5/WB/Fig5C-GAPDH.jpg]

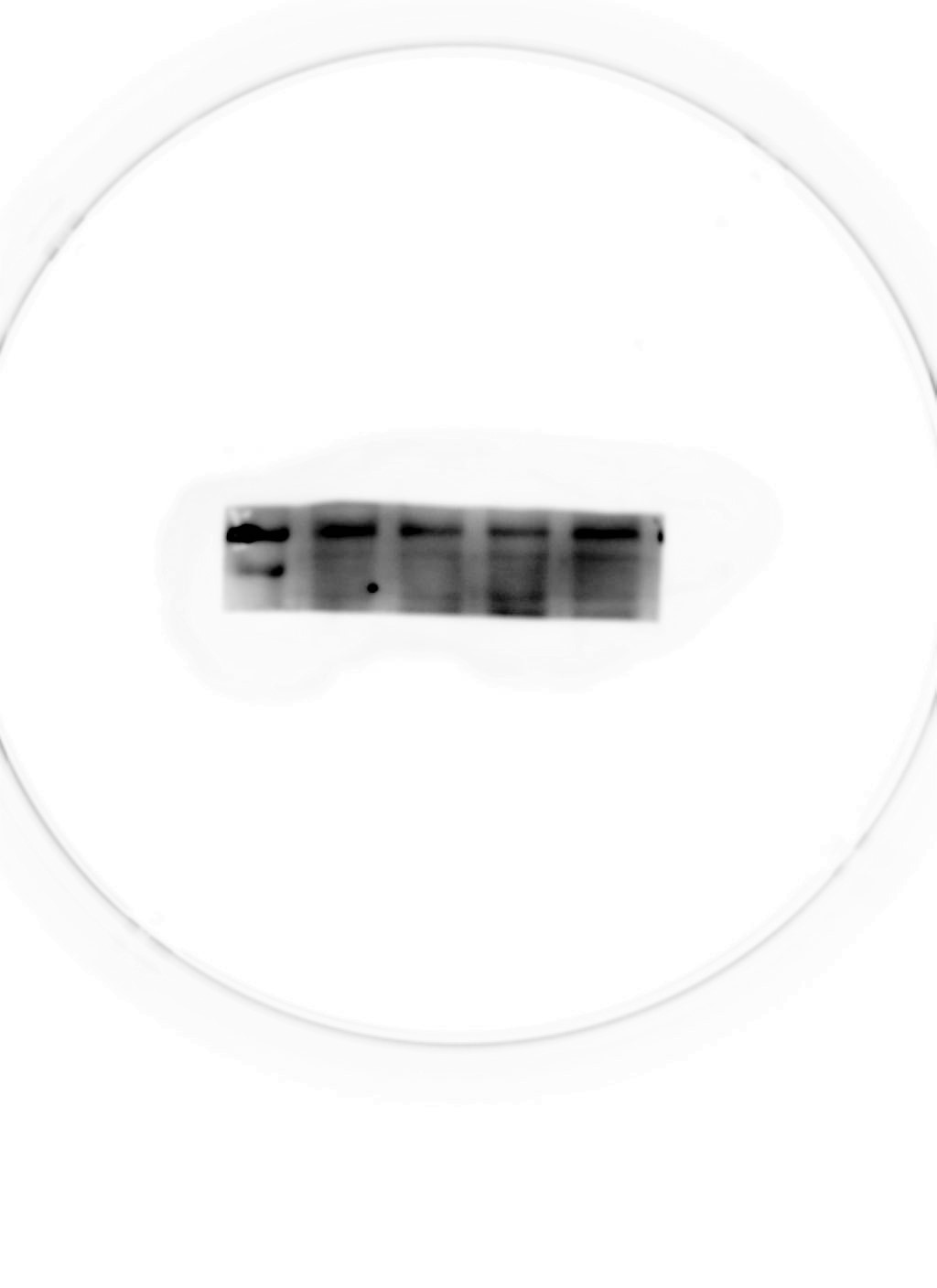

Supplement: Supplementary file 4 [file DataSheet_4.zip › raw original data-Fig5/WB/Fig5E-Aromatase.jpg]

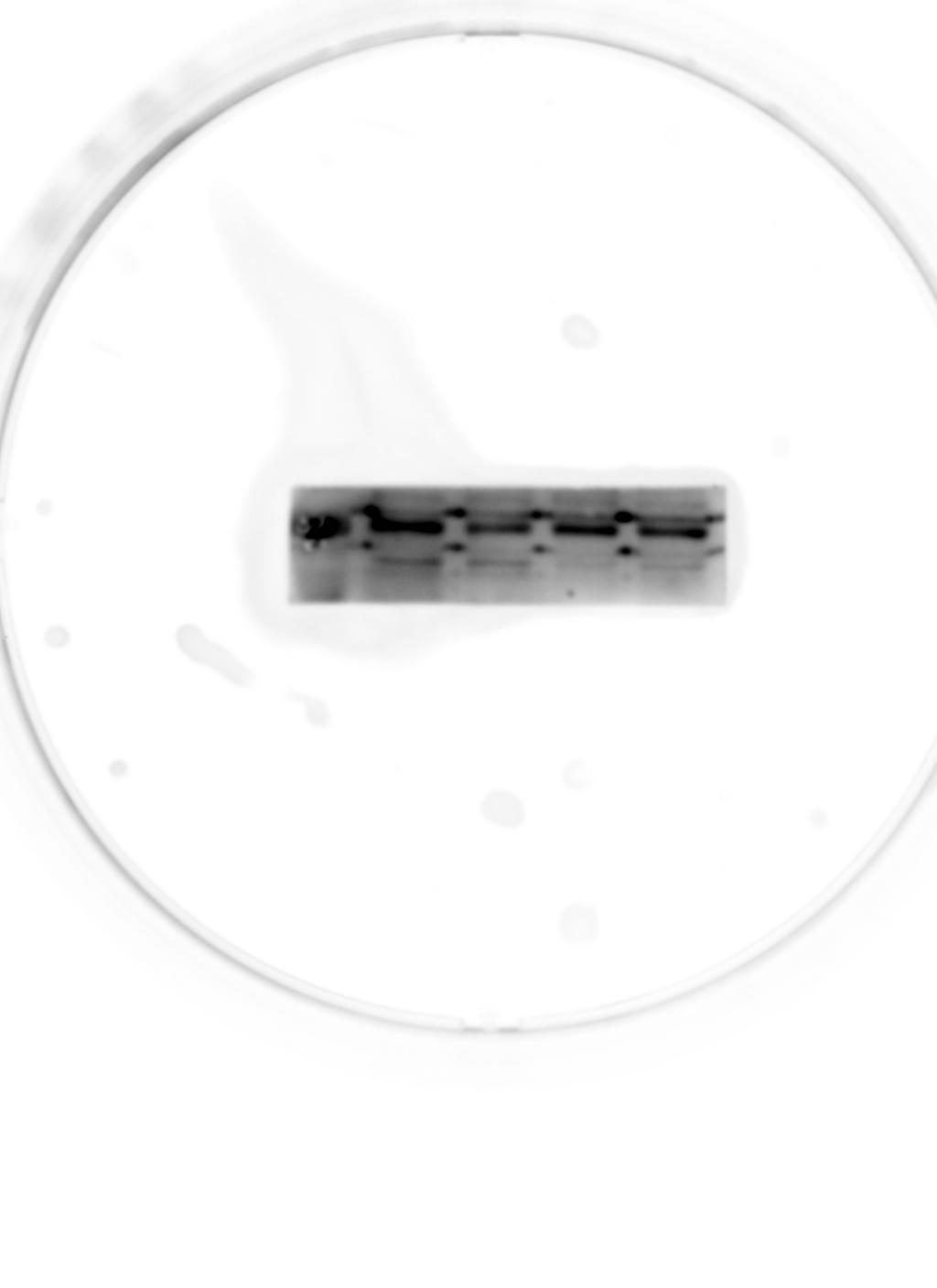

Supplement: Supplementary file 4 [file DataSheet_4.zip › raw original data-Fig5/WB/Fig5E-ERa.jpg]

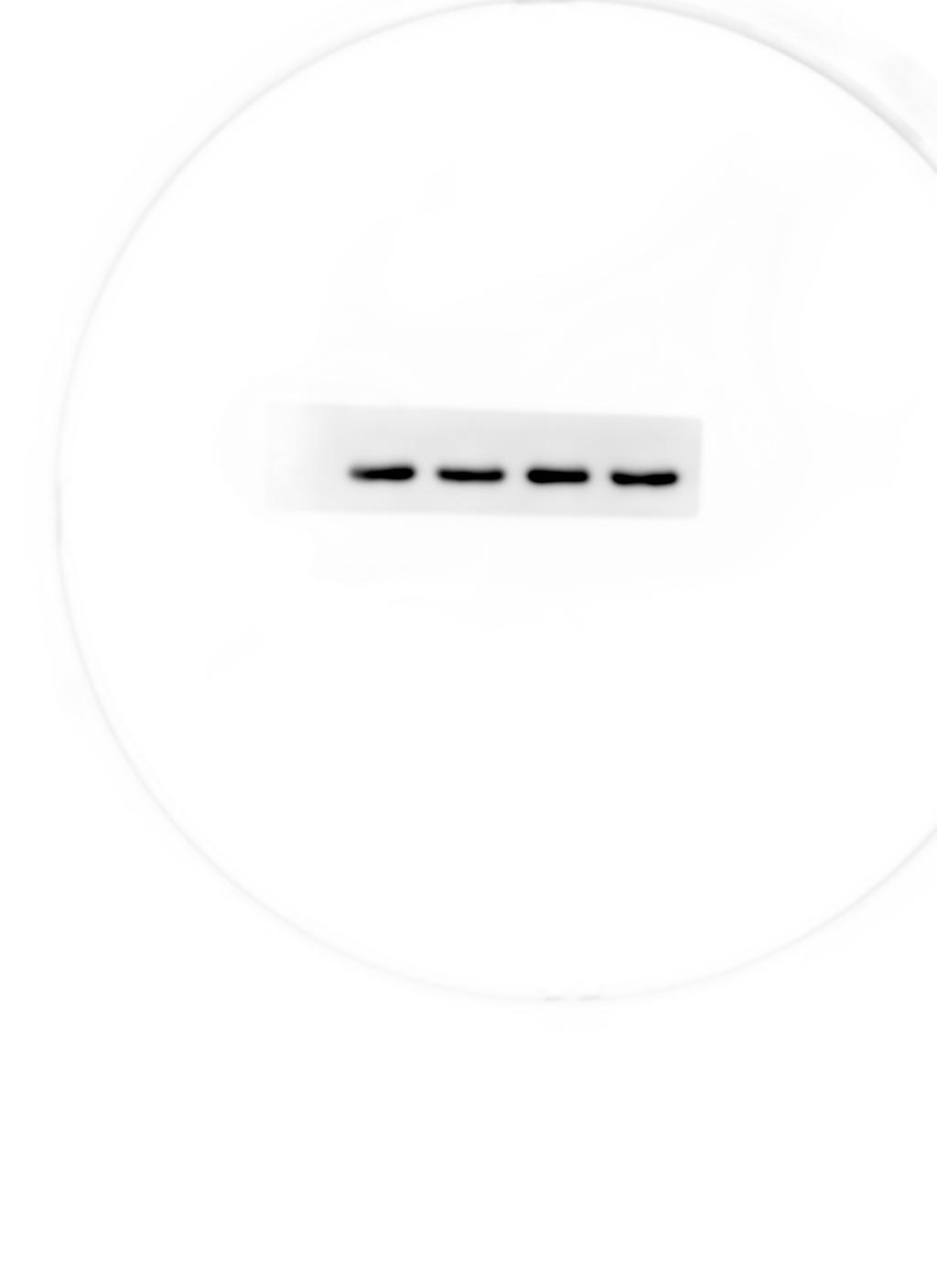

Supplement: Supplementary file 4 [file DataSheet_4.zip › raw original data-Fig5/WB/Fig5E-GAPDH.jpg]

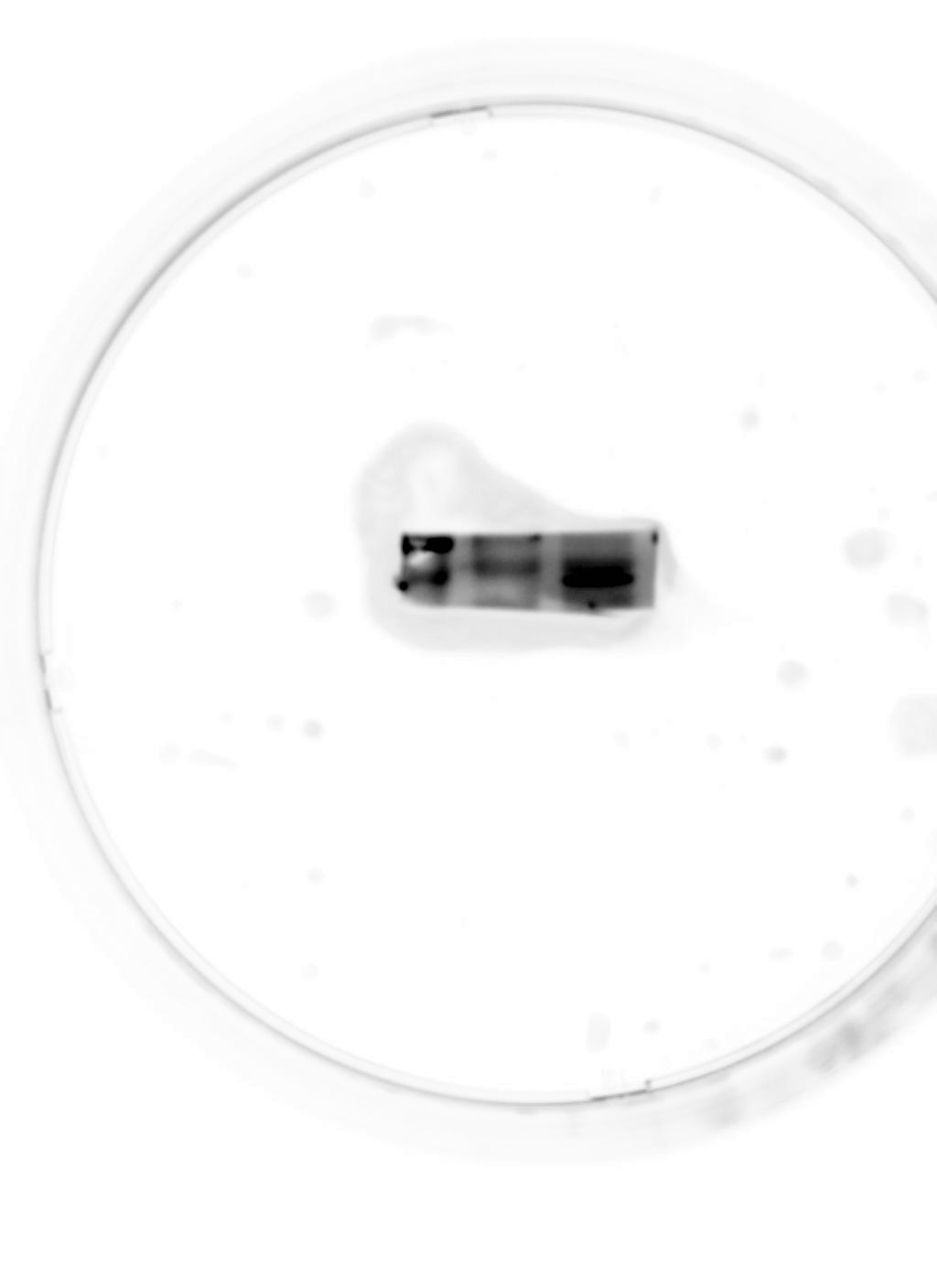

Supplement: Supplementary file 4 [file DataSheet_4.zip › raw original data-Fig5/WB/Fig5G-Aromatase.jpg]

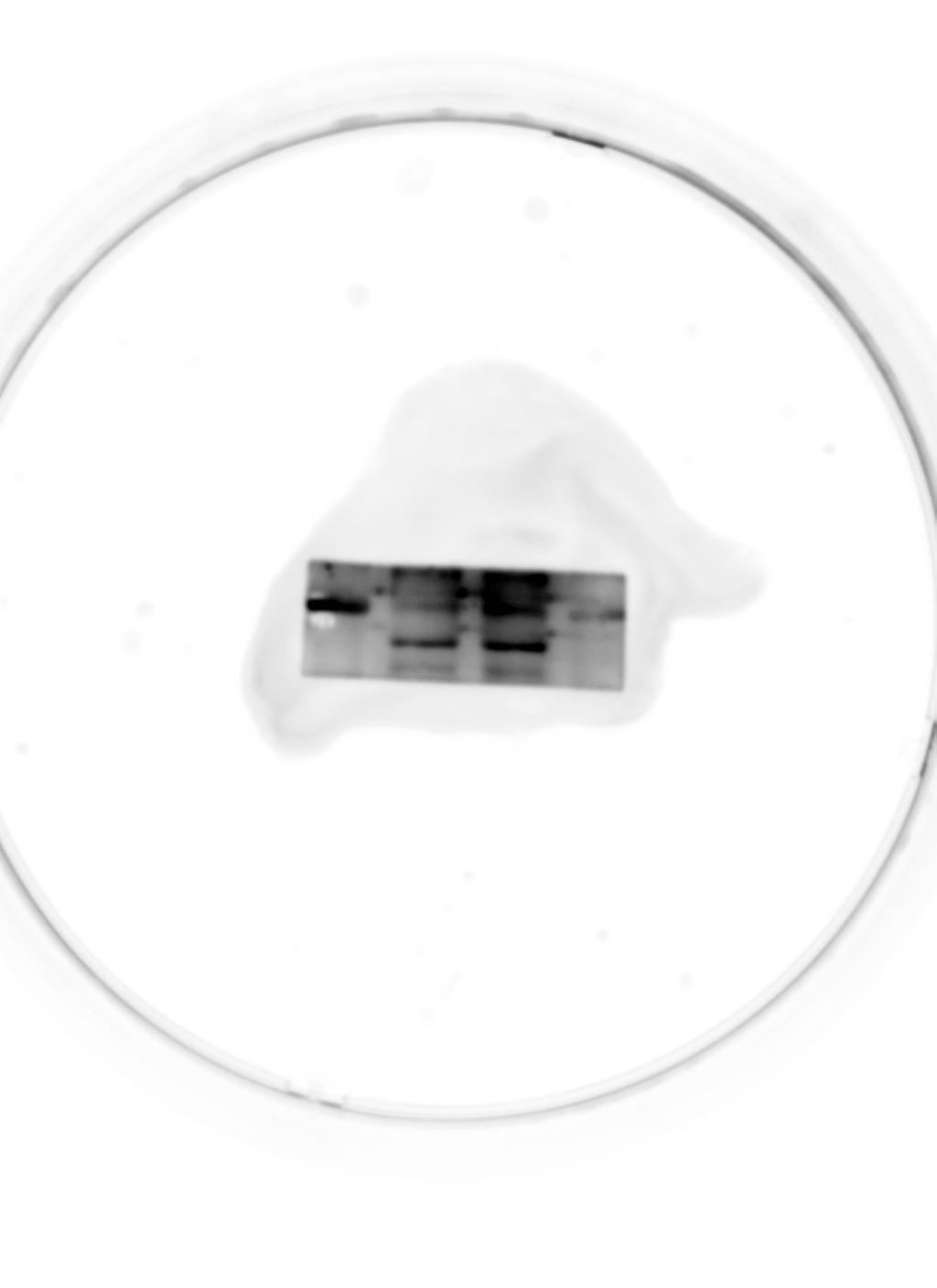

Supplement: Supplementary file 4 [file DataSheet_4.zip › raw original data-Fig5/WB/Fig5G-ERa.jpg]

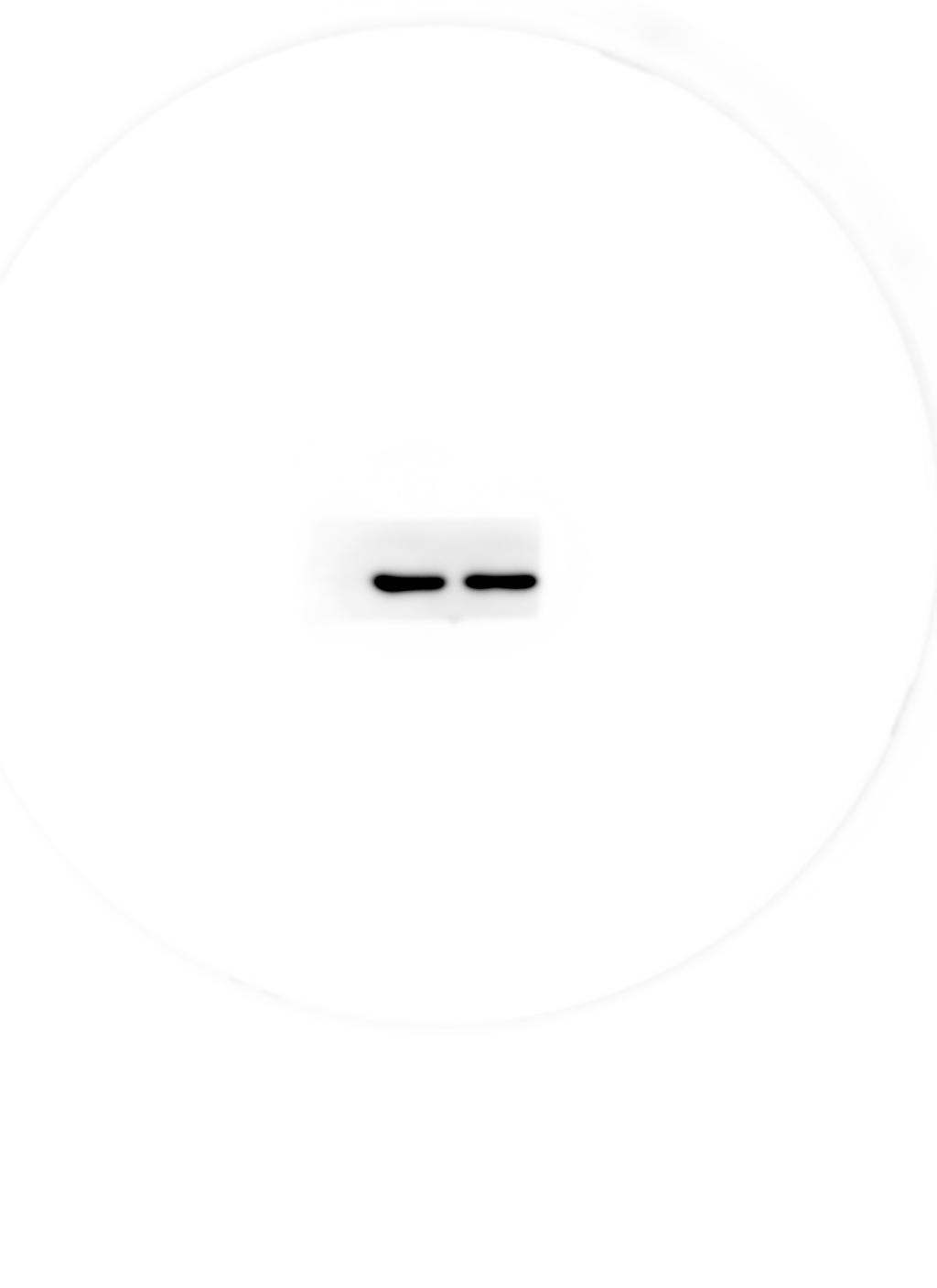

Supplement: Supplementary file 4 [file DataSheet_4.zip › raw original data-Fig5/WB/Fig5G-GAPDH.jpg]

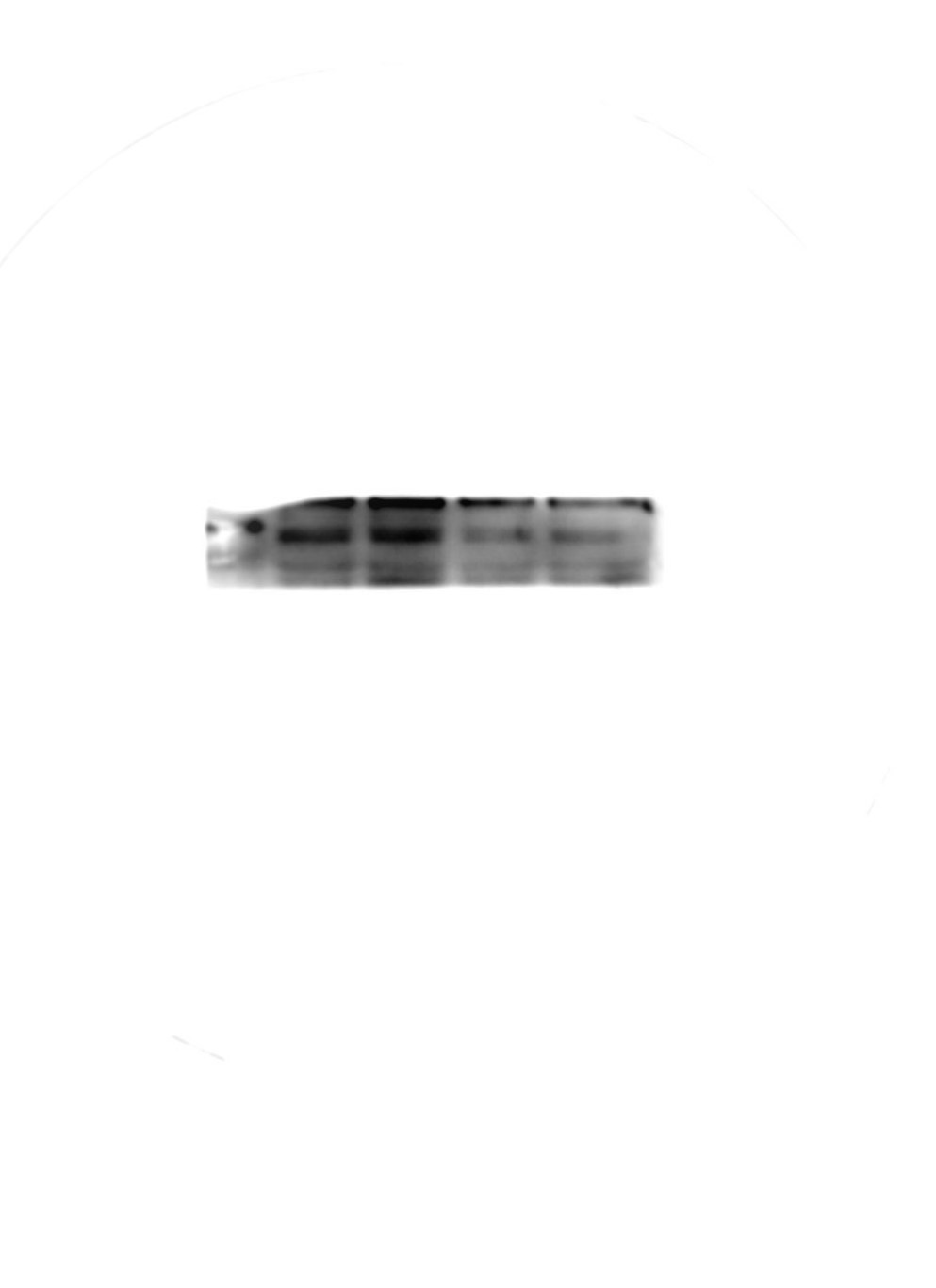

Supplement: Supplementary file 4 [file DataSheet_4.zip › raw original data-Fig5/WB/Fig5I-Aromatase.jpg]

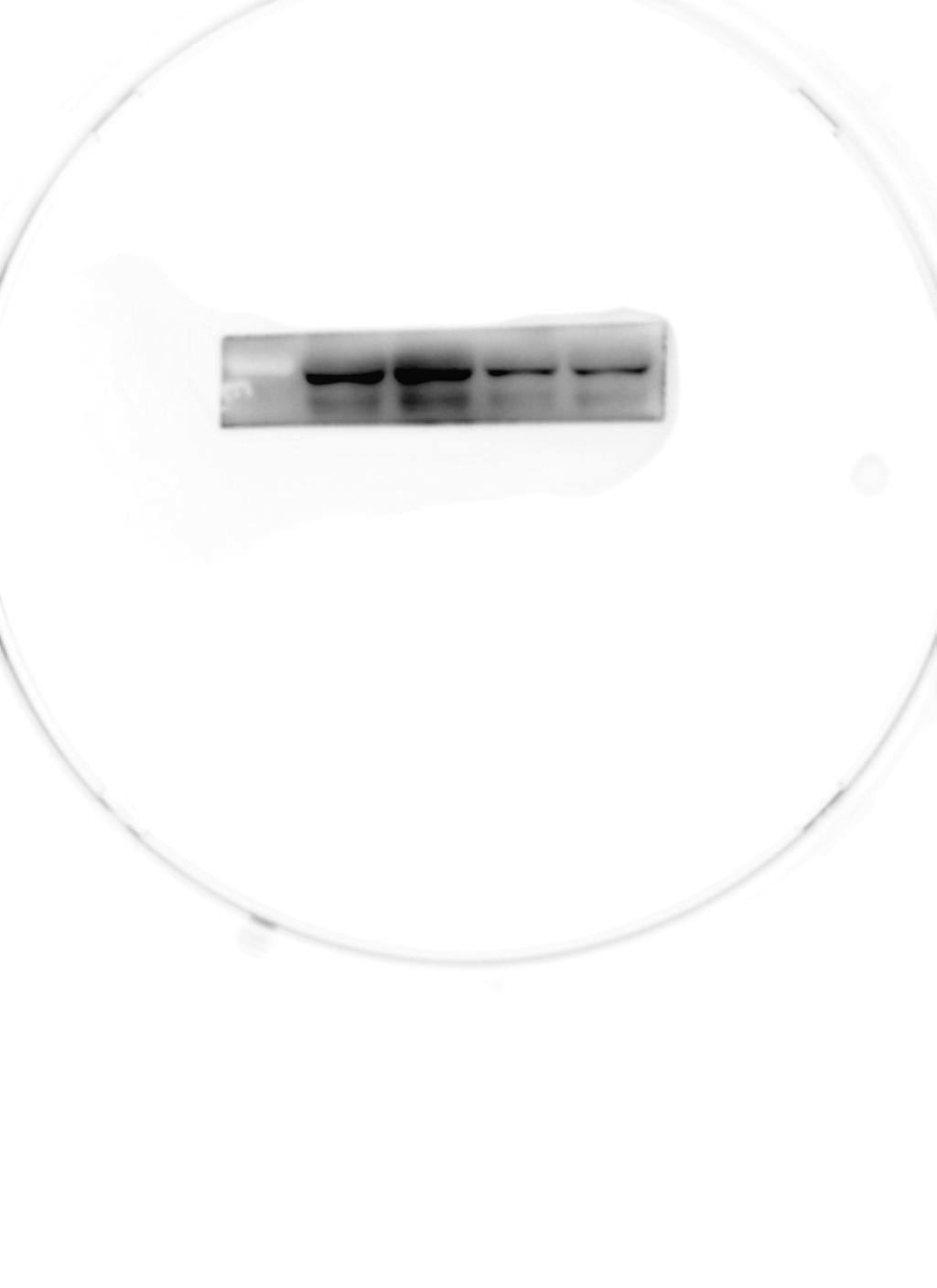

Supplement: Supplementary file 4 [file DataSheet_4.zip › raw original data-Fig5/WB/Fig5I-ERa.jpg]

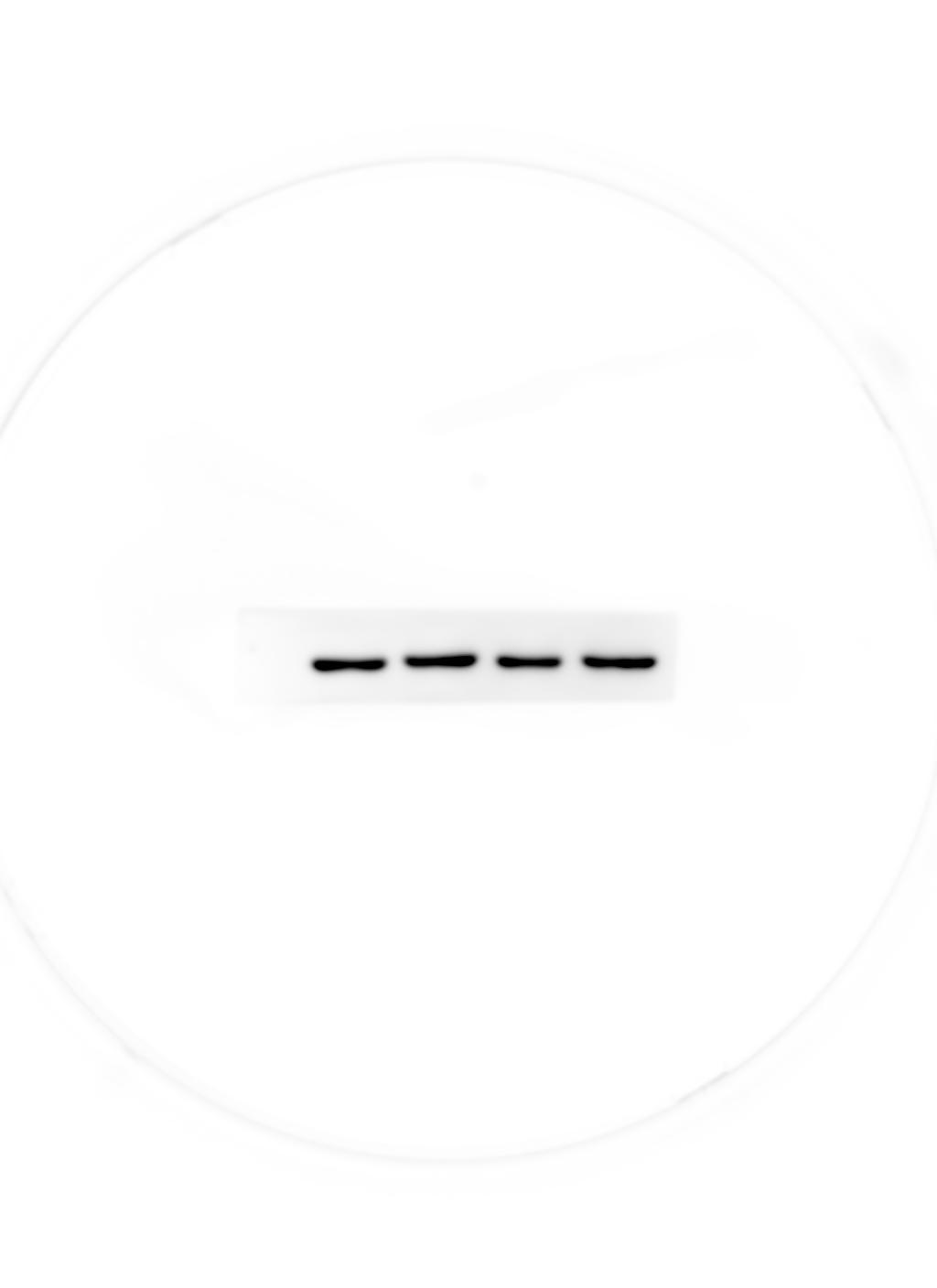

Supplement: Supplementary file 4 [file DataSheet_4.zip › raw original data-Fig5/WB/Fig5I-GAPDH.jpg]

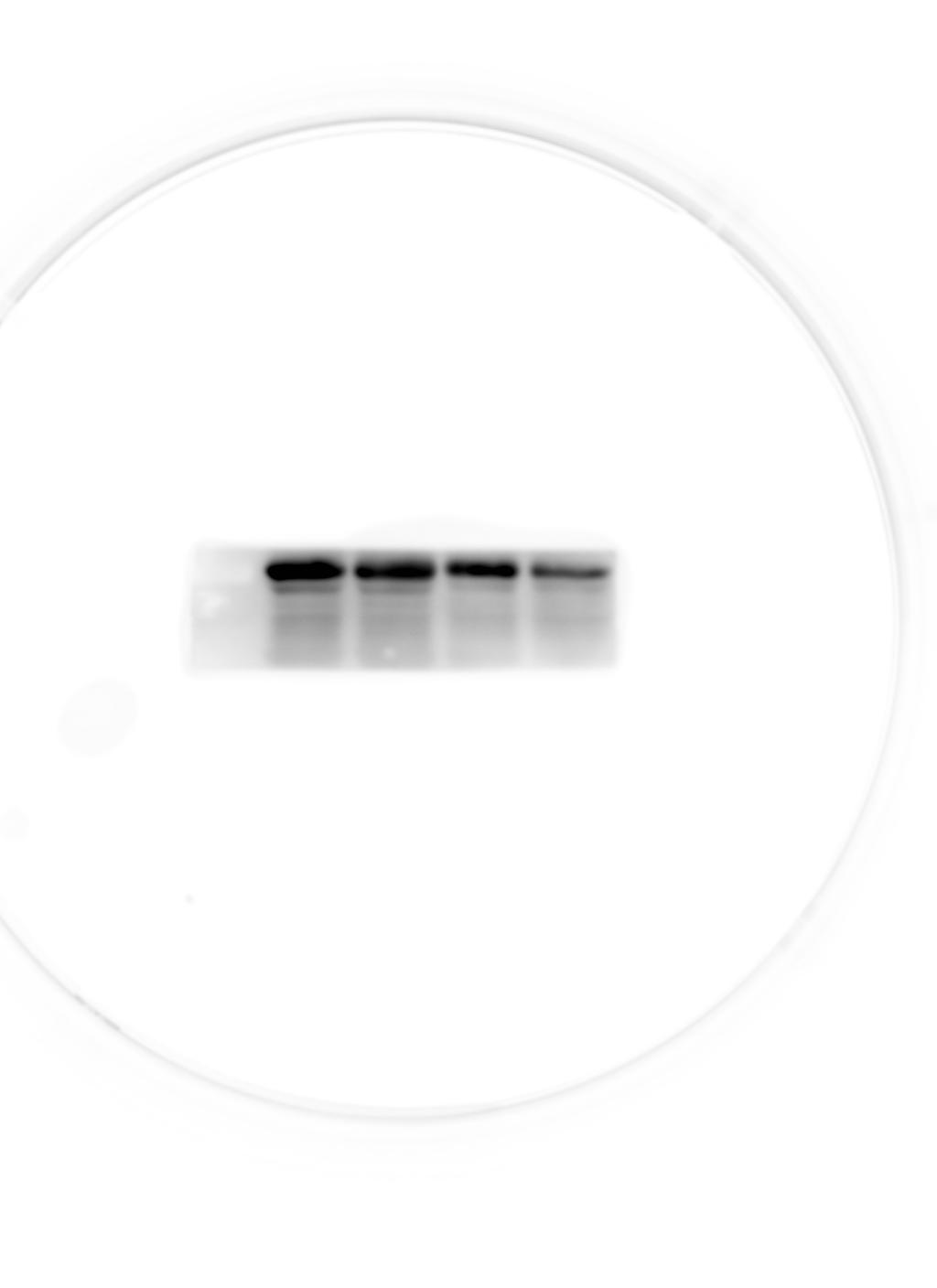

Supplement: Supplementary file 5 [file DataSheet_5.zip › raw original data-Fig6/WB/Fig6B-Aromatase.jpg]

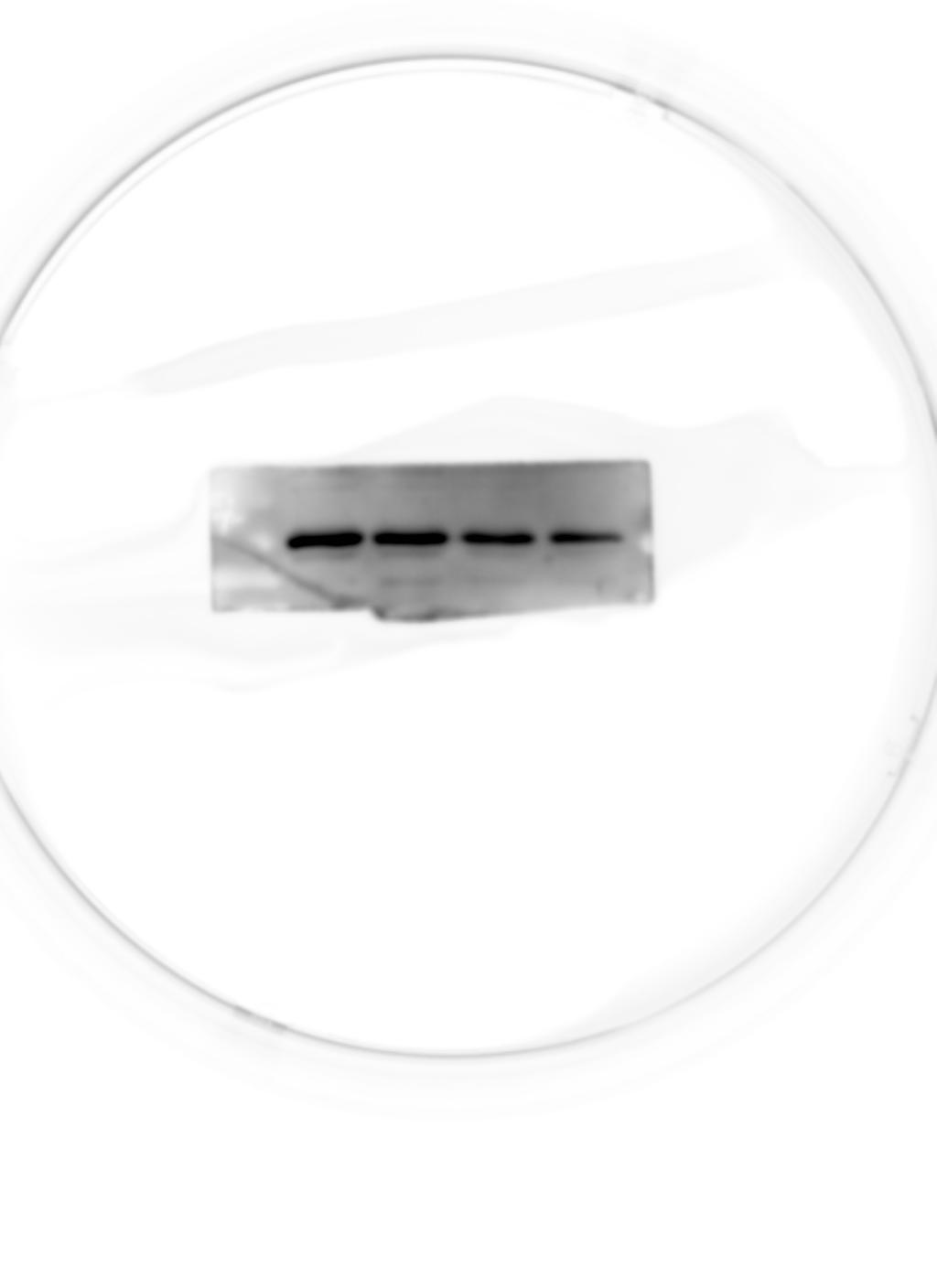

Supplement: Supplementary file 5 [file DataSheet_5.zip › raw original data-Fig6/WB/Fig6B-ERa.jpg]

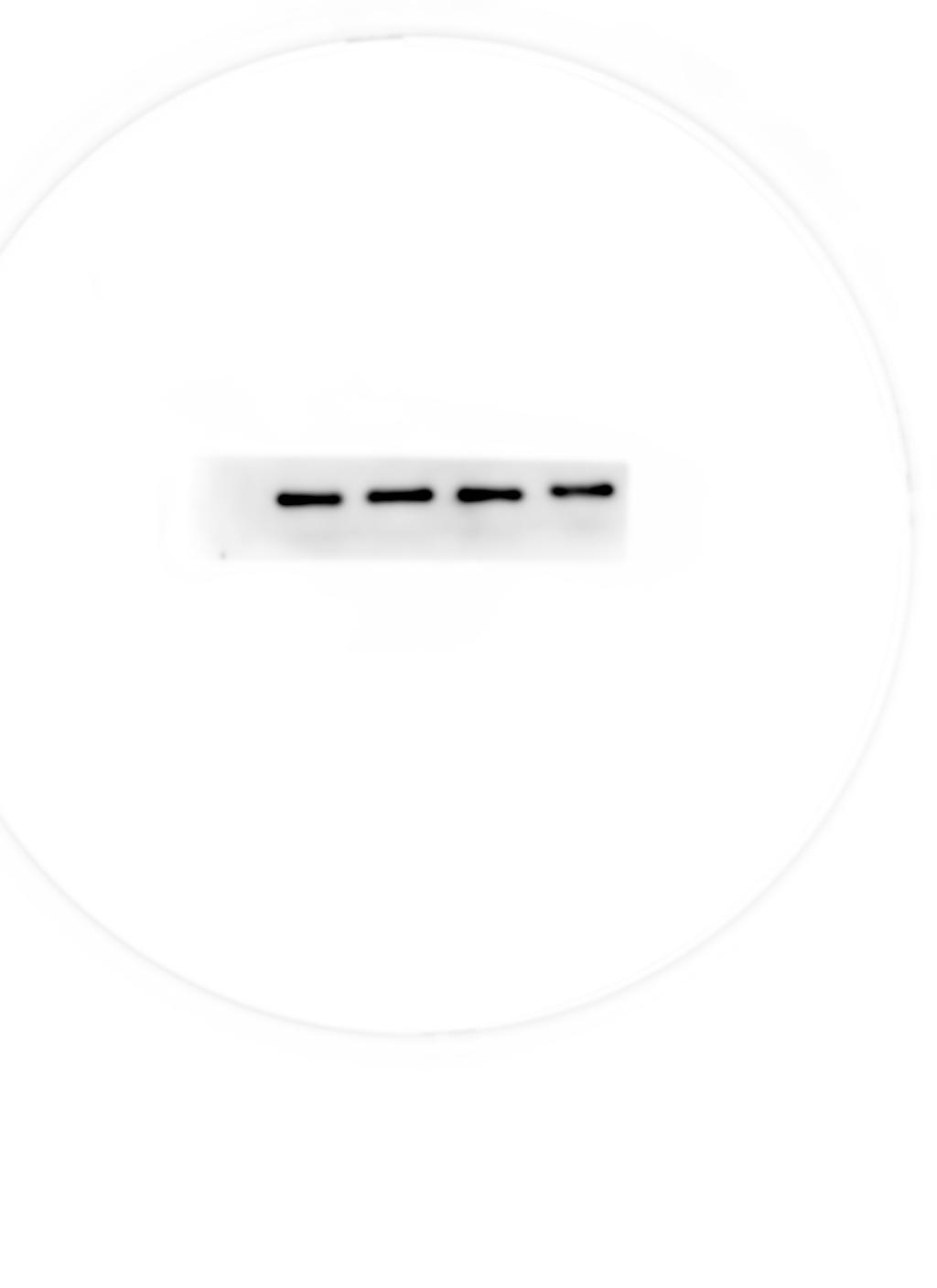

Supplement: Supplementary file 5 [file DataSheet_5.zip › raw original data-Fig6/WB/Fig6B-GAPDH.jpg]

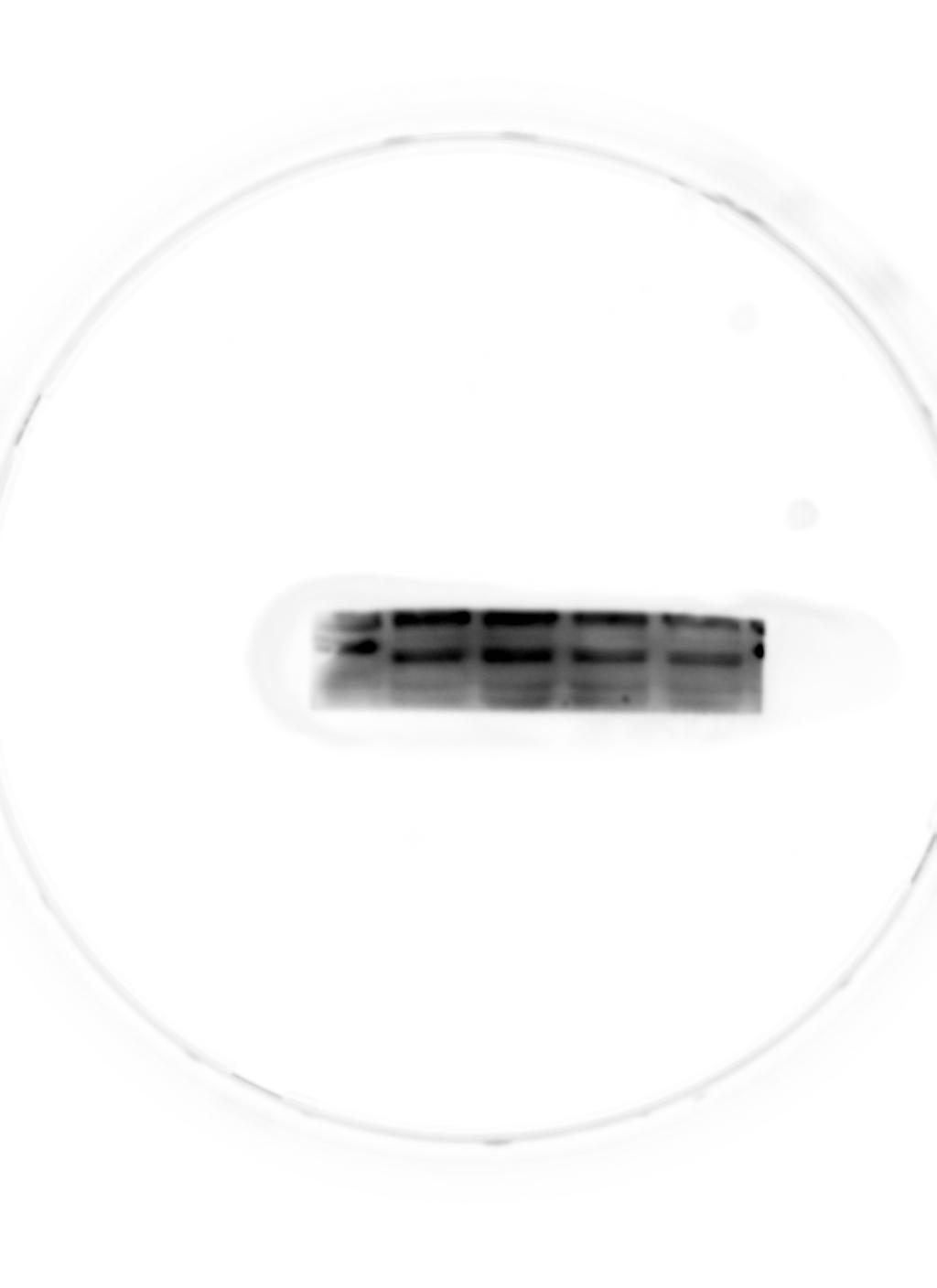

Supplement: Supplementary file 5 [file DataSheet_5.zip › raw original data-Fig6/WB/Fig6C-Aromatase.jpg]

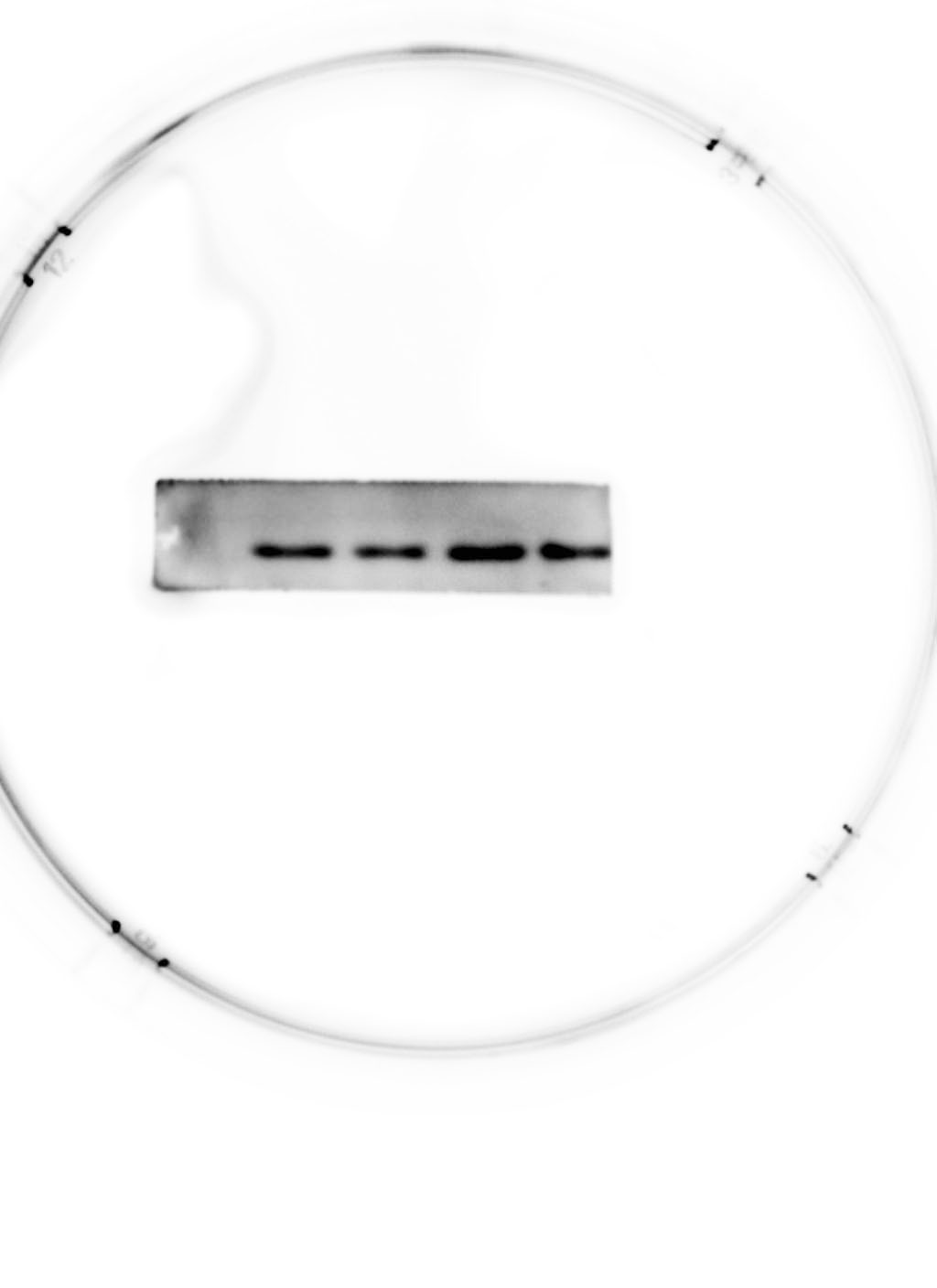

Supplement: Supplementary file 5 [file DataSheet_5.zip › raw original data-Fig6/WB/Fig6C-Bax.jpg]

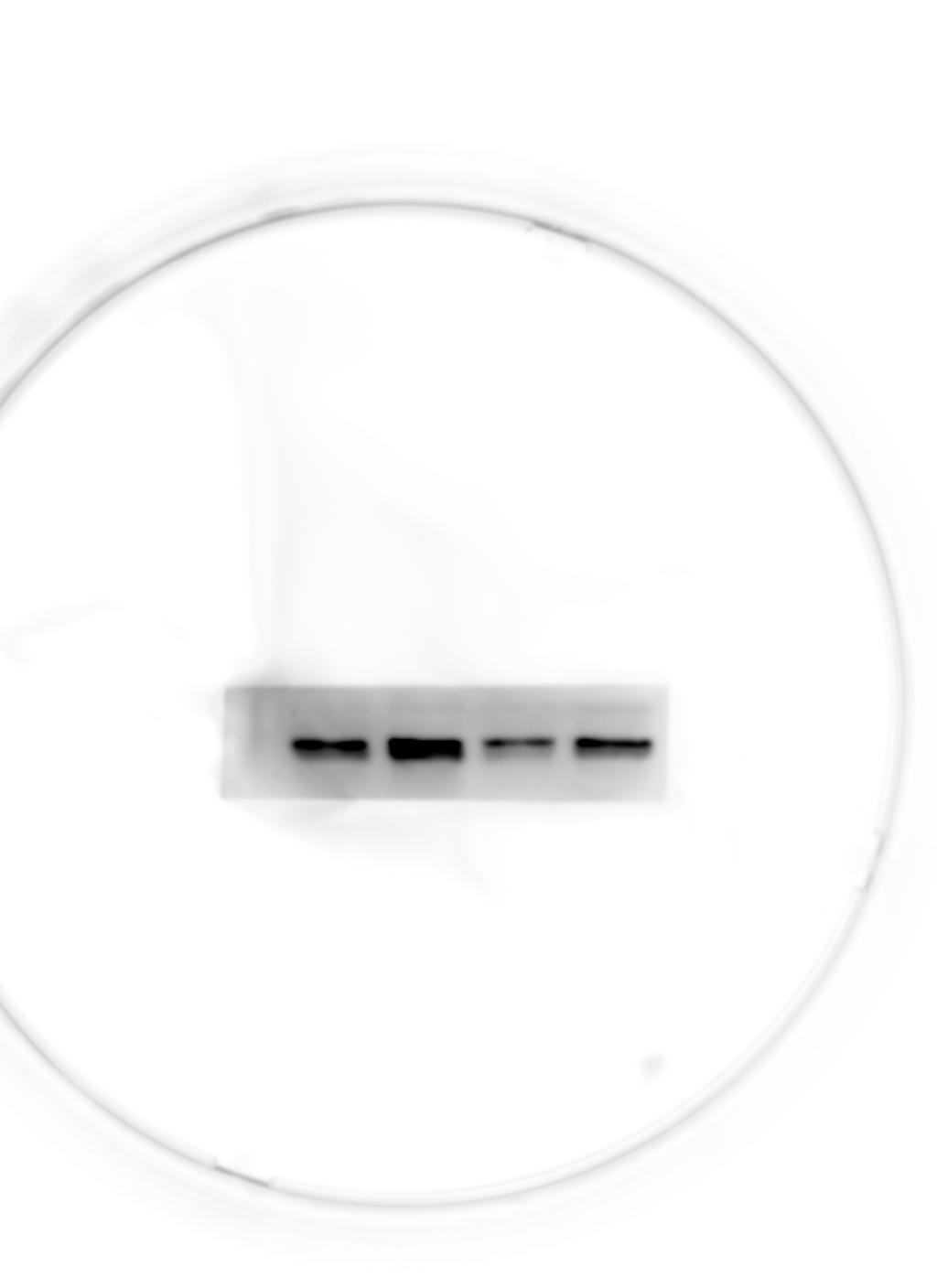

Supplement: Supplementary file 5 [file DataSheet_5.zip › raw original data-Fig6/WB/Fig6C-Bcl2.jpg]

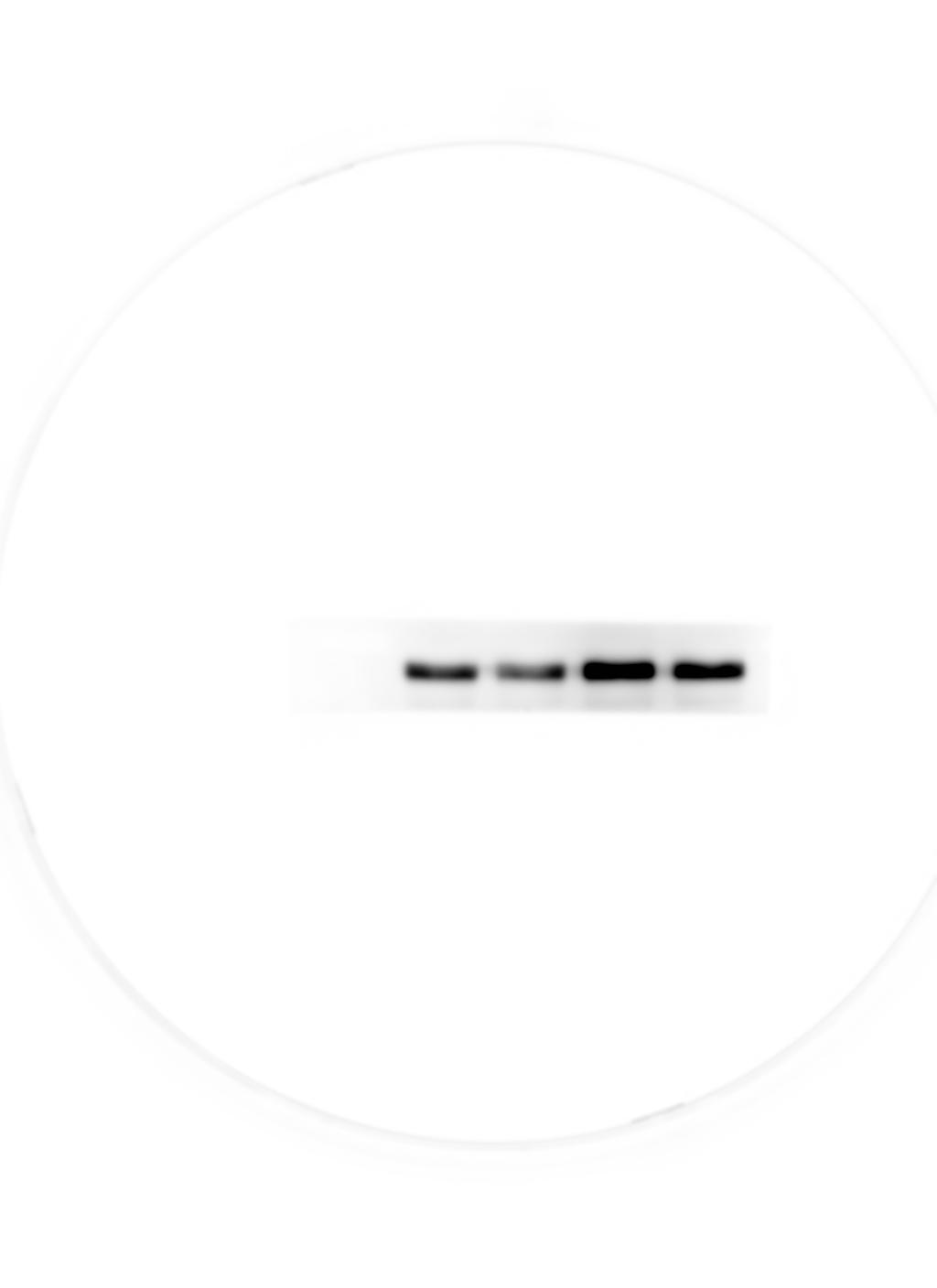

Supplement: Supplementary file 5 [file DataSheet_5.zip › raw original data-Fig6/WB/Fig6C-BECN1.jpg]

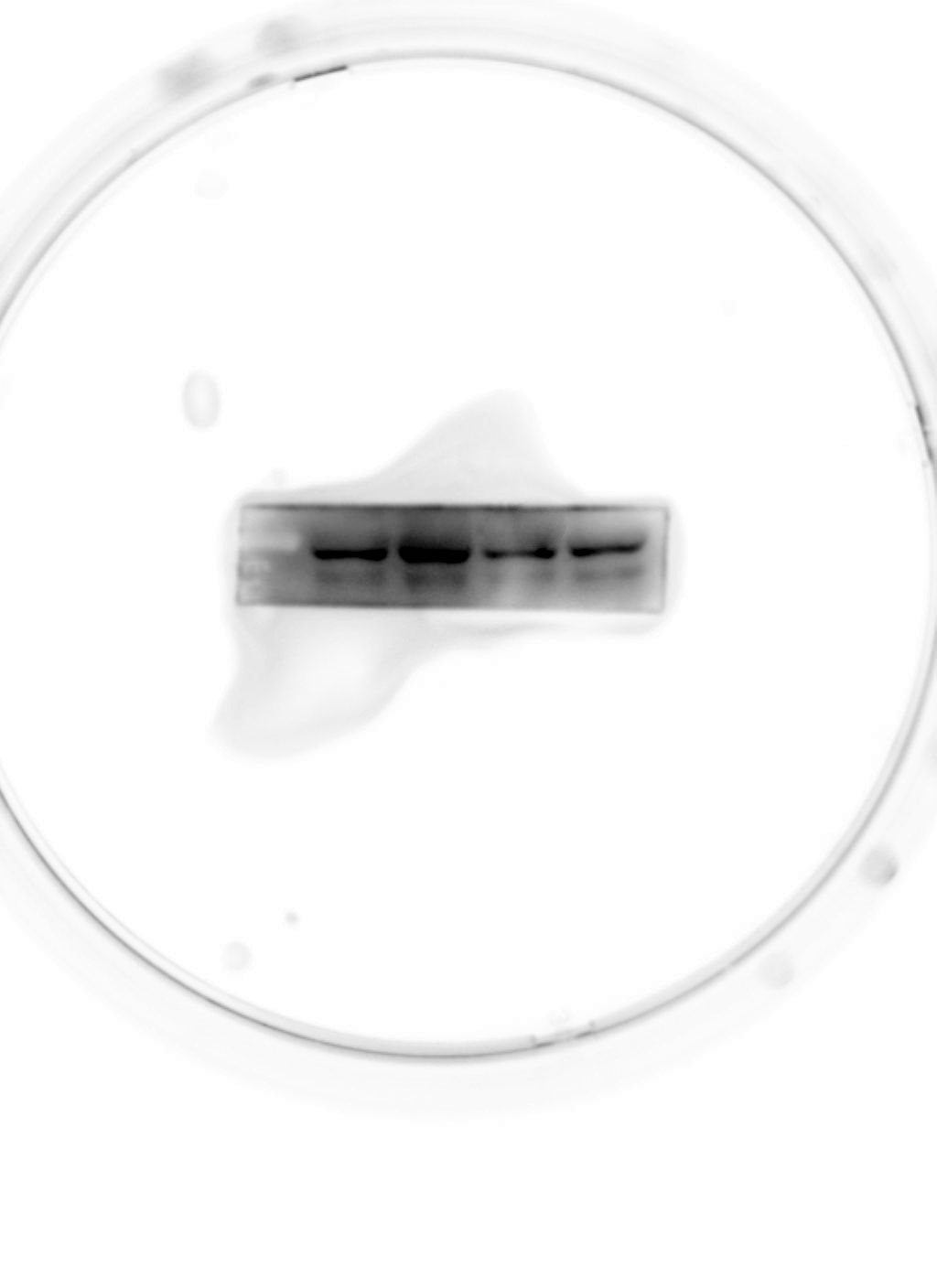

Supplement: Supplementary file 5 [file DataSheet_5.zip › raw original data-Fig6/WB/Fig6C-ERa.jpg]

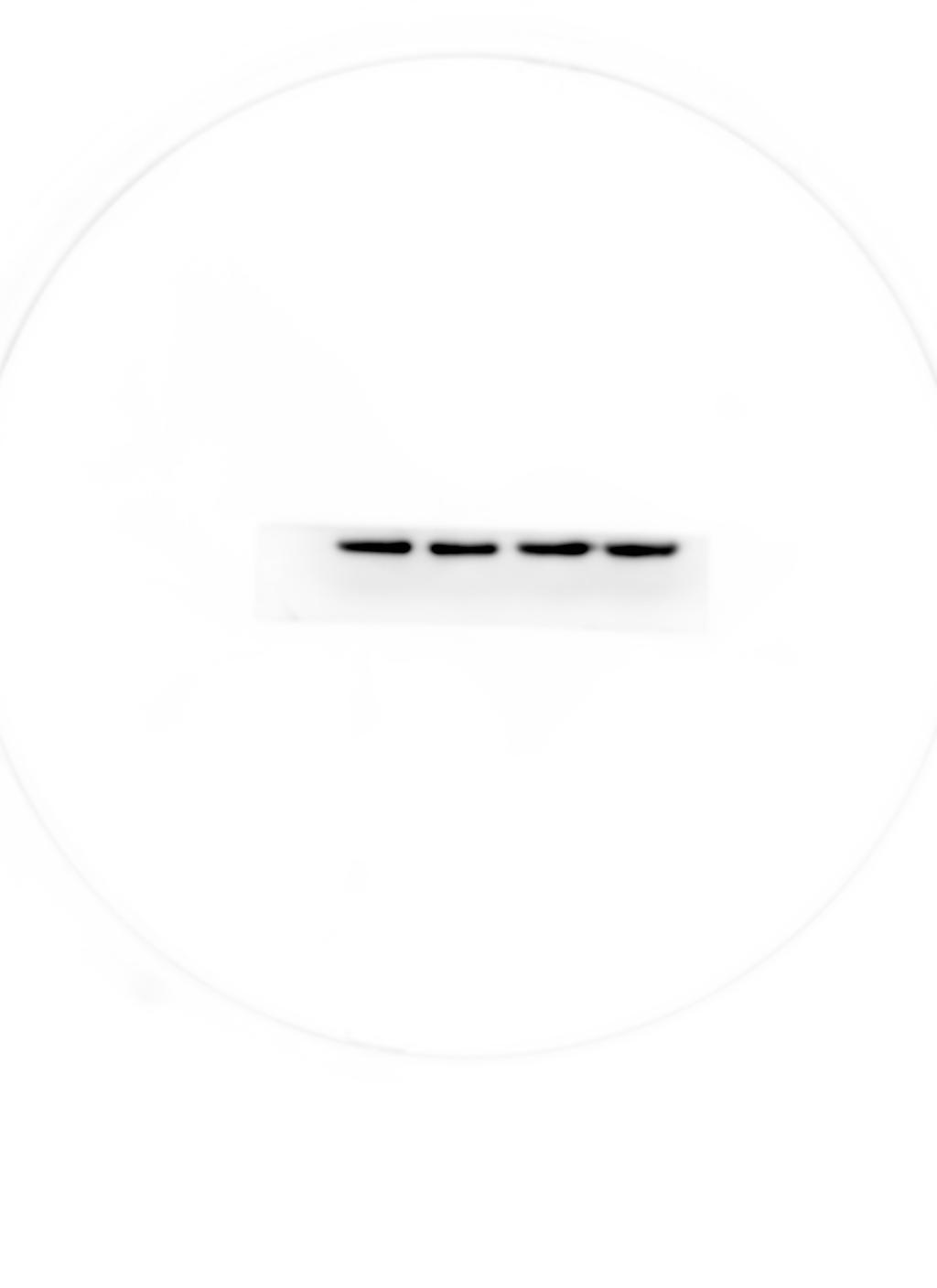

Supplement: Supplementary file 5 [file DataSheet_5.zip › raw original data-Fig6/WB/Fig6C-GAPDH.jpg]

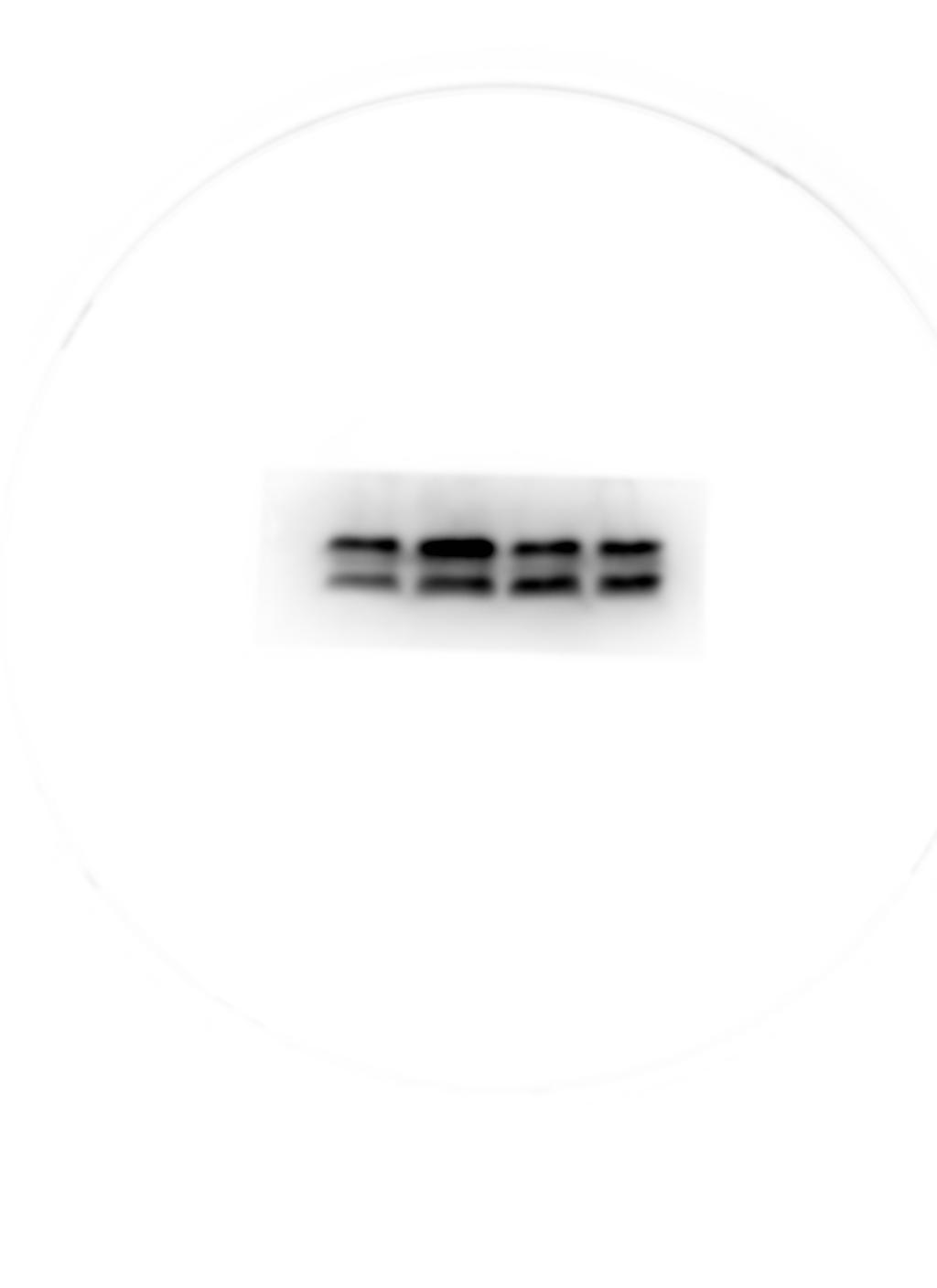

Supplement: Supplementary file 5 [file DataSheet_5.zip › raw original data-Fig6/WB/Fig6C-LC3B.jpg]

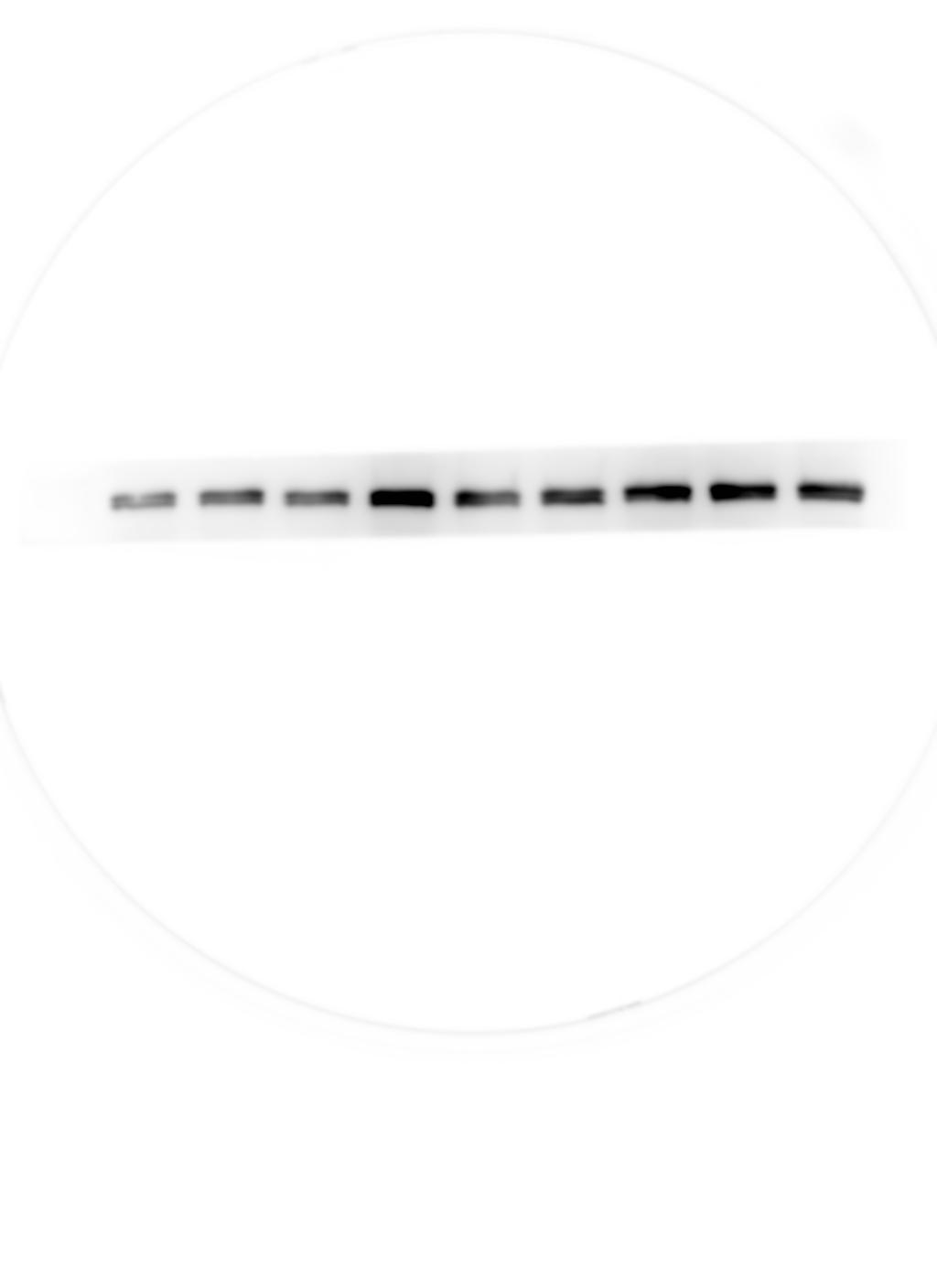

Supplement: Supplementary file 6 [file DataSheet_6.zip › raw original data-Fig2/WB/Fig2E-BECN1.jpg]

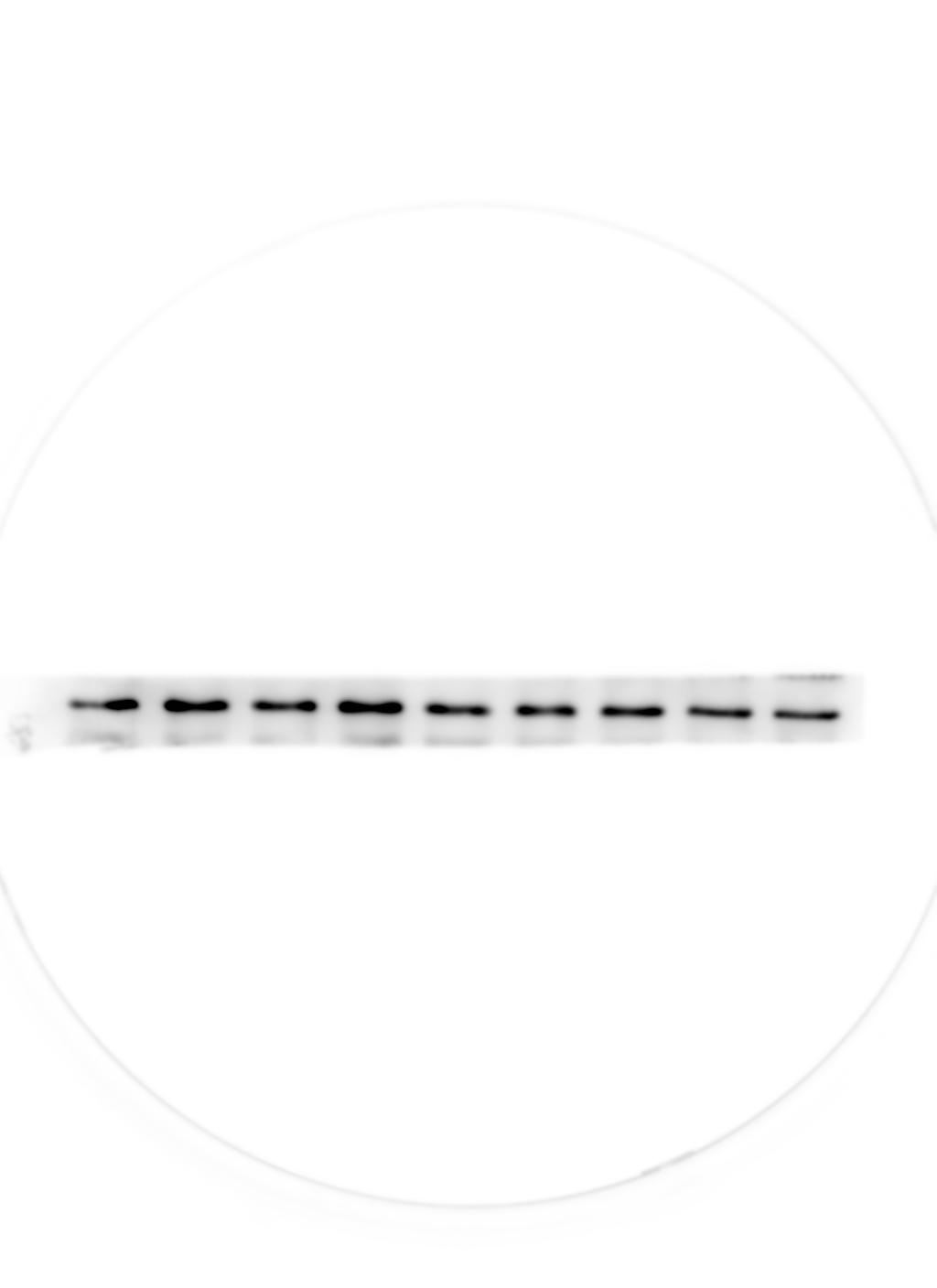

Supplement: Supplementary file 6 [file DataSheet_6.zip › raw original data-Fig2/WB/Fig2E-GAPDH.jpg]

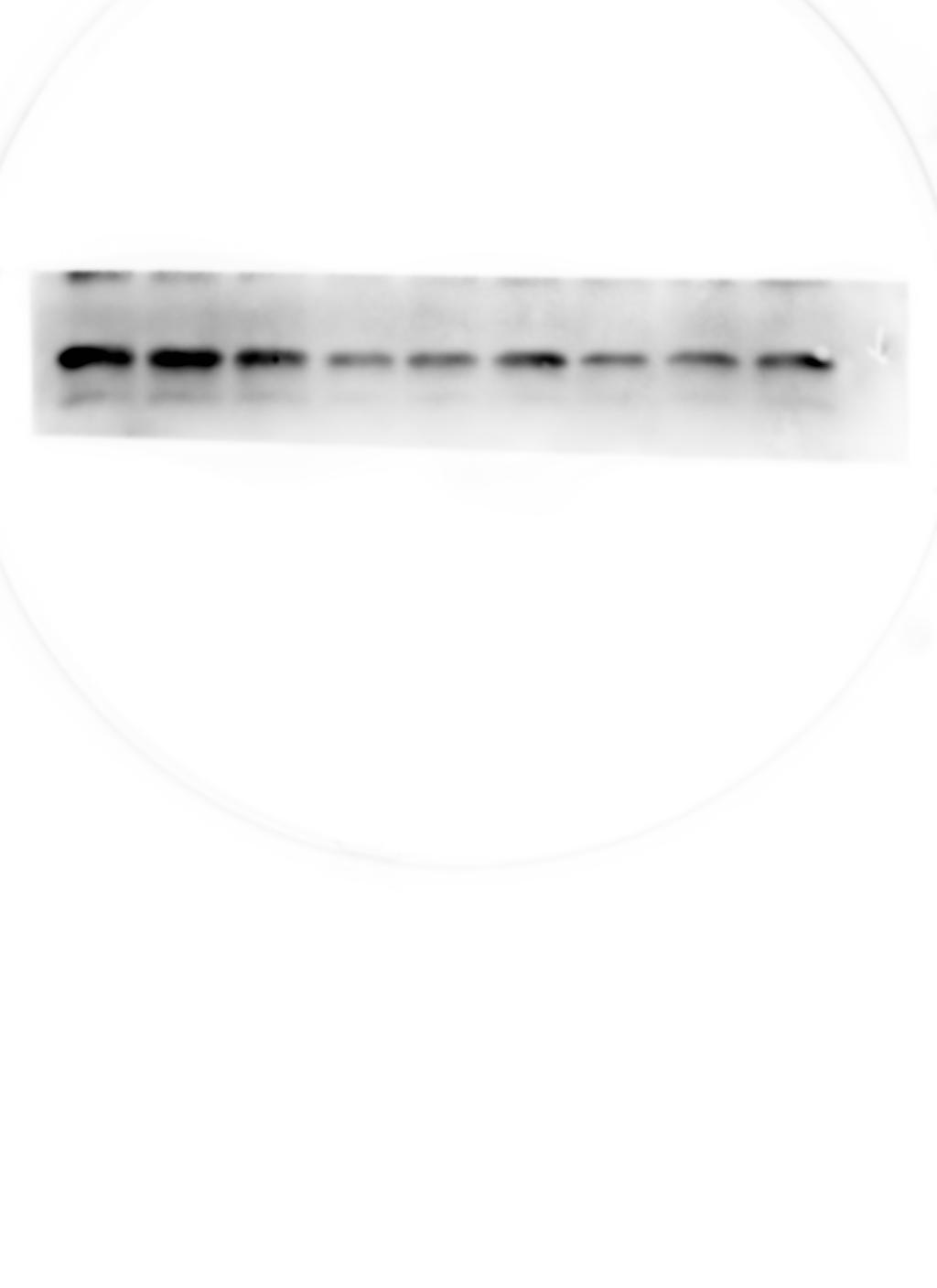

Supplement: Supplementary file 6 [file DataSheet_6.zip › raw original data-Fig2/WB/Fig2E-LC3B.jpg]

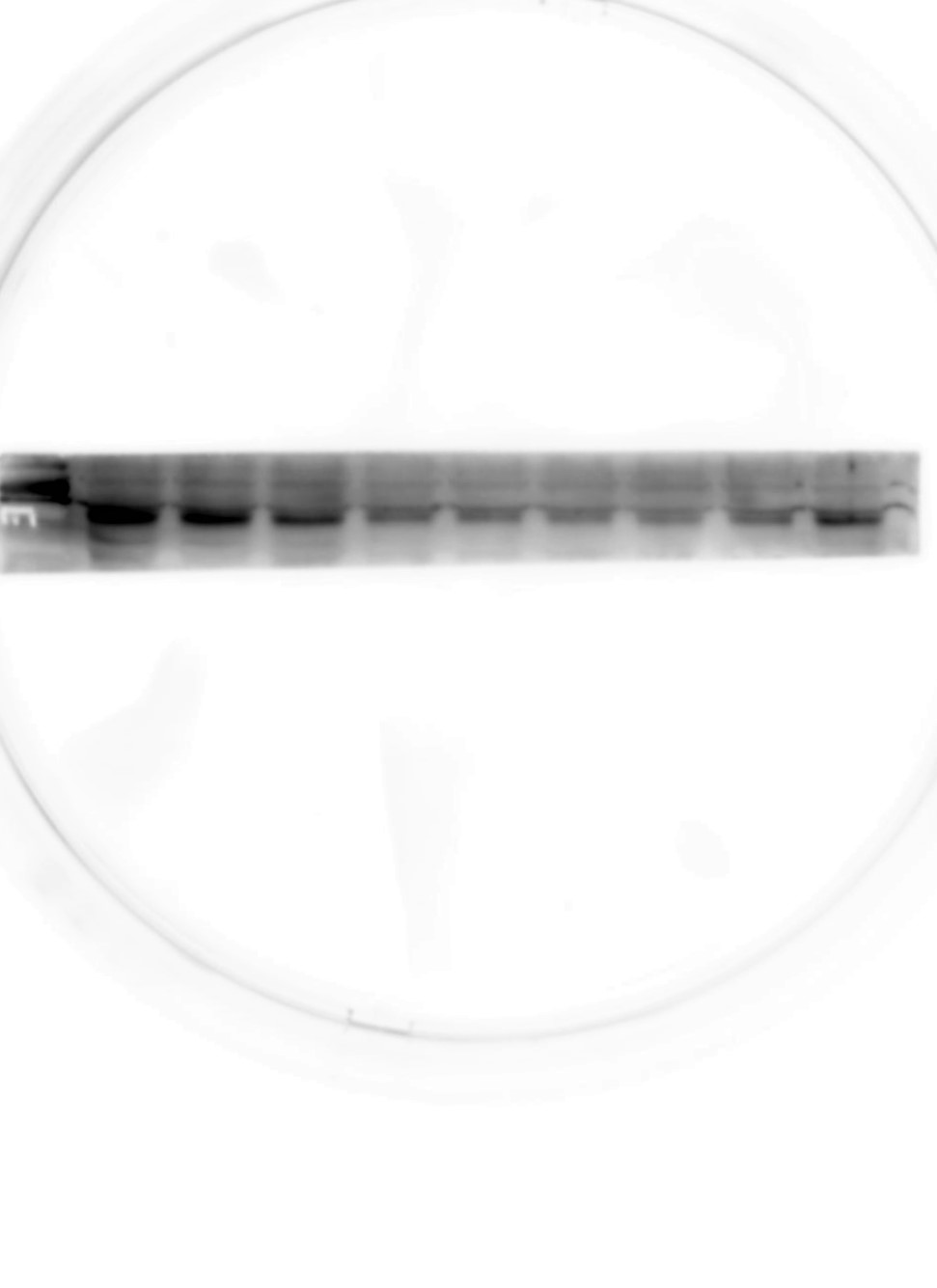

Supplement: Supplementary file 6 [file DataSheet_6.zip › raw original data-Fig2/WB/Fig2F-ERa.jpg]

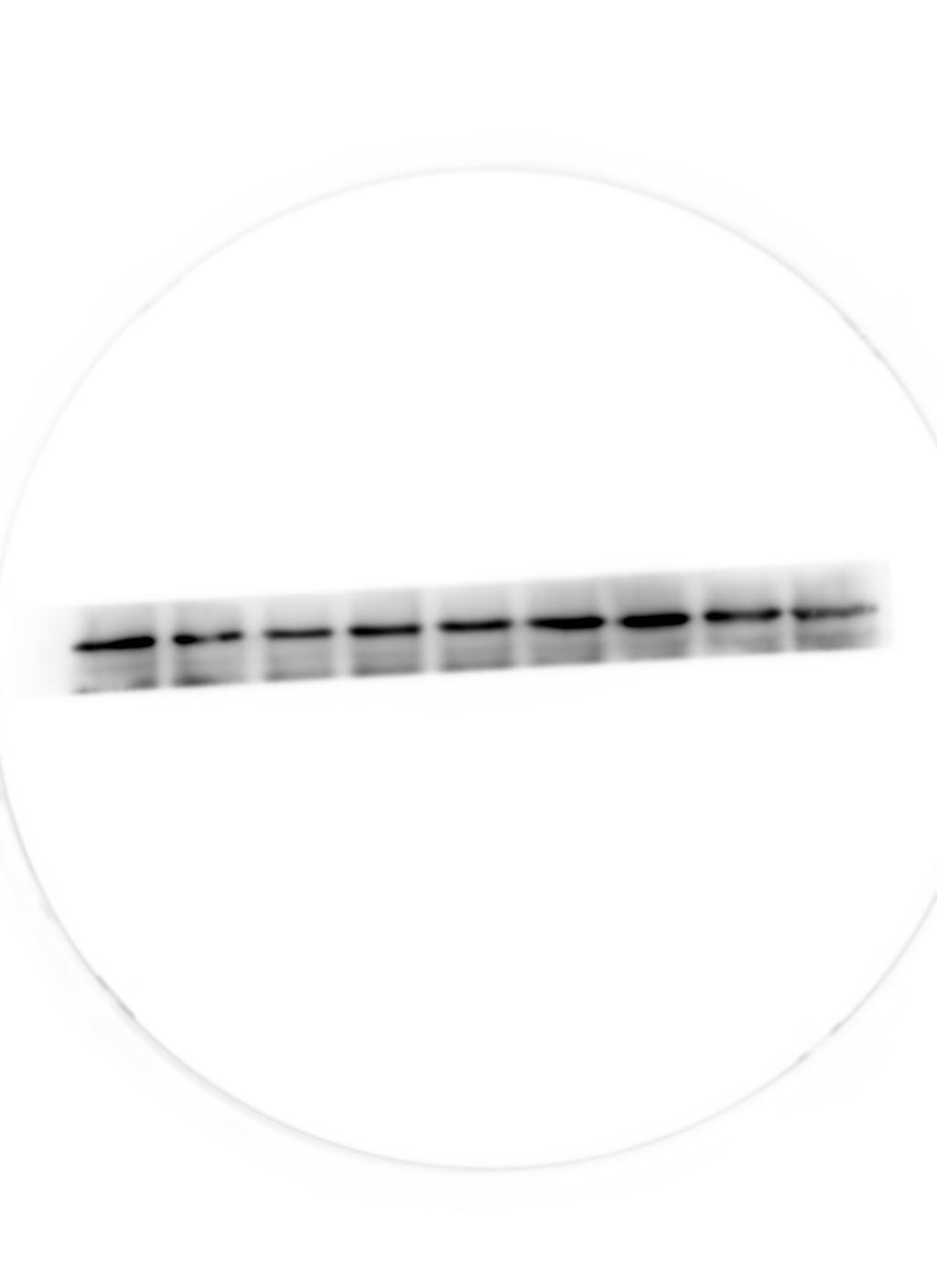

Supplement: Supplementary file 6 [file DataSheet_6.zip › raw original data-Fig2/WB/Fig2F-GAPDH.jpg]

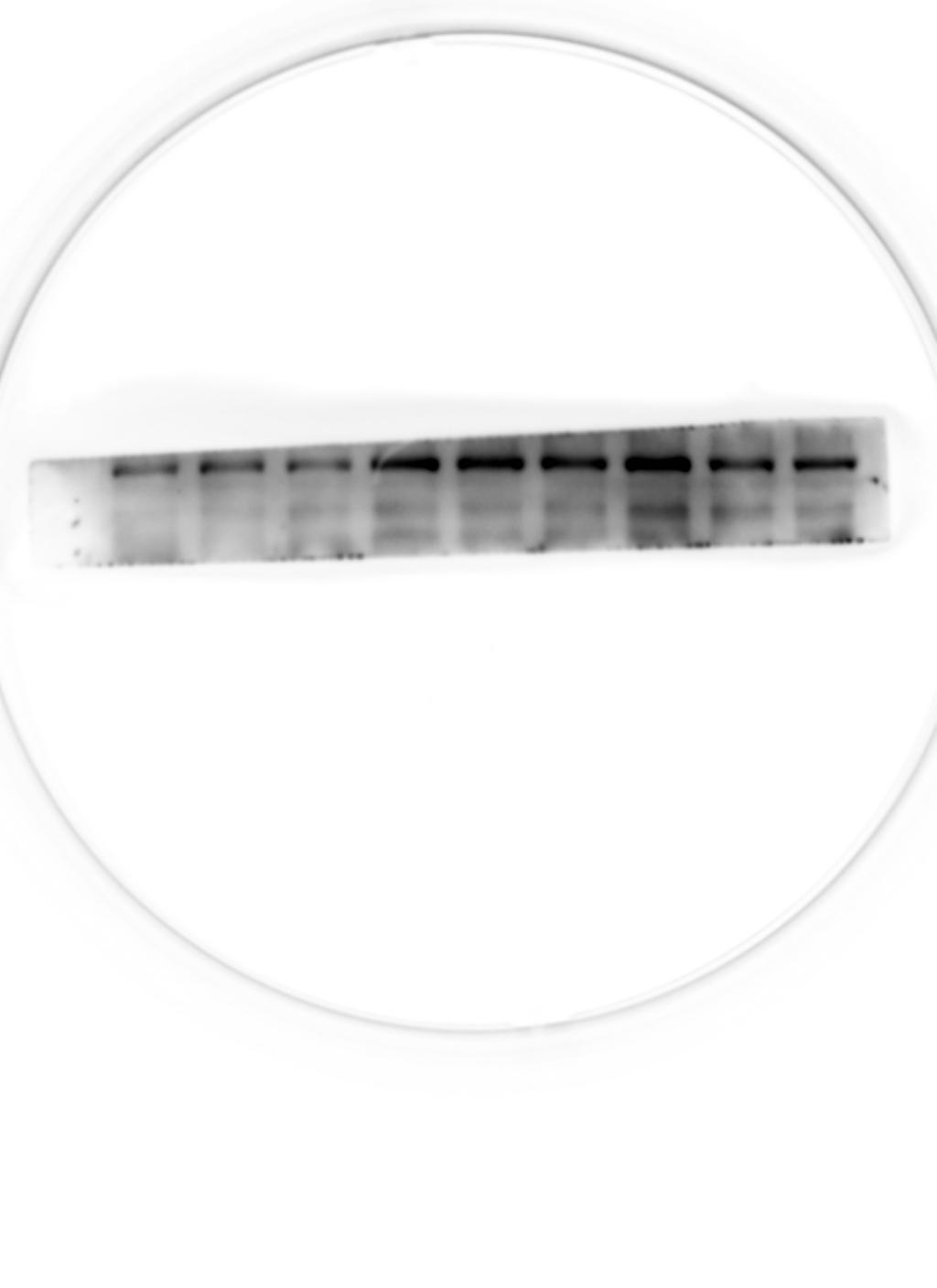

Supplement: Supplementary file 6 [file DataSheet_6.zip › raw original data-Fig2/WB/Fig2F-PR.jpg]

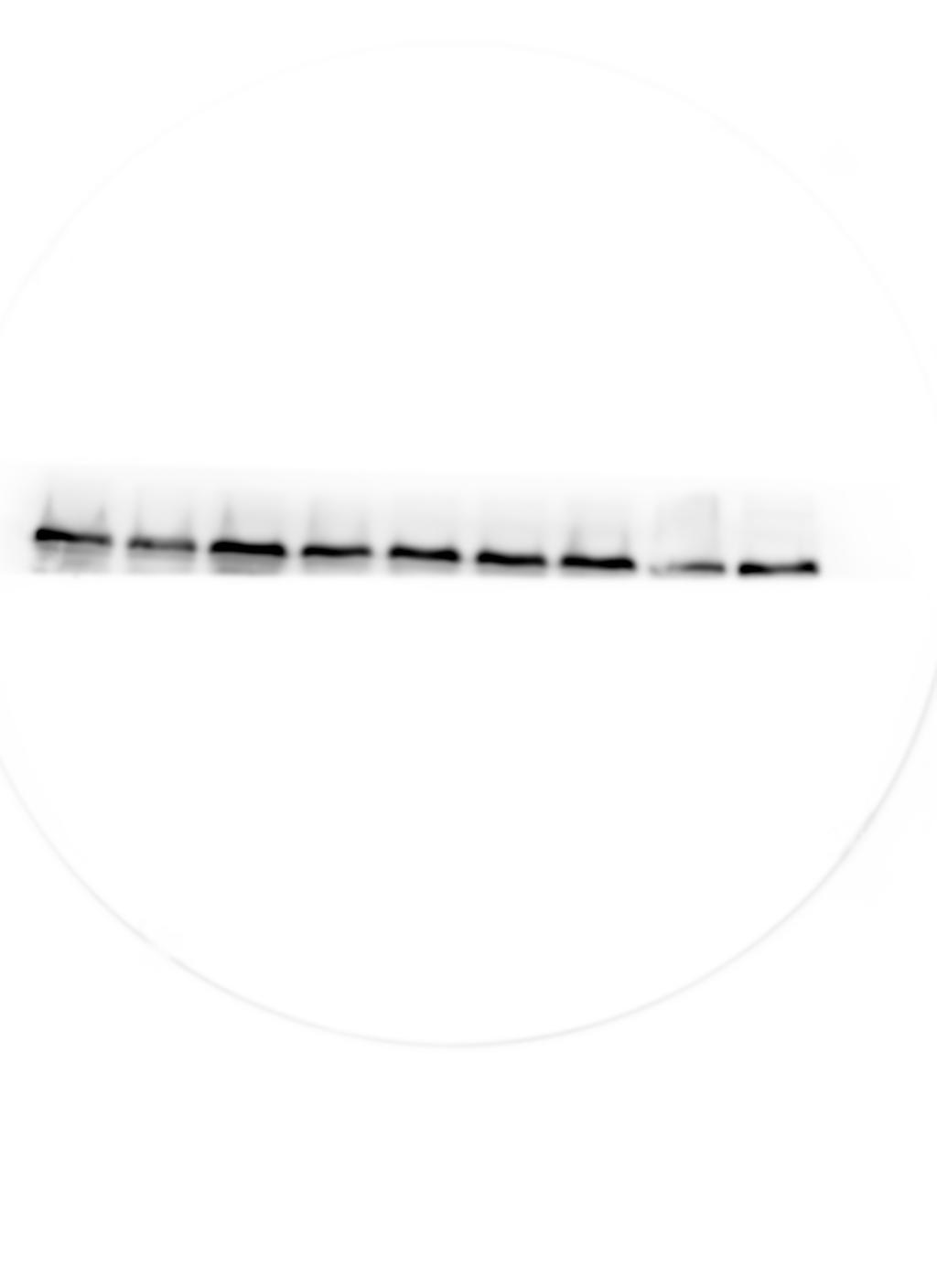

Supplement: Supplementary file 8 [file DataSheet_8.zip › Revised WB raw data/Figure 2F-revised WB/ERβ.jpg]

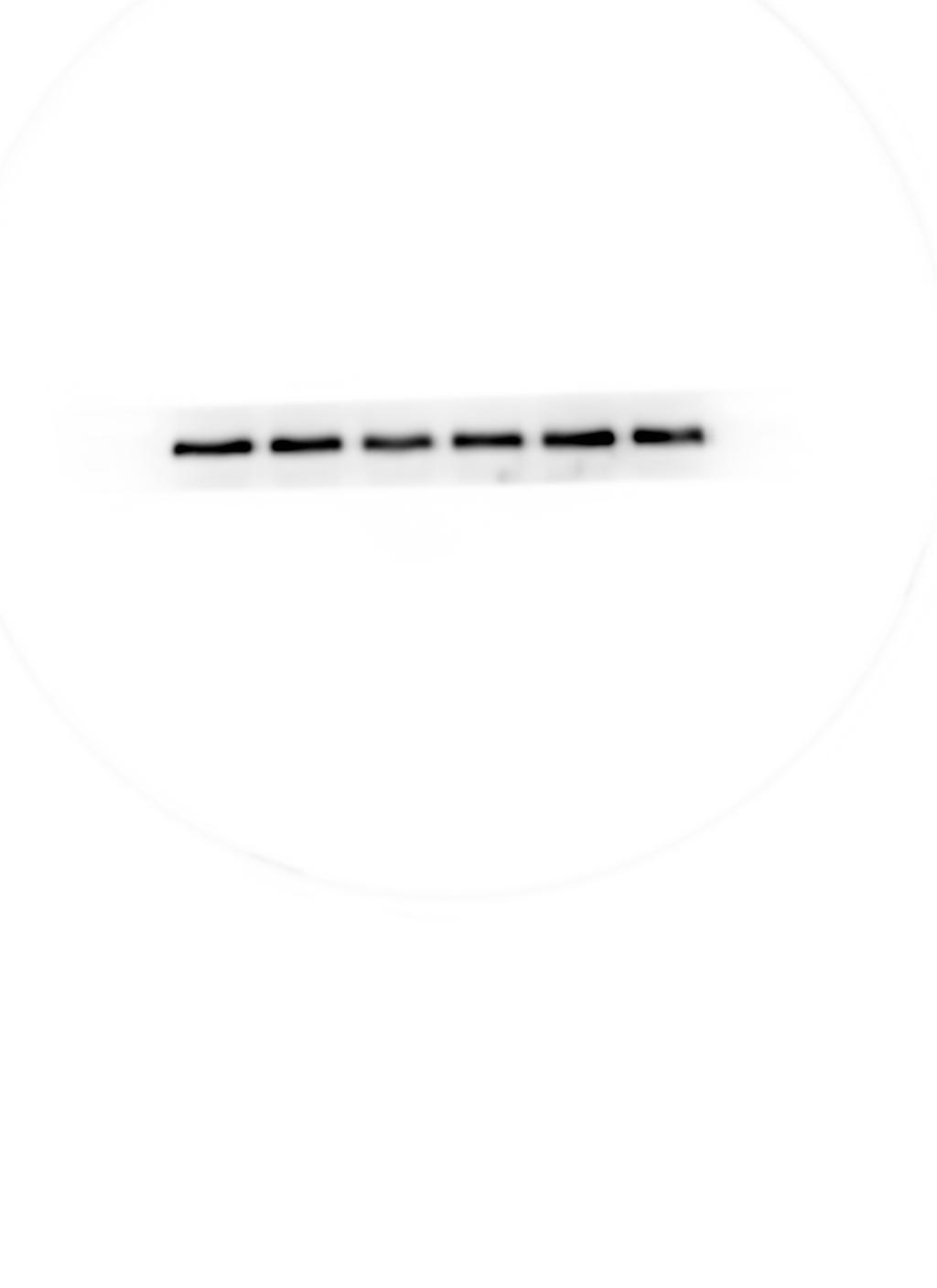

Supplement: Supplementary file 8 [file DataSheet_8.zip › Revised WB raw data/Figure 3B D-revised WB/ERβ-Estrogen.jpg]

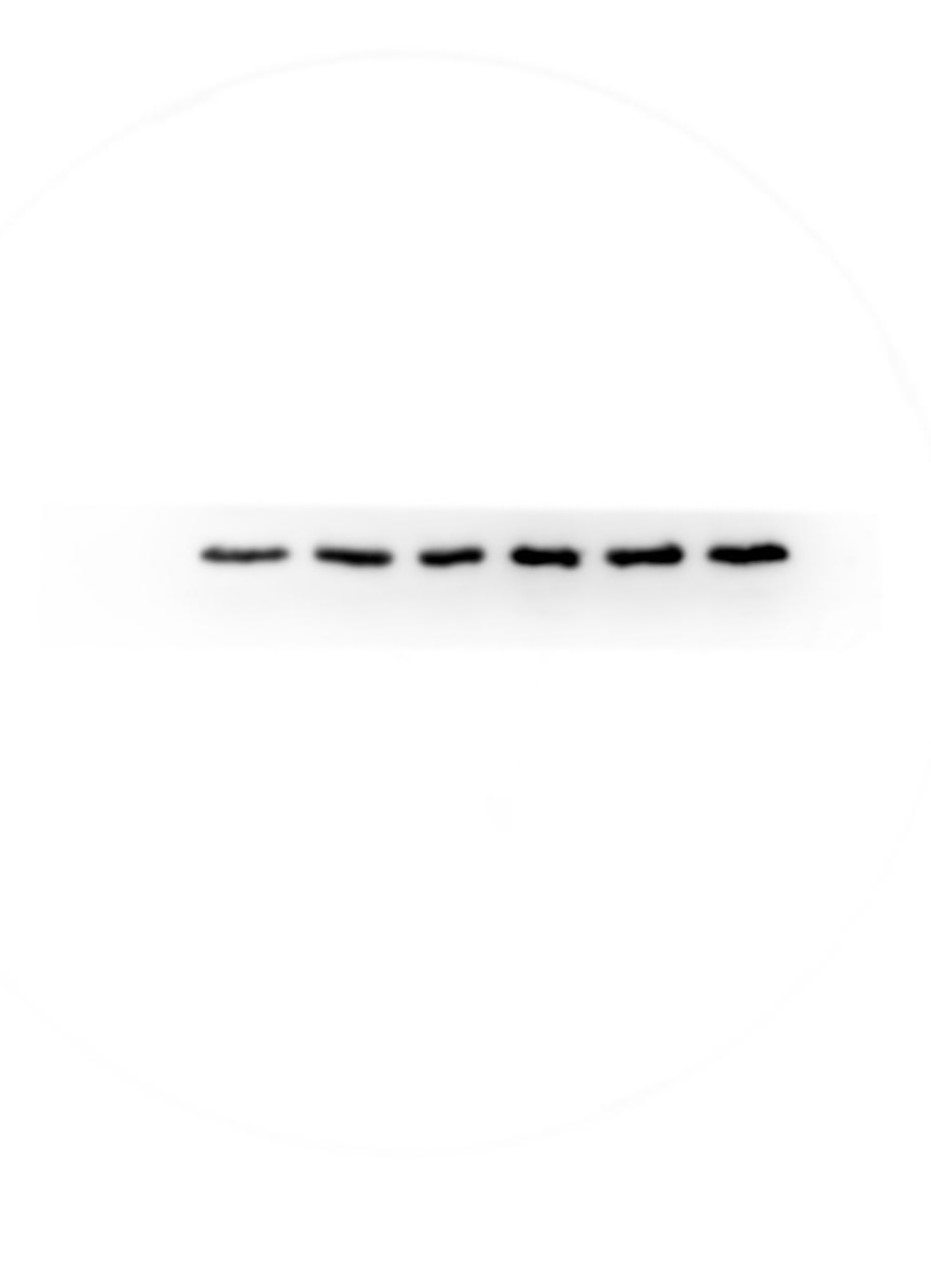

Supplement: Supplementary file 8 [file DataSheet_8.zip › Revised WB raw data/Figure 3B D-revised WB/ERβ-neESC.jpg]

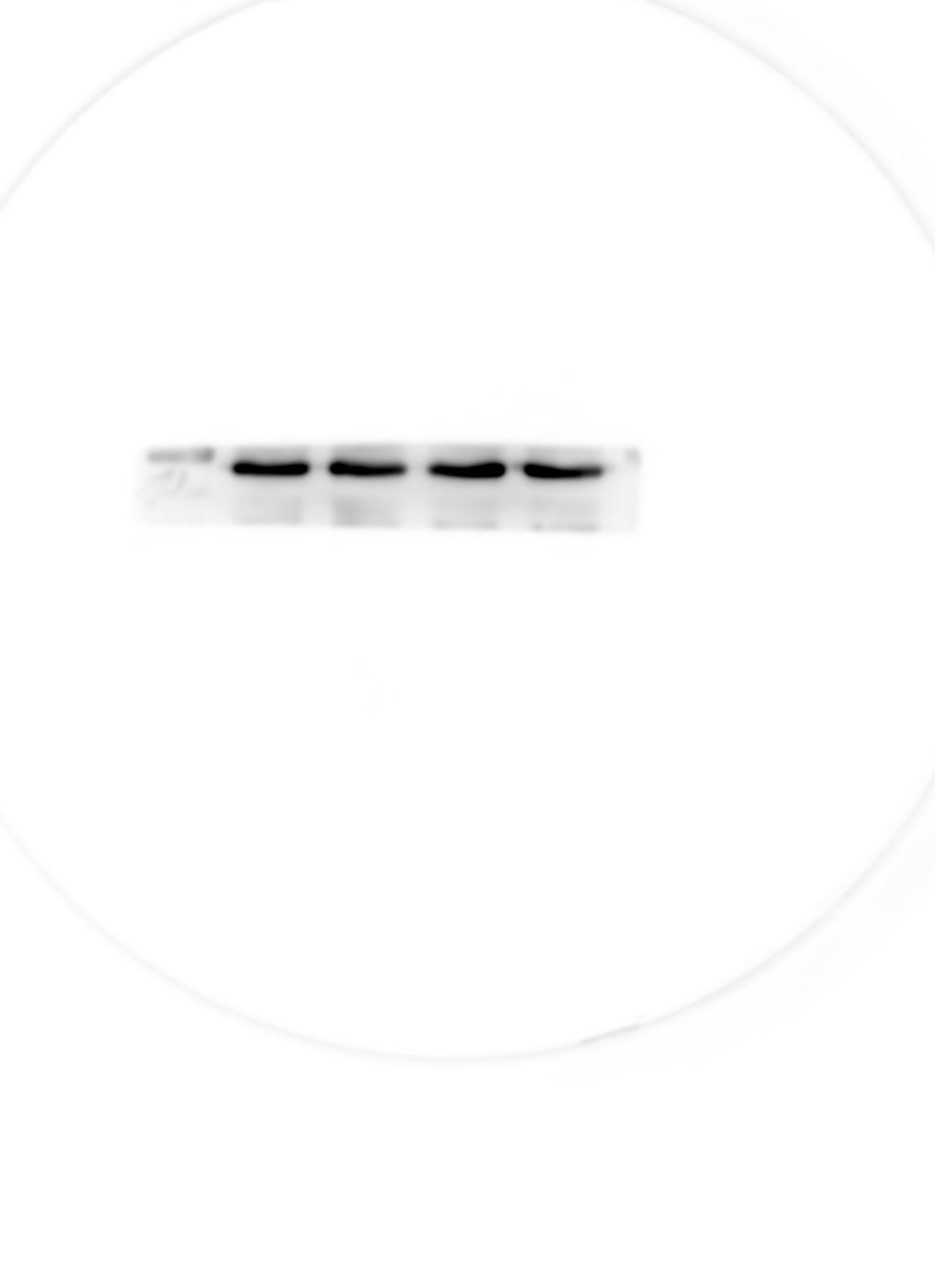

Supplement: Supplementary file 8 [file DataSheet_8.zip › Revised WB raw data/Figure 4B C-revised WB/ERβ SCM-198 E2.jpg]

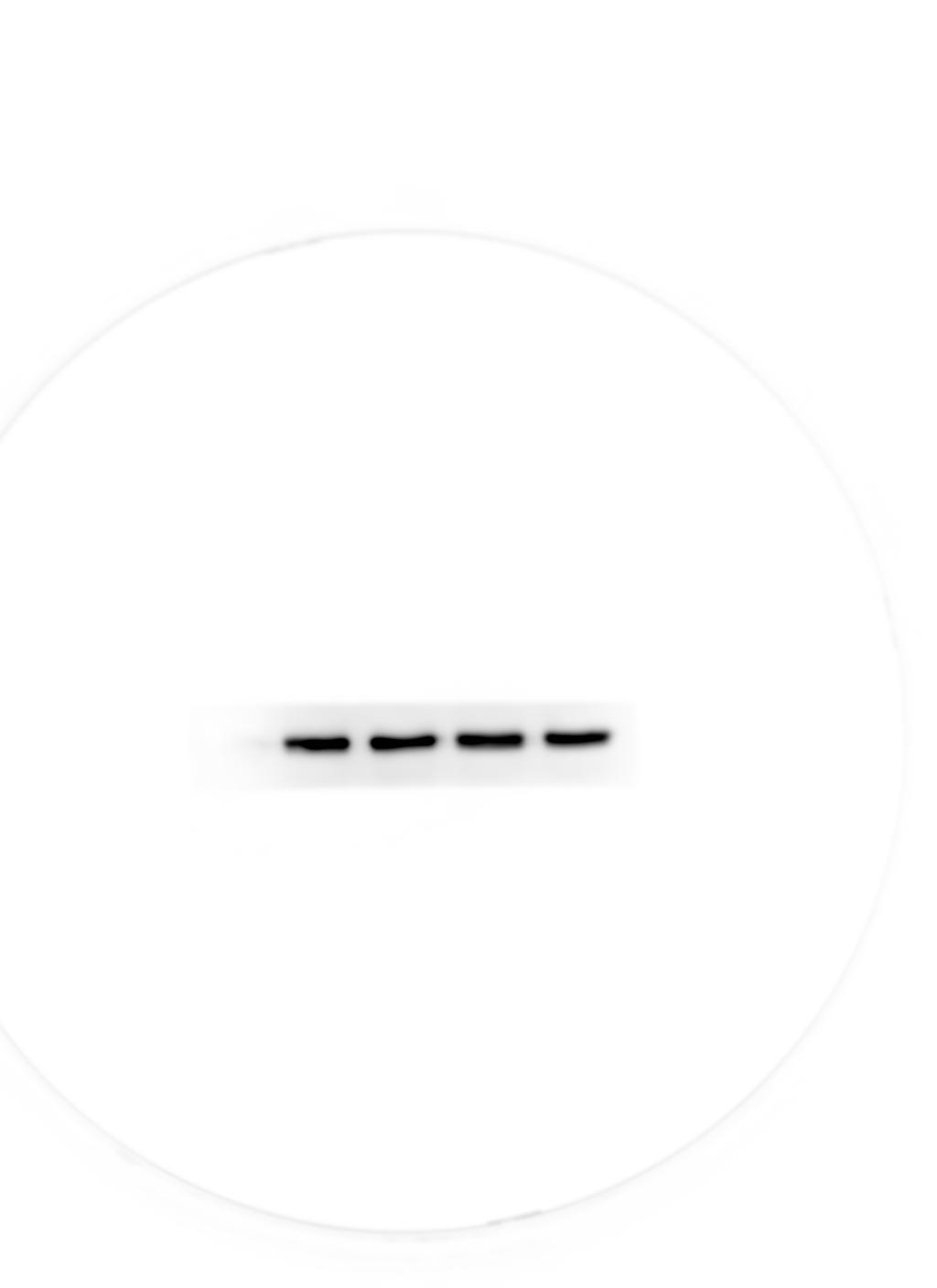

Supplement: Supplementary file 8 [file DataSheet_8.zip › Revised WB raw data/Figure 4B C-revised WB/ERβ SCM-198.jpg]

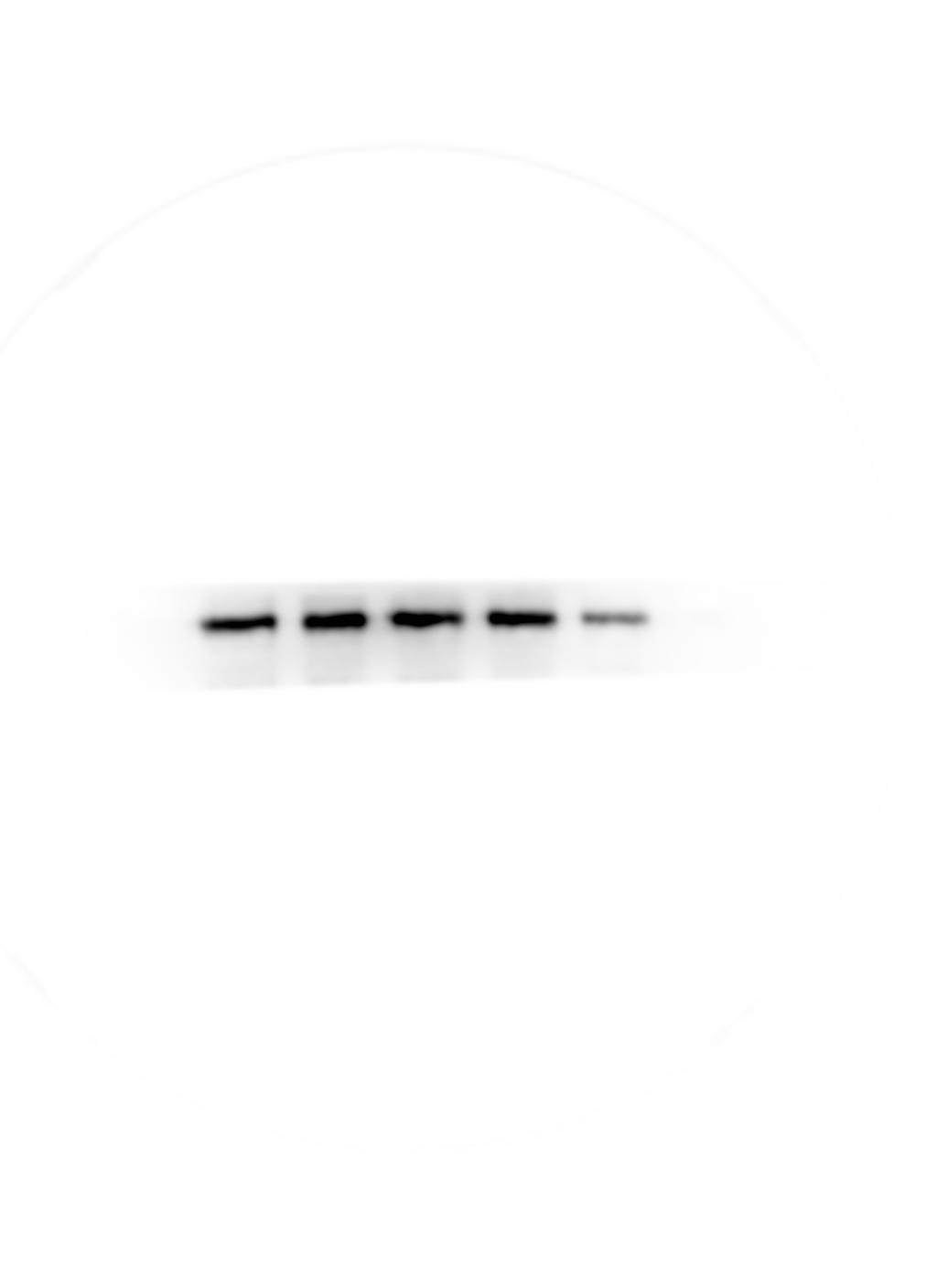

Supplement: Supplementary file 8 [file DataSheet_8.zip › Revised WB raw data/Figure 5C-revised WB/ERβ .jpg]

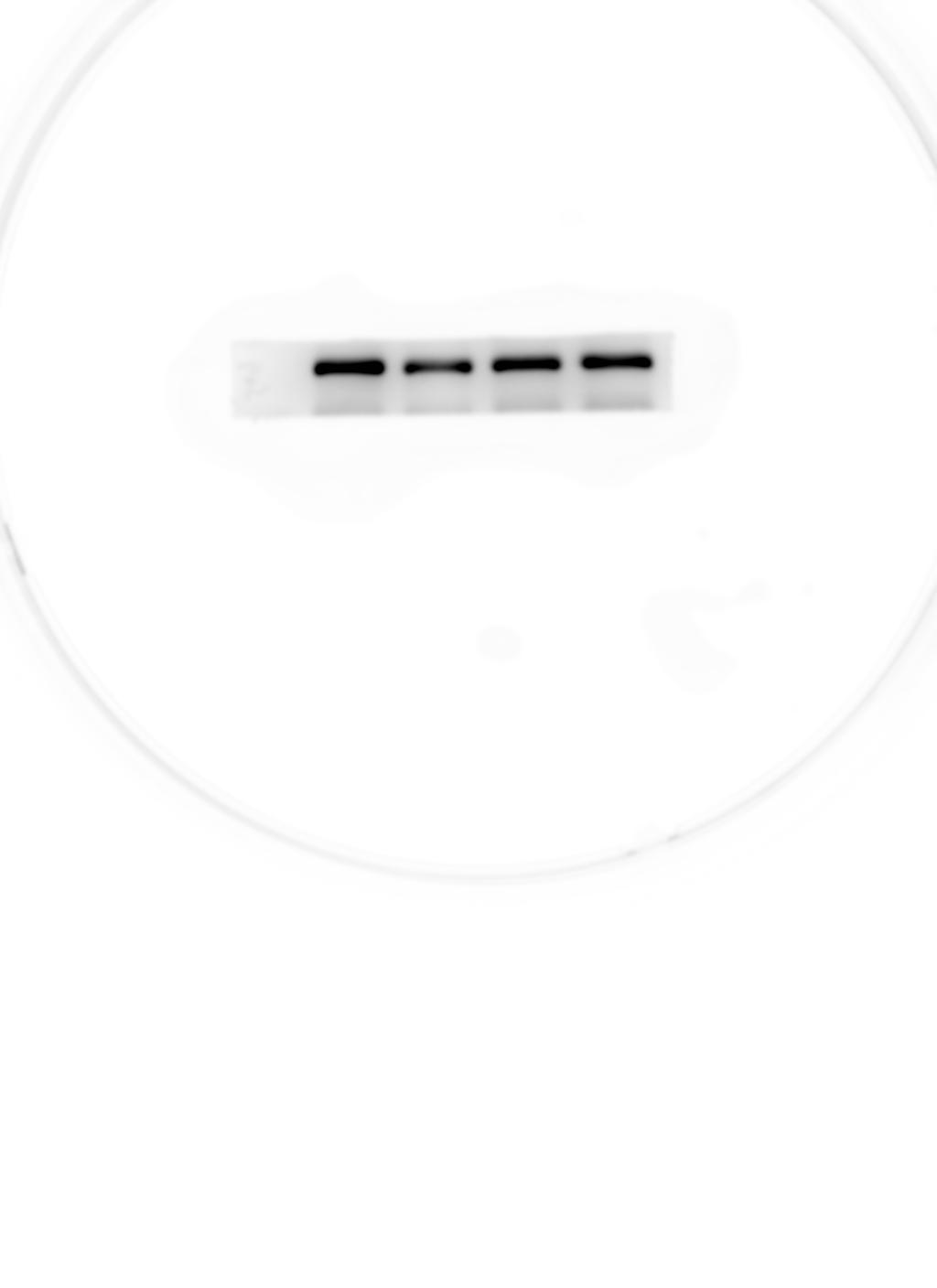

Supplement: Supplementary file 8 [file DataSheet_8.zip › Revised WB raw data/Figure 5C-revised WB/PRB.jpg]

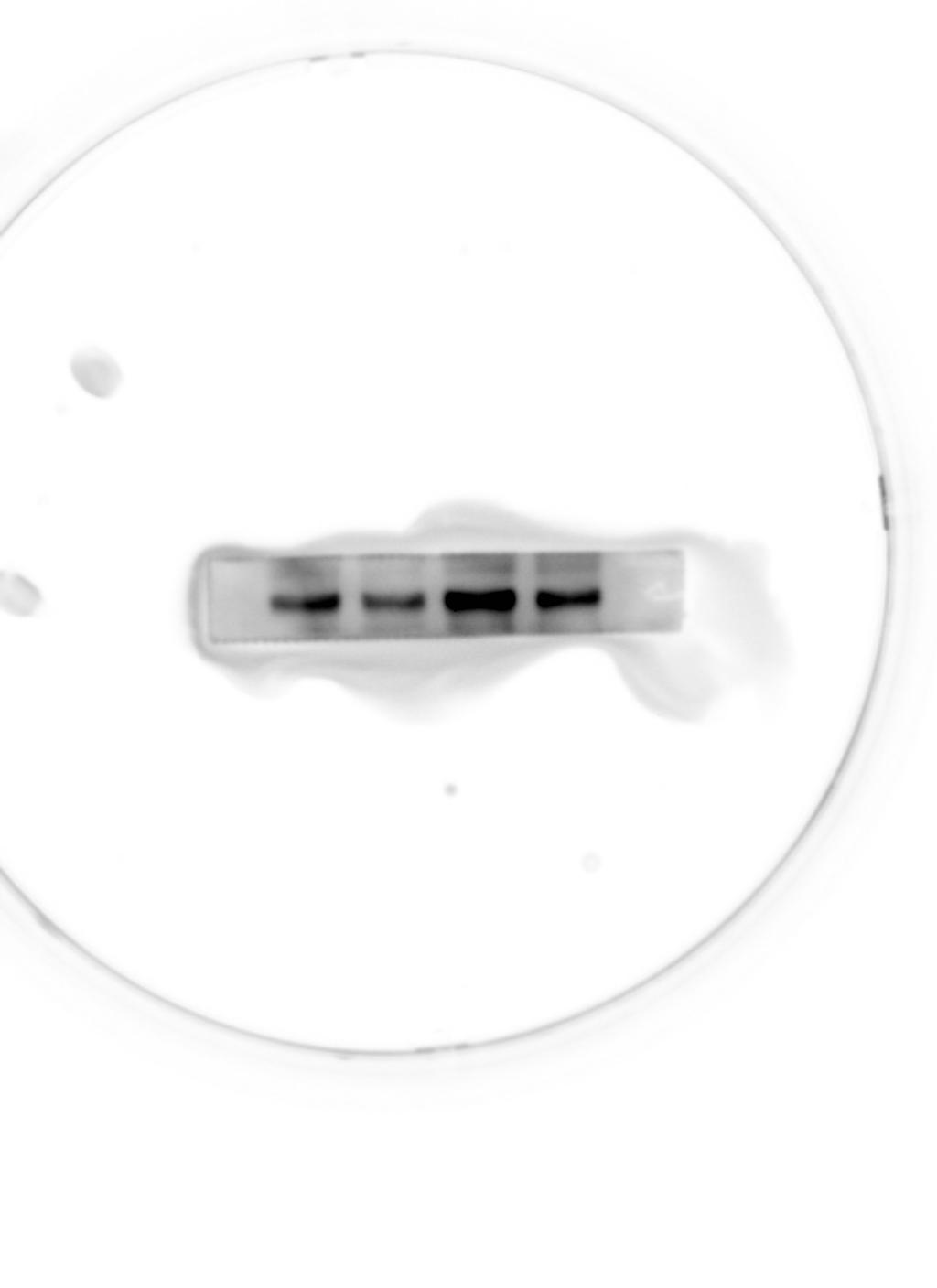

Supplement: Supplementary file 8 [file DataSheet_8.zip › Revised WB raw data/Figure 6C-revised WB/PRB.jpg]
